# Supplementary figures and images for: Generalist Eimeria species in rodents: Multilocus analyses indicate inadequate resolution of established markers
Source: Ecol Evol. 2020 Jan 11;10(3):1378–89. doi: 10.1002/ece3.5992 (PMC7029063; doi:10.1002/ece3.5992)

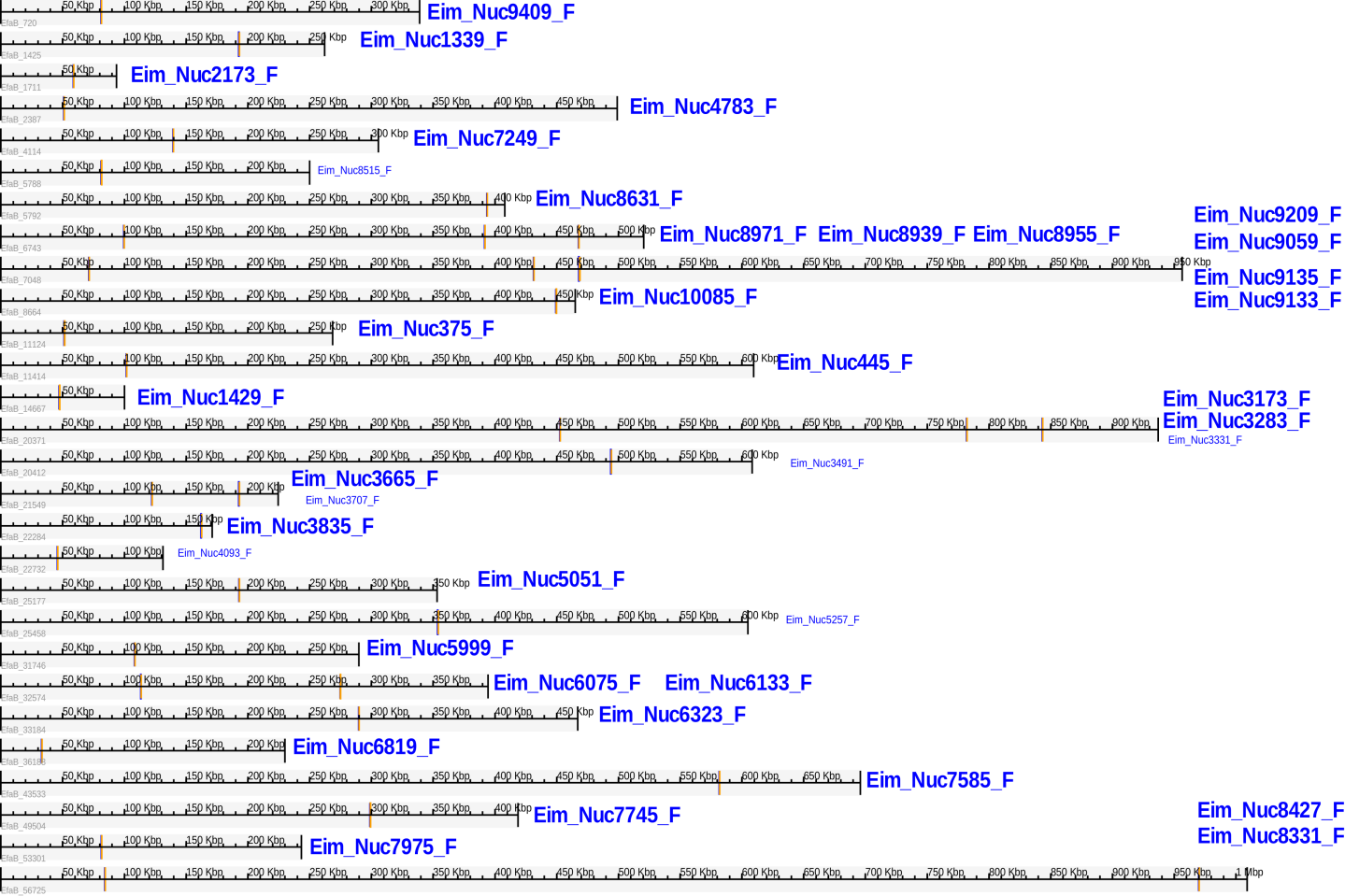

Supplement: Supplementary file 4 [file ECE3-10-1378-s004.pdf]

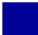 Coding sequence (CDS)

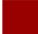 tRNA

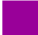 rRNA

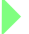 Primer position

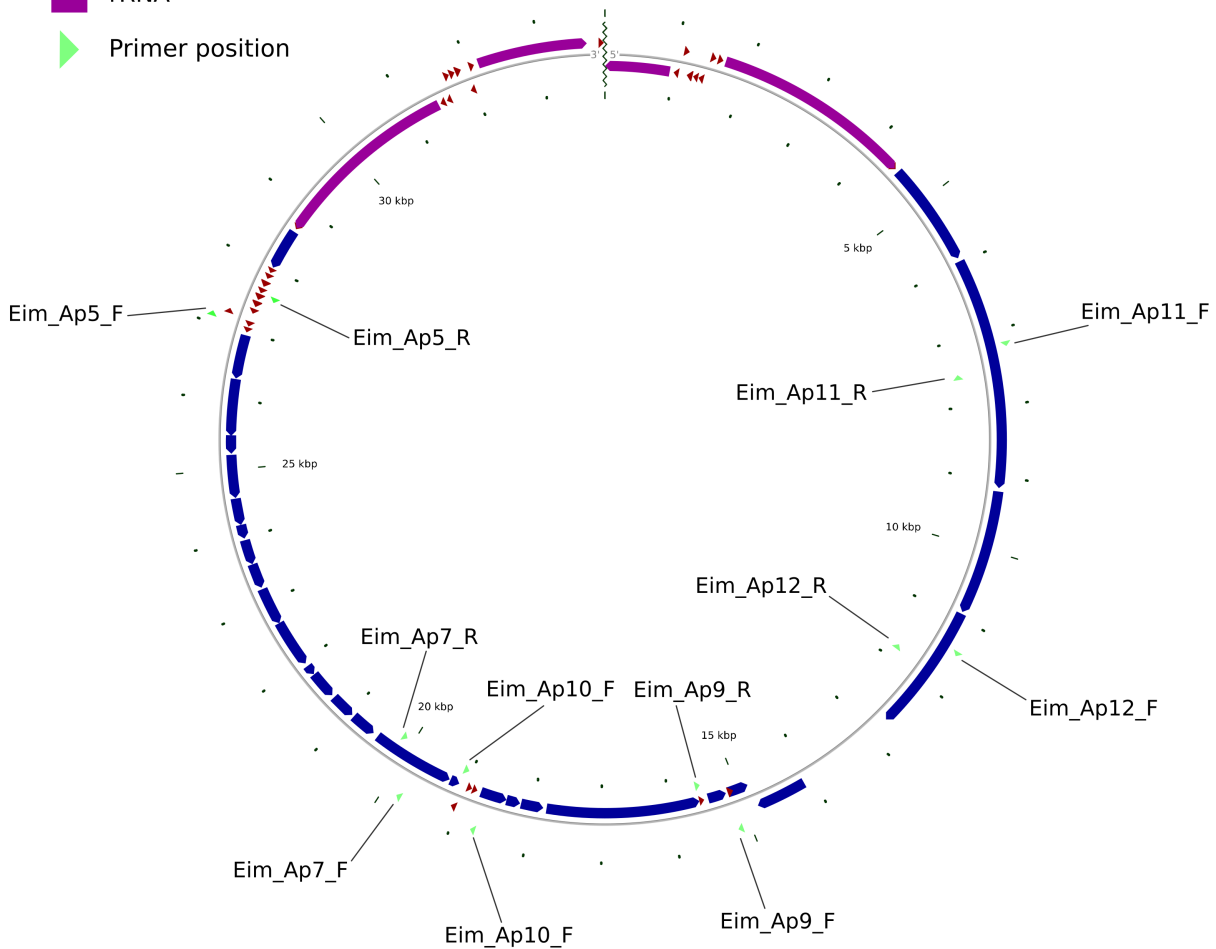

Supplement: Supplementary file 5 [file ECE3-10-1378-s005.pdf]

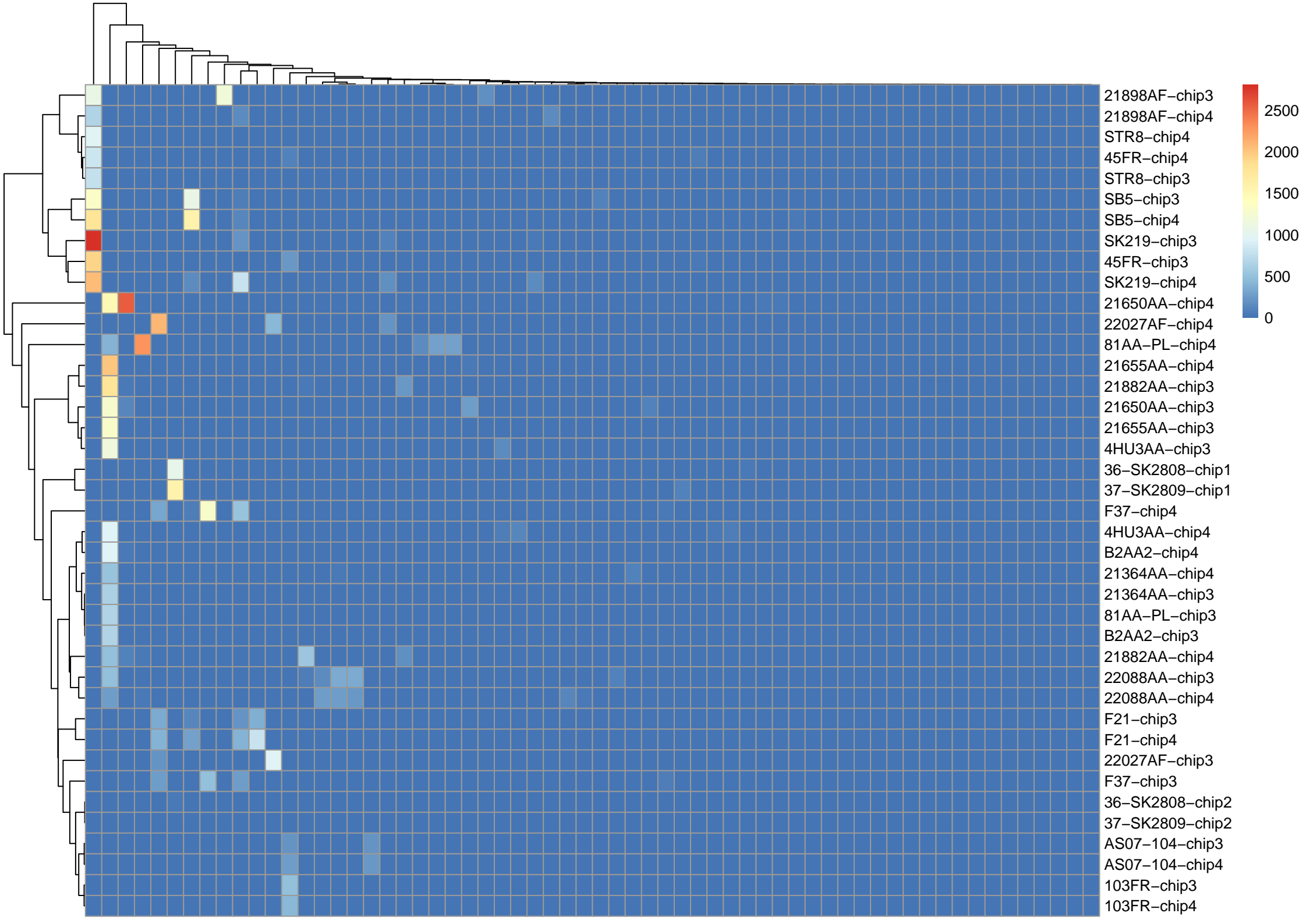

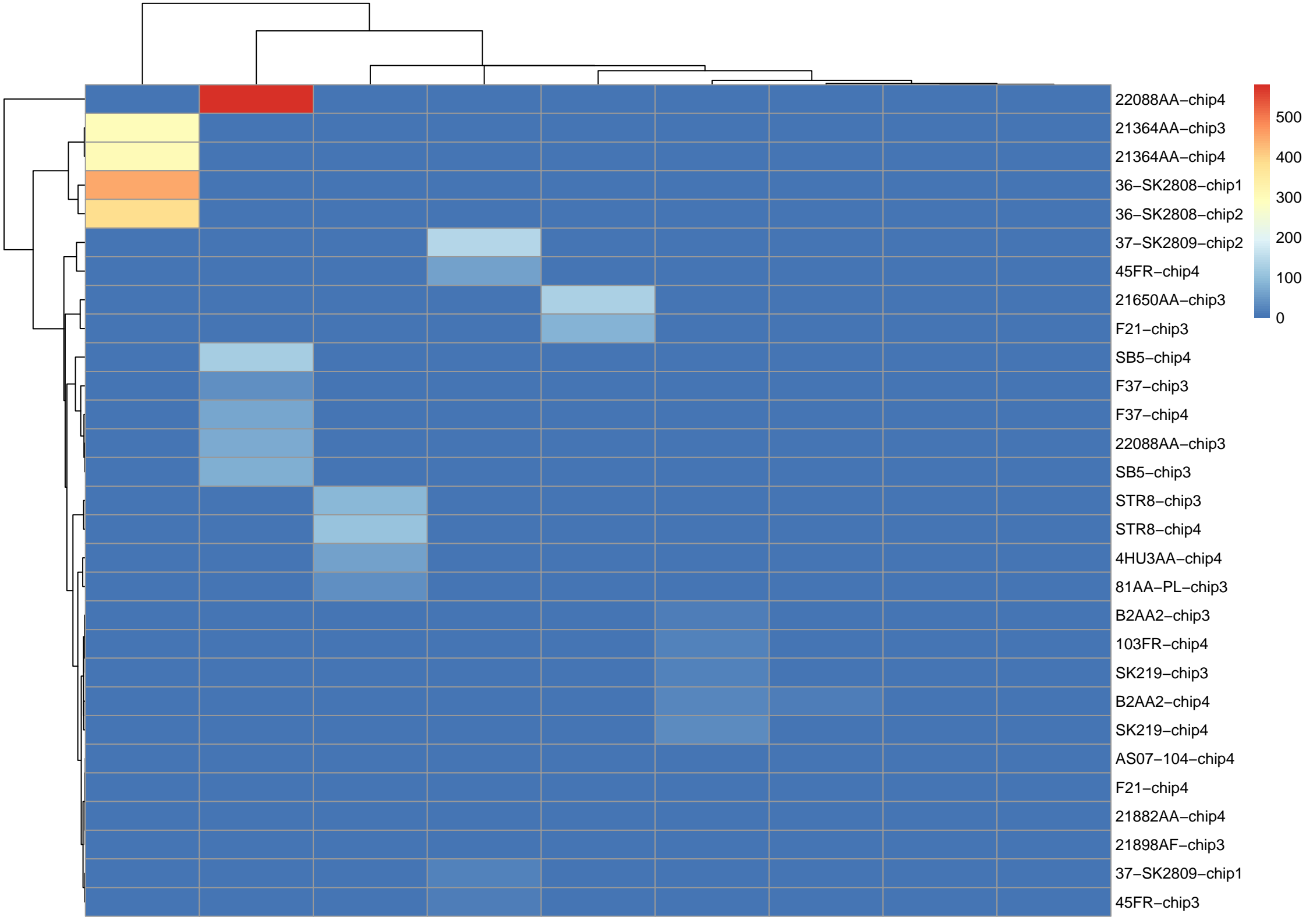

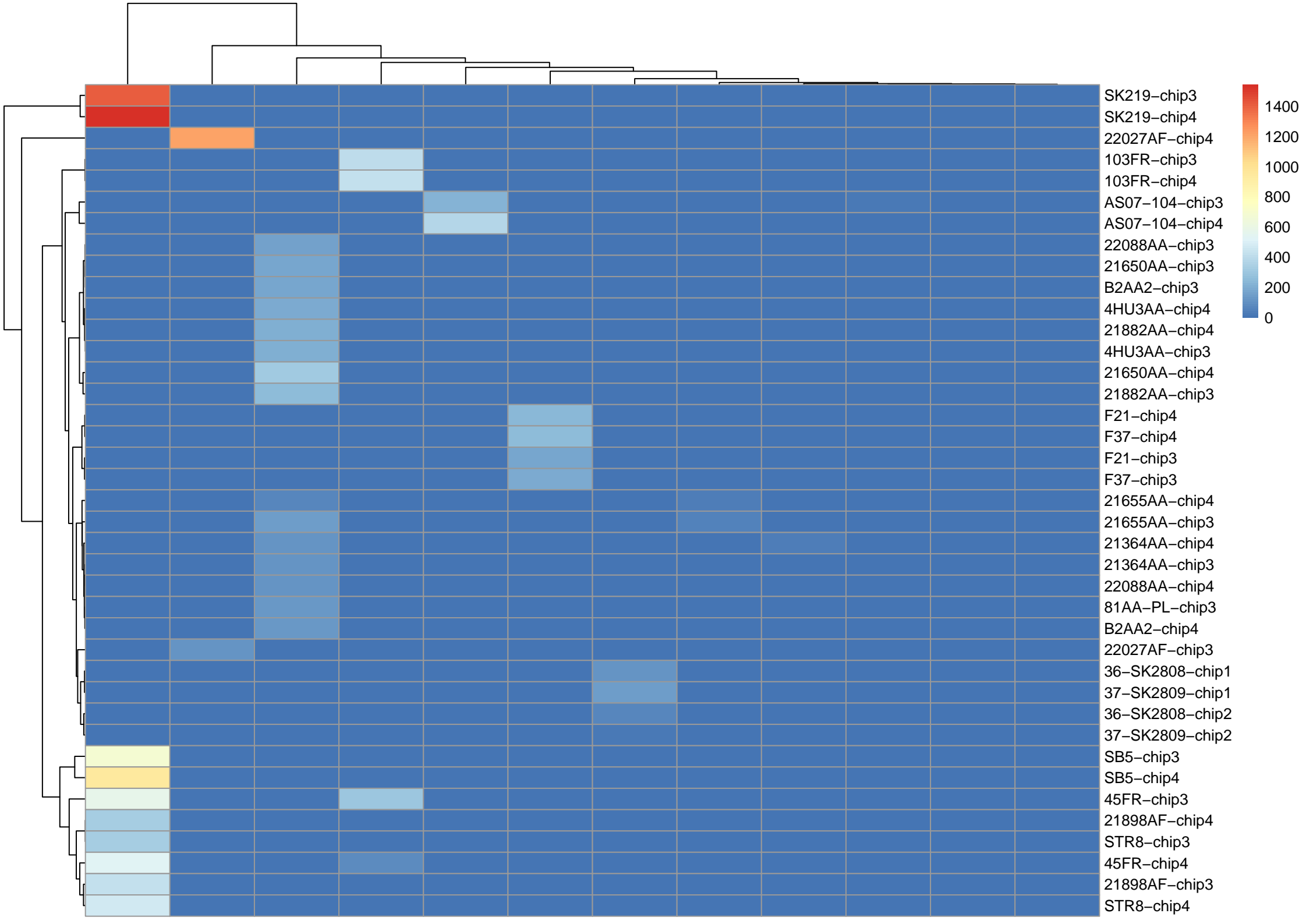

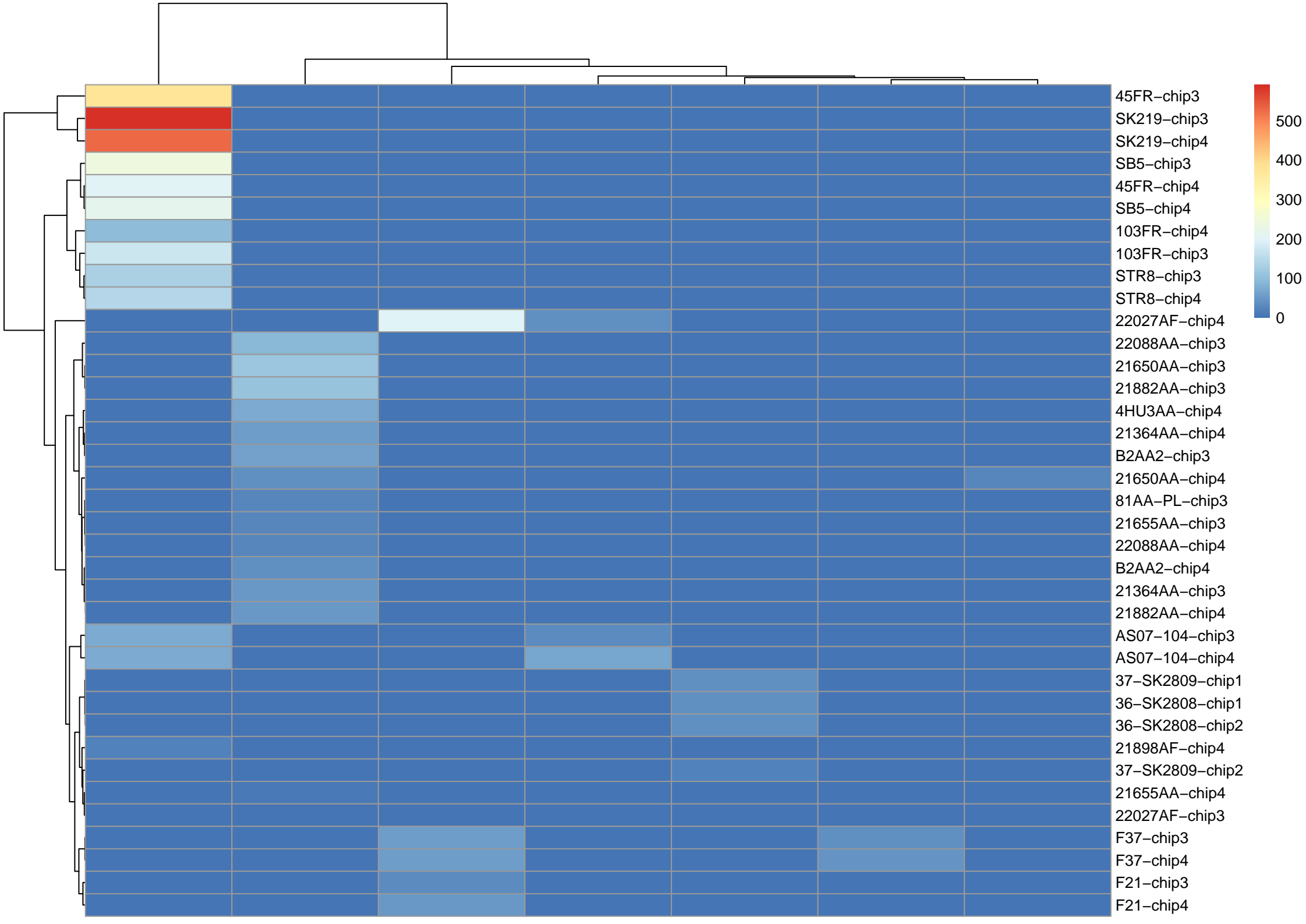

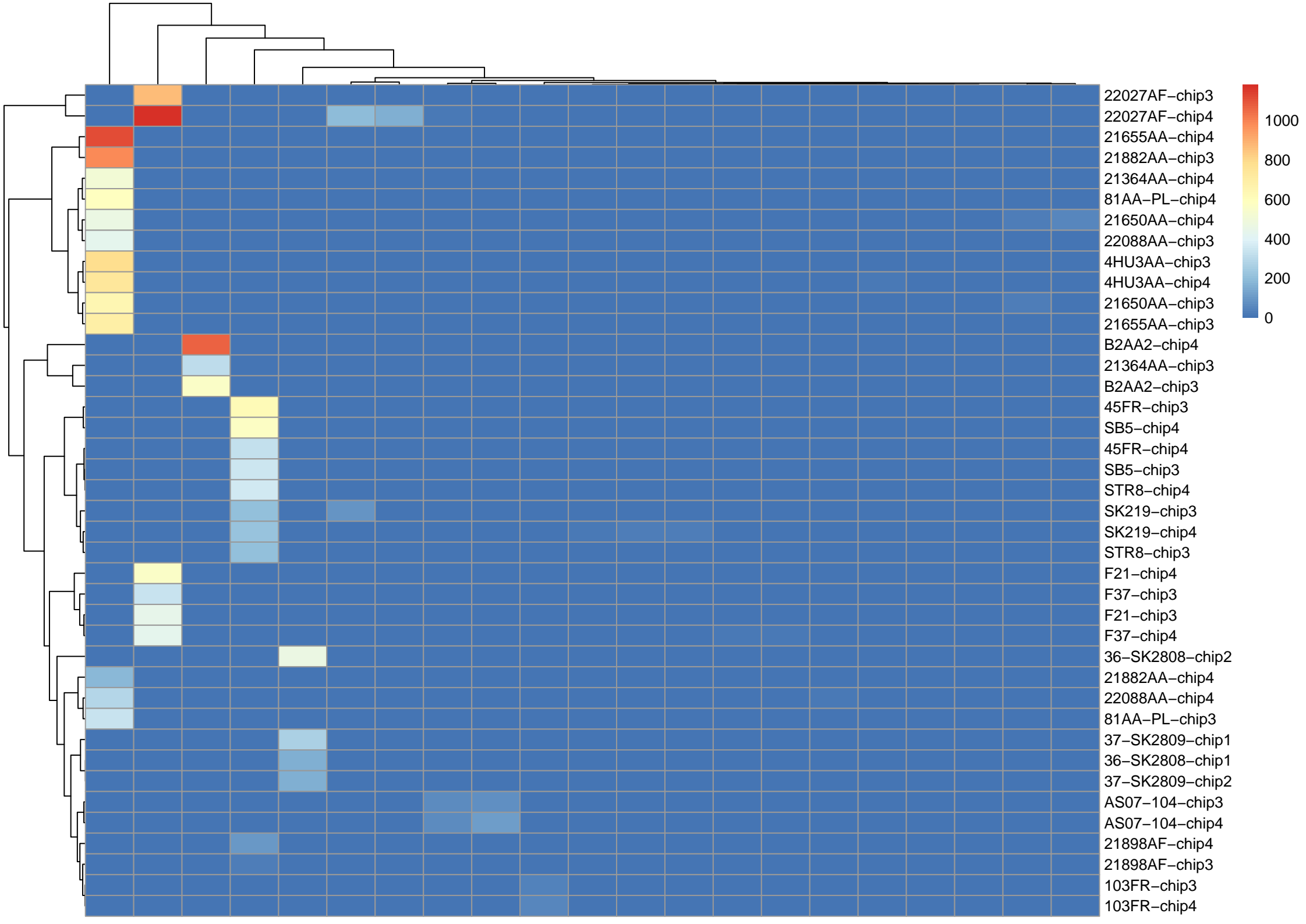

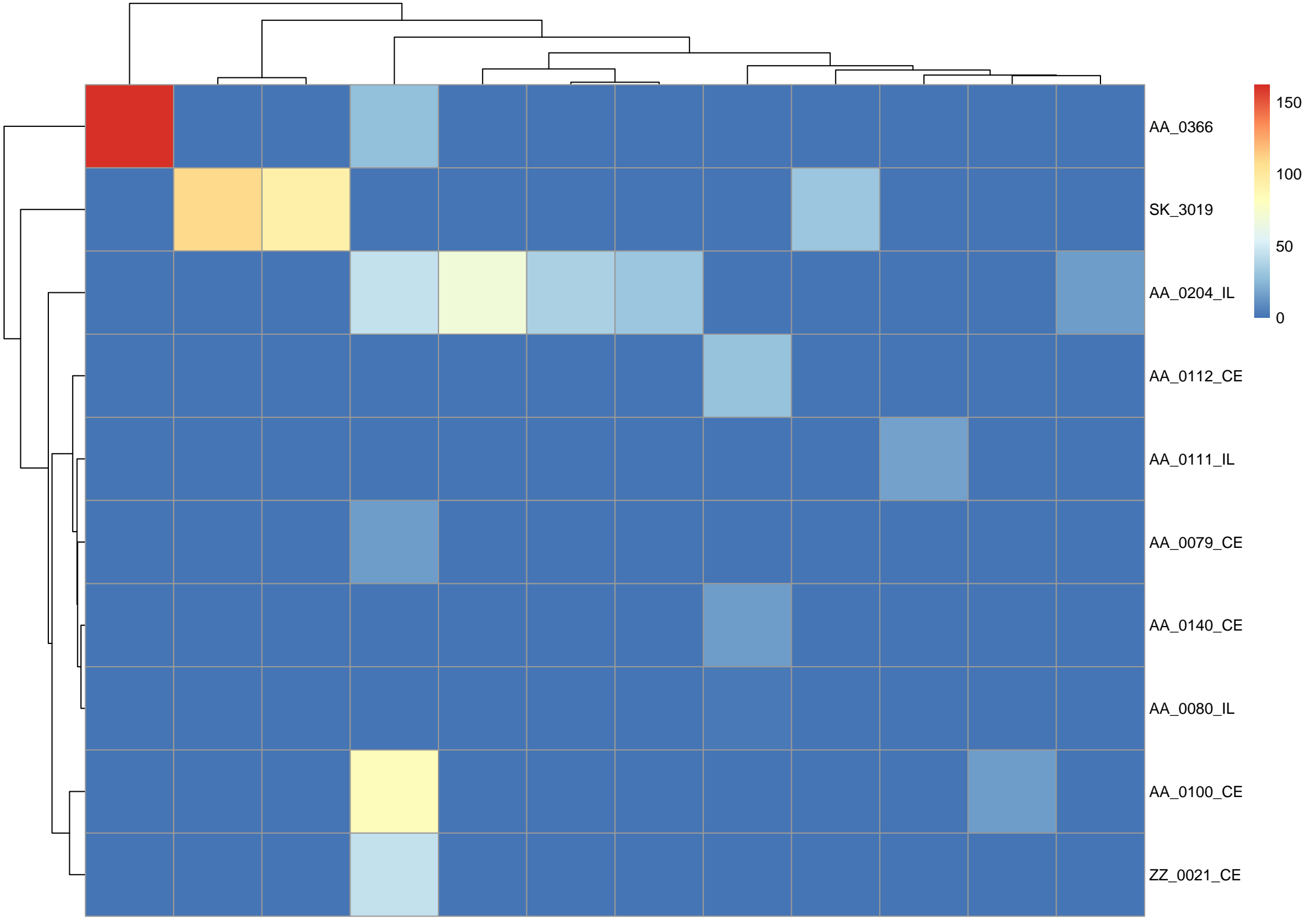

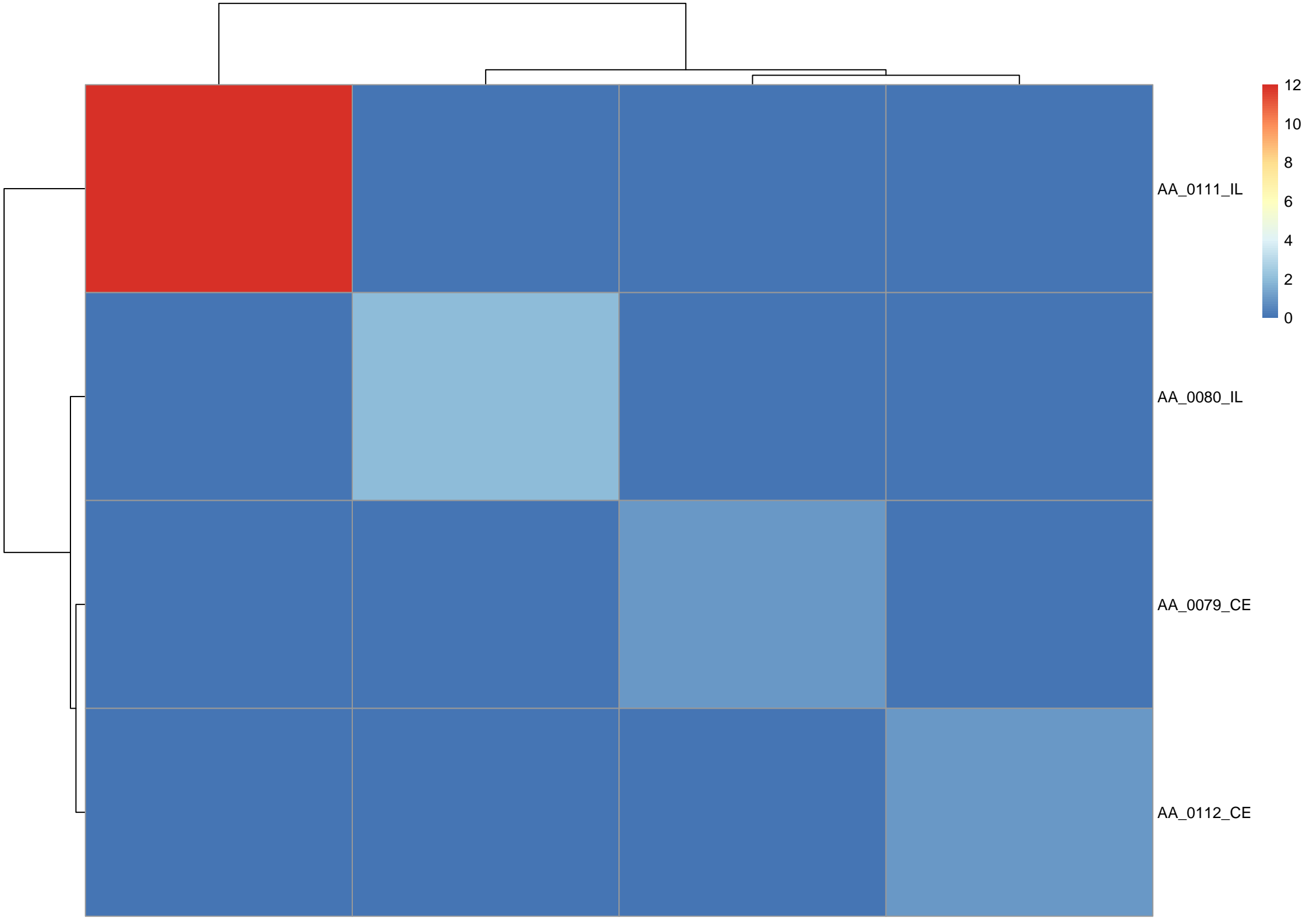

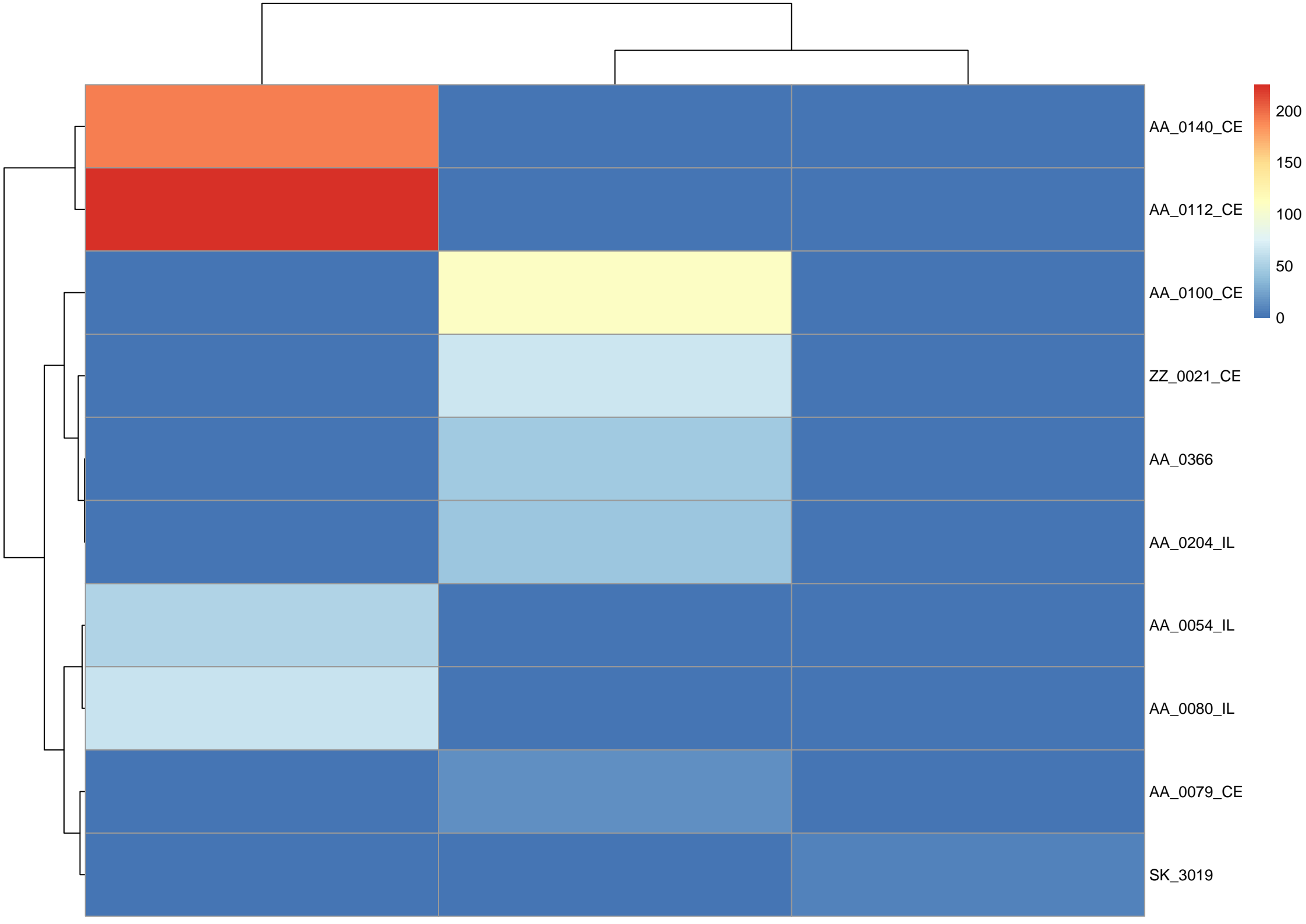

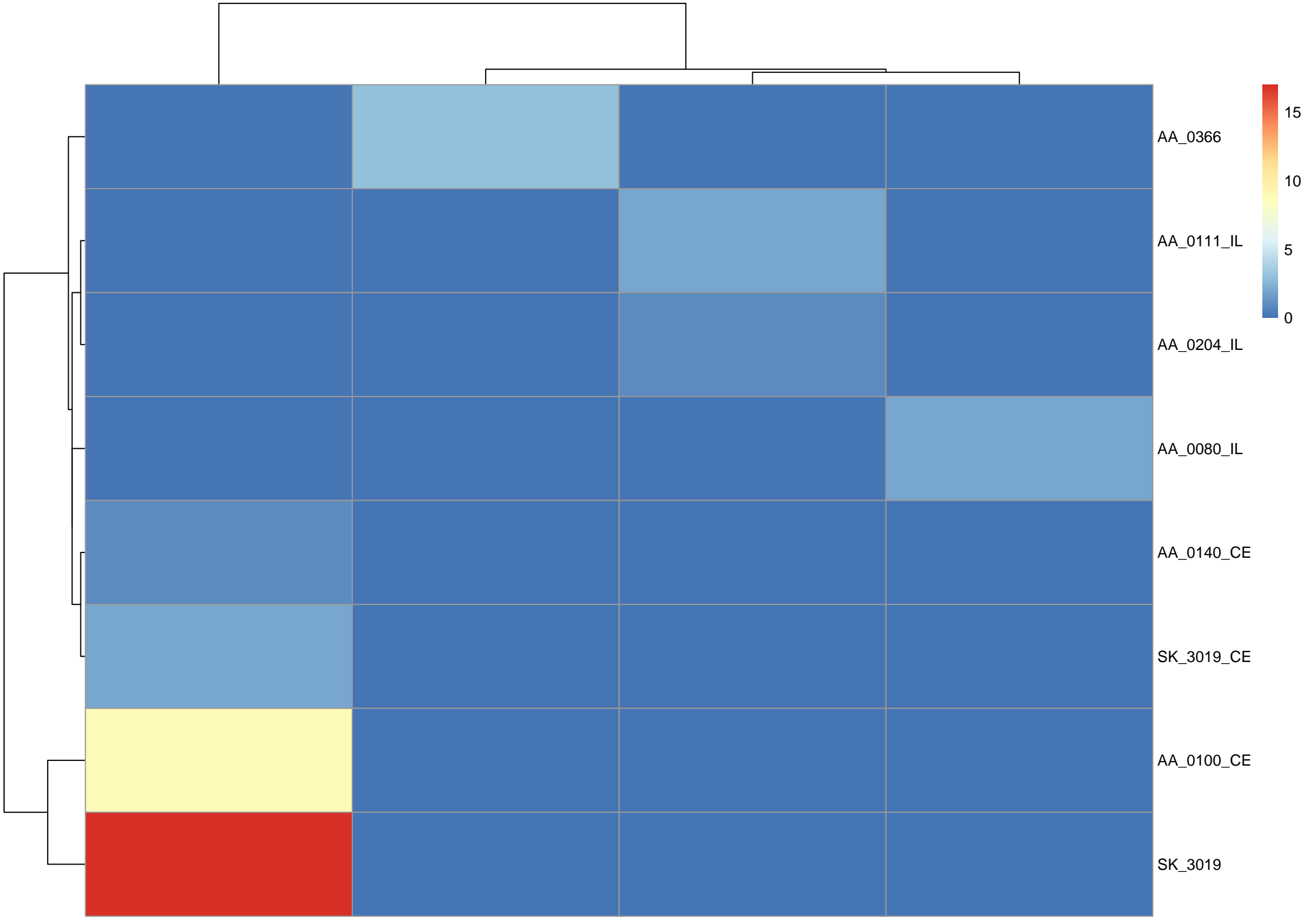

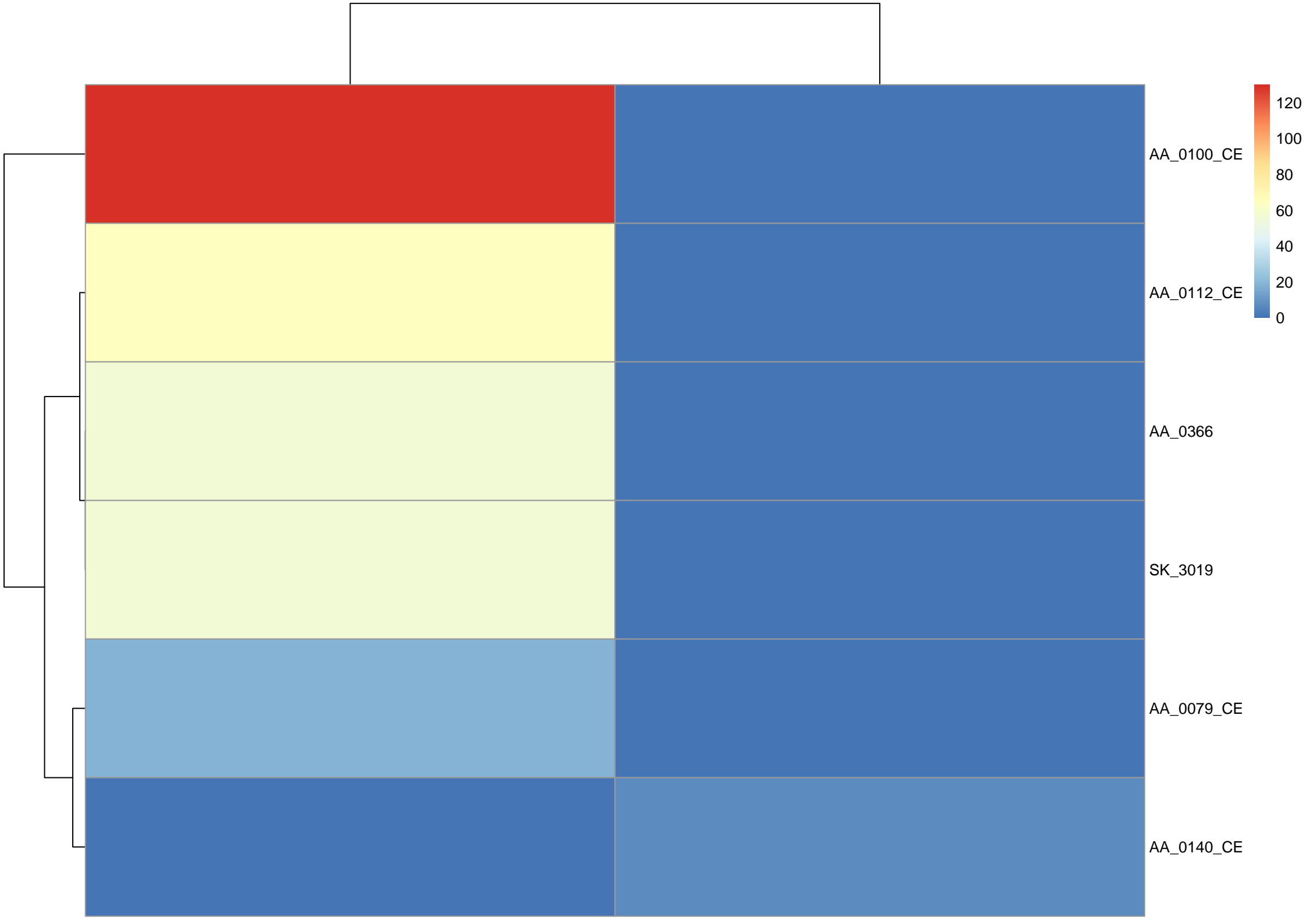

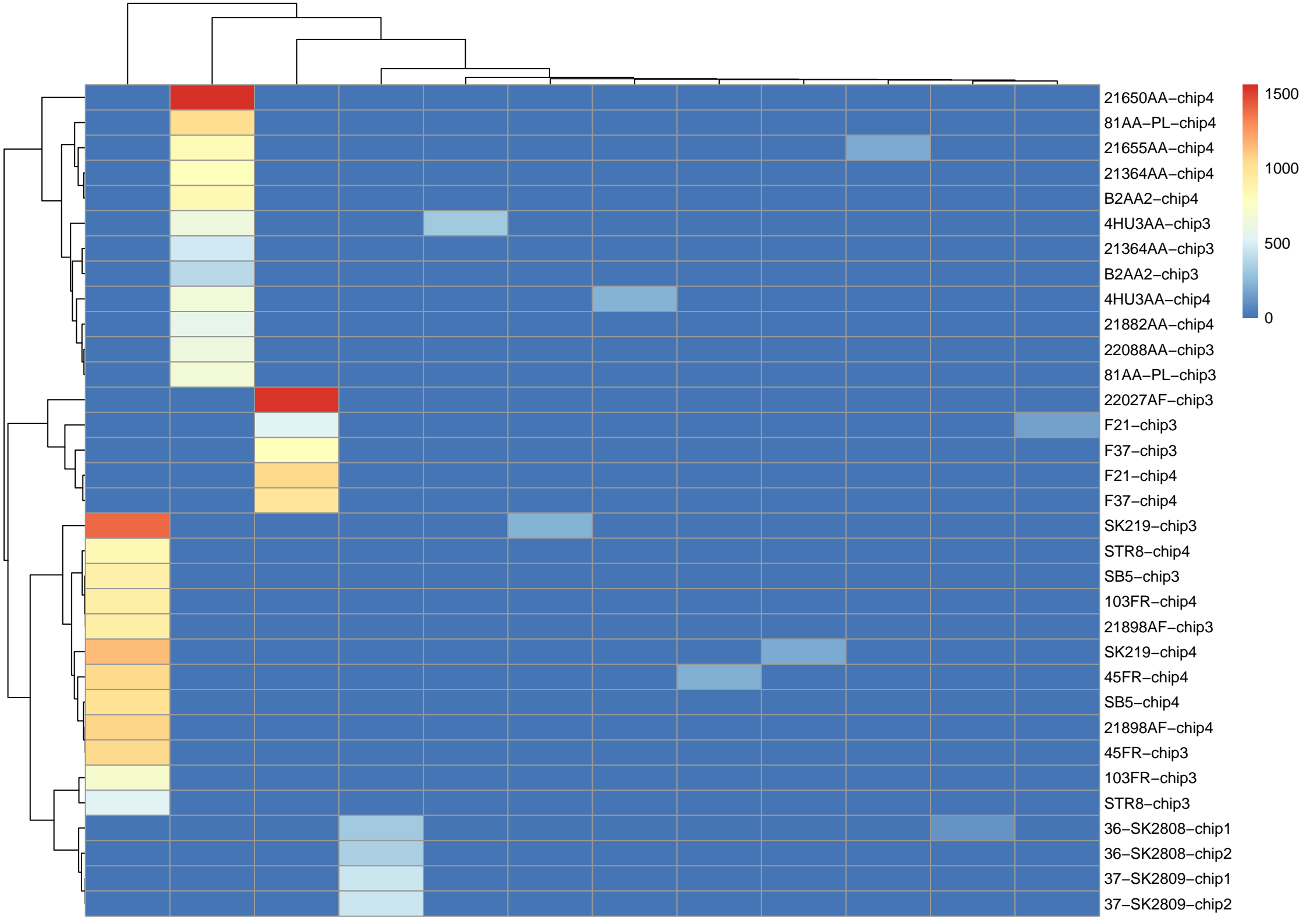

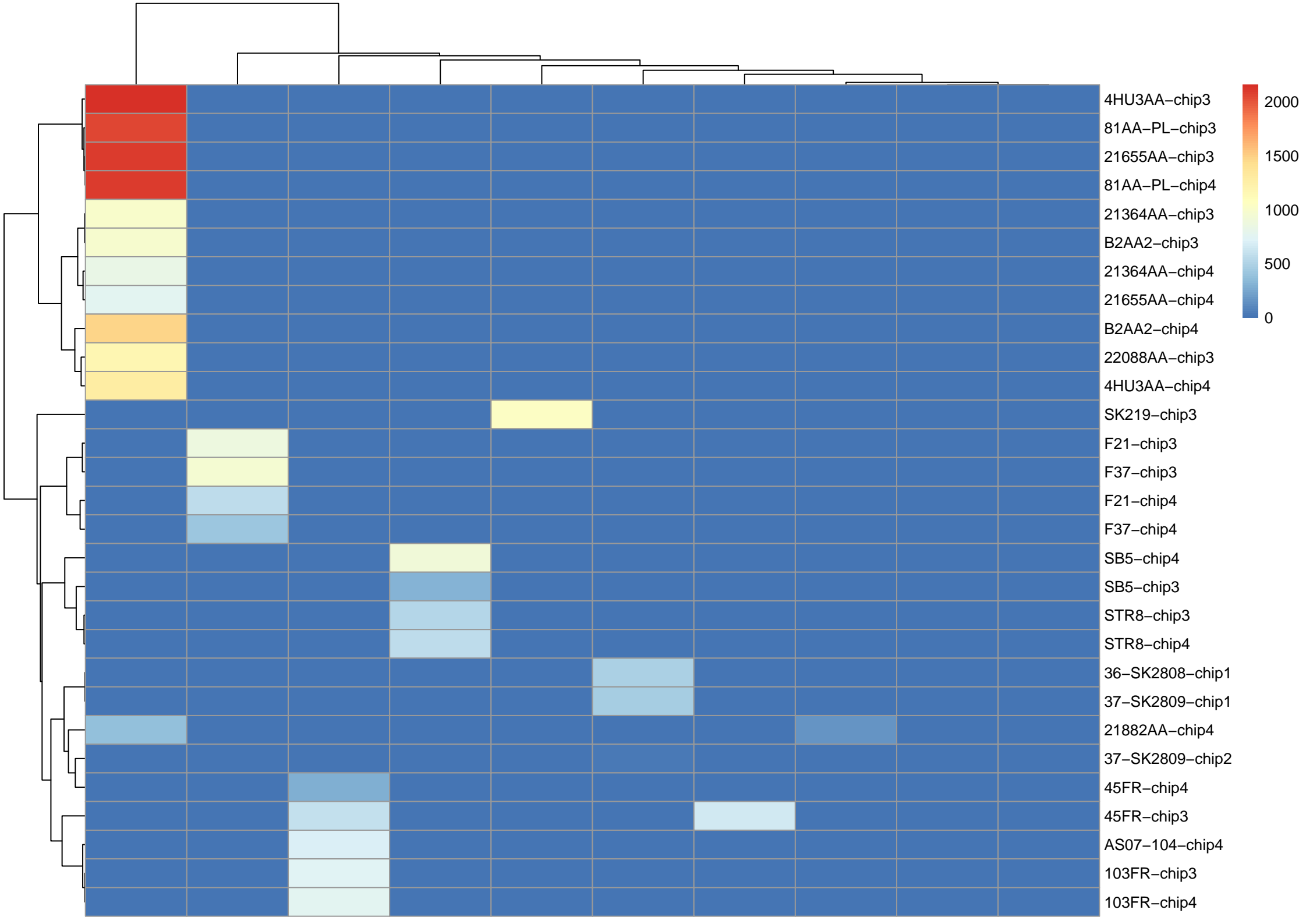

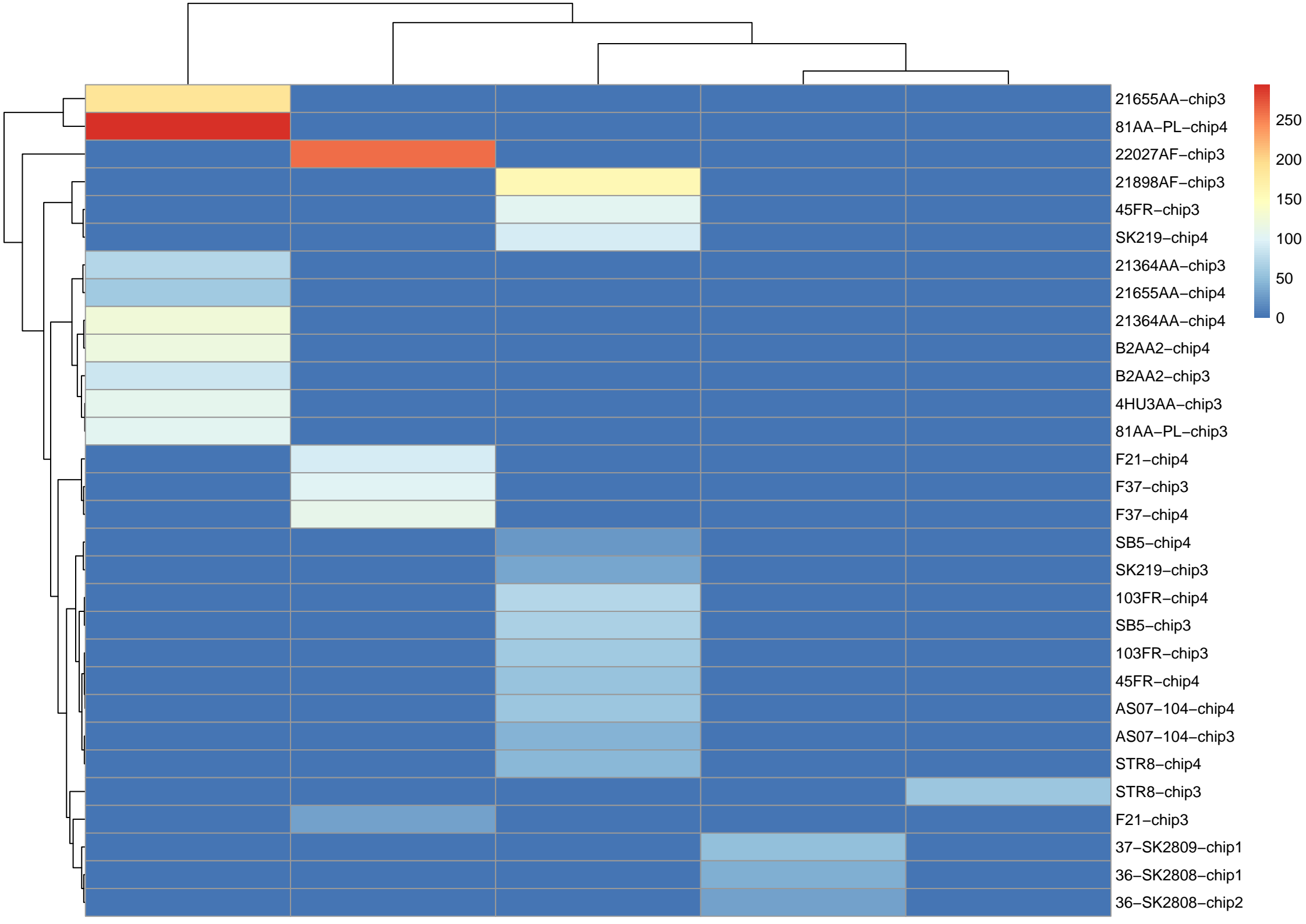

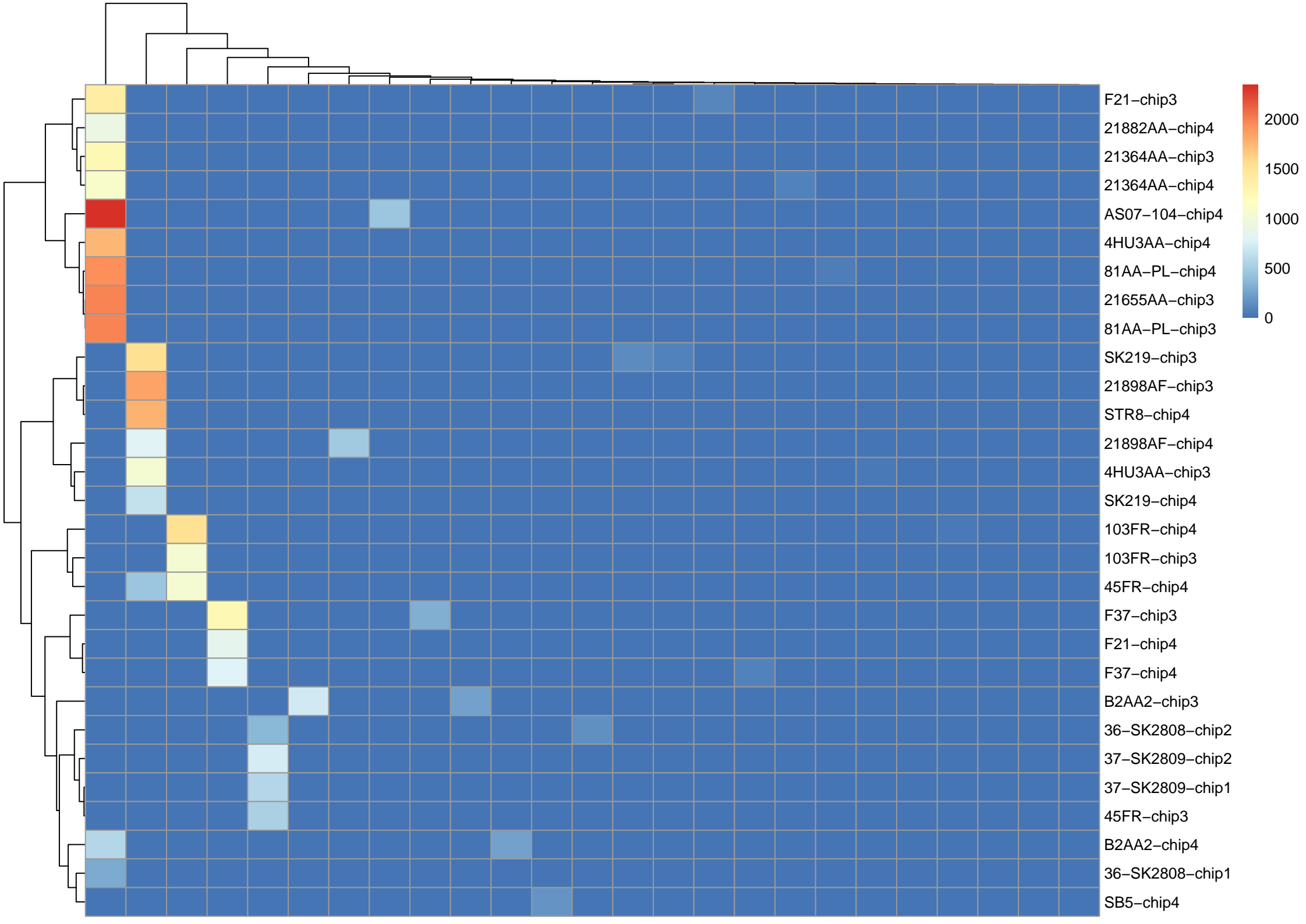

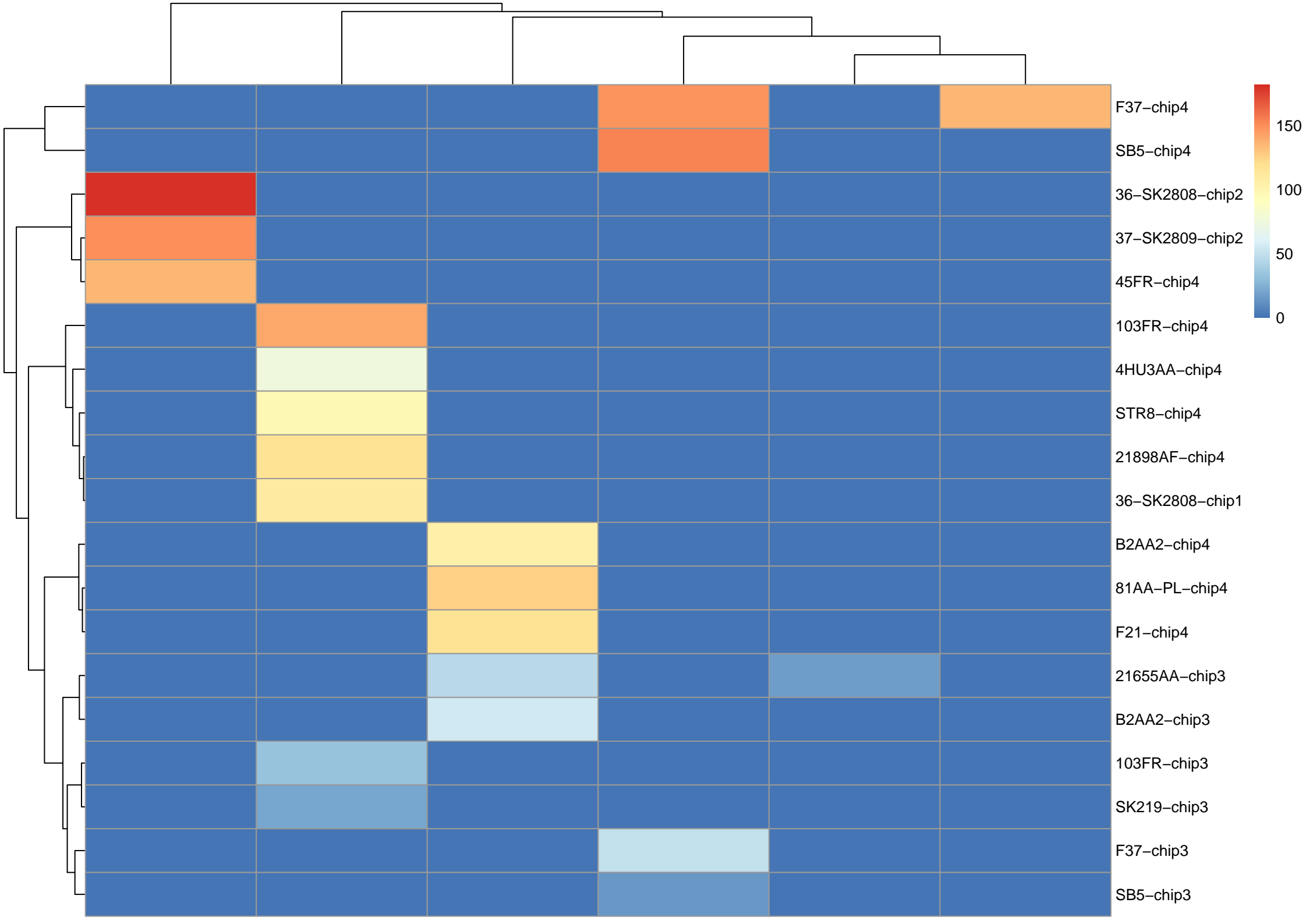

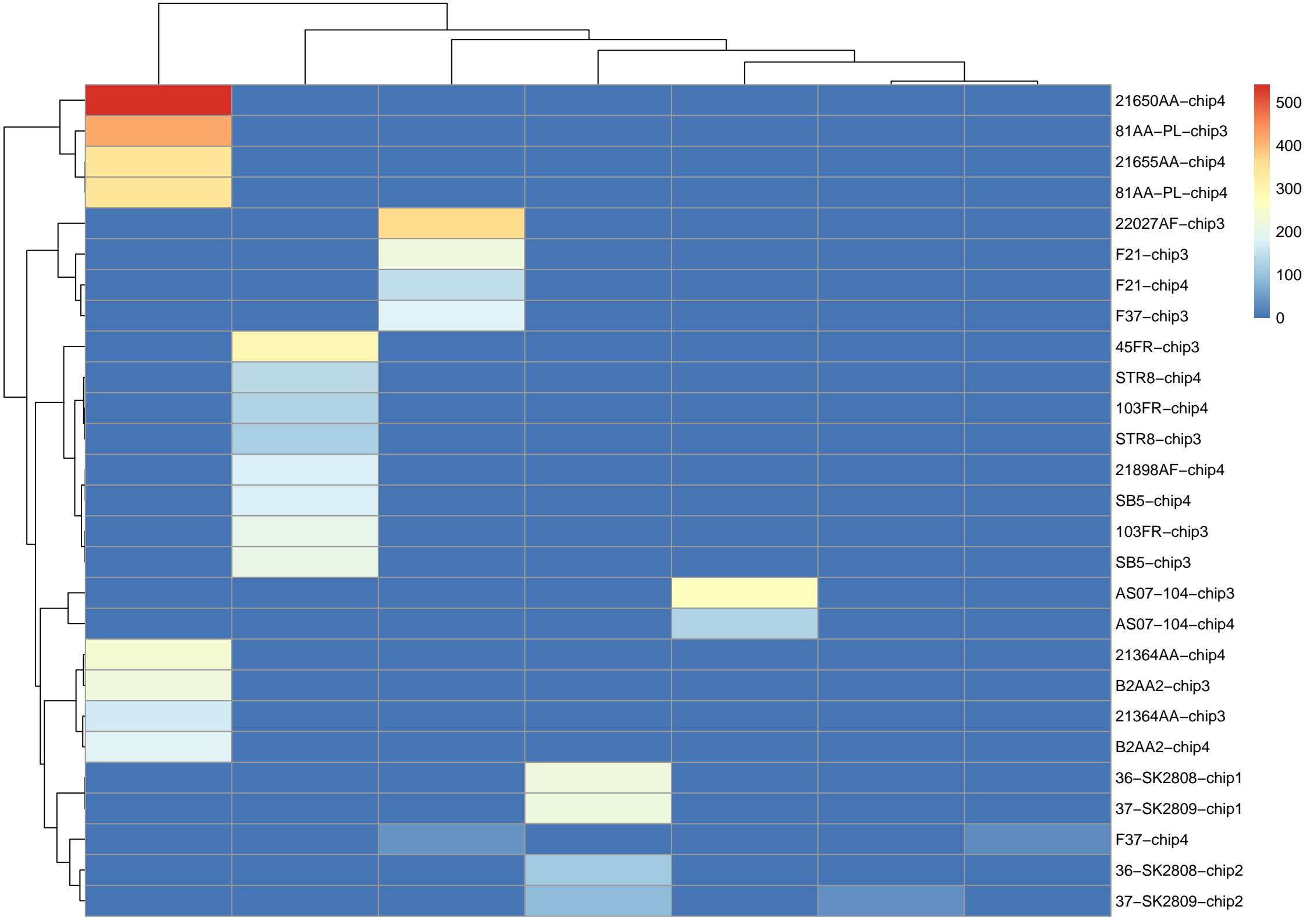

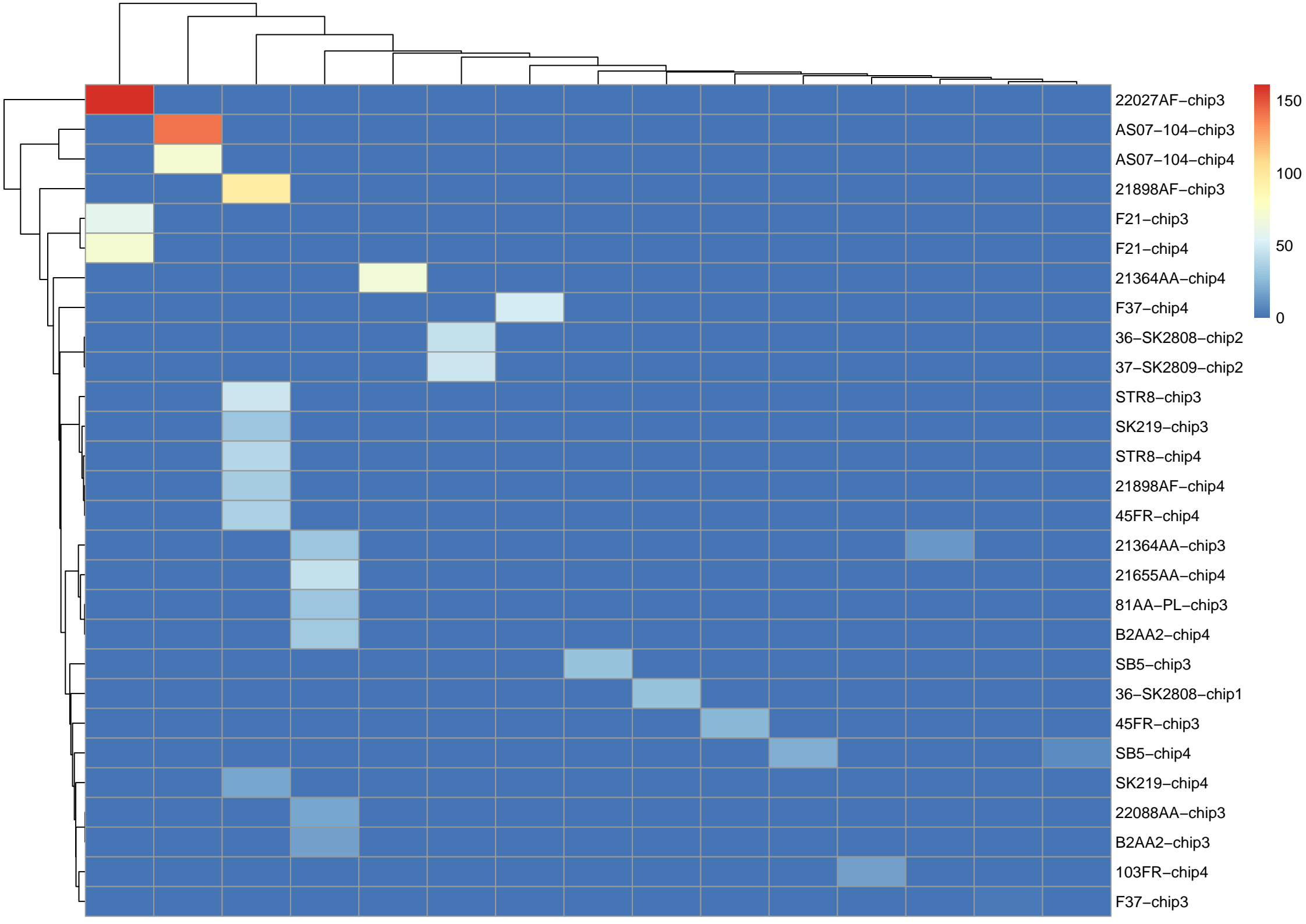

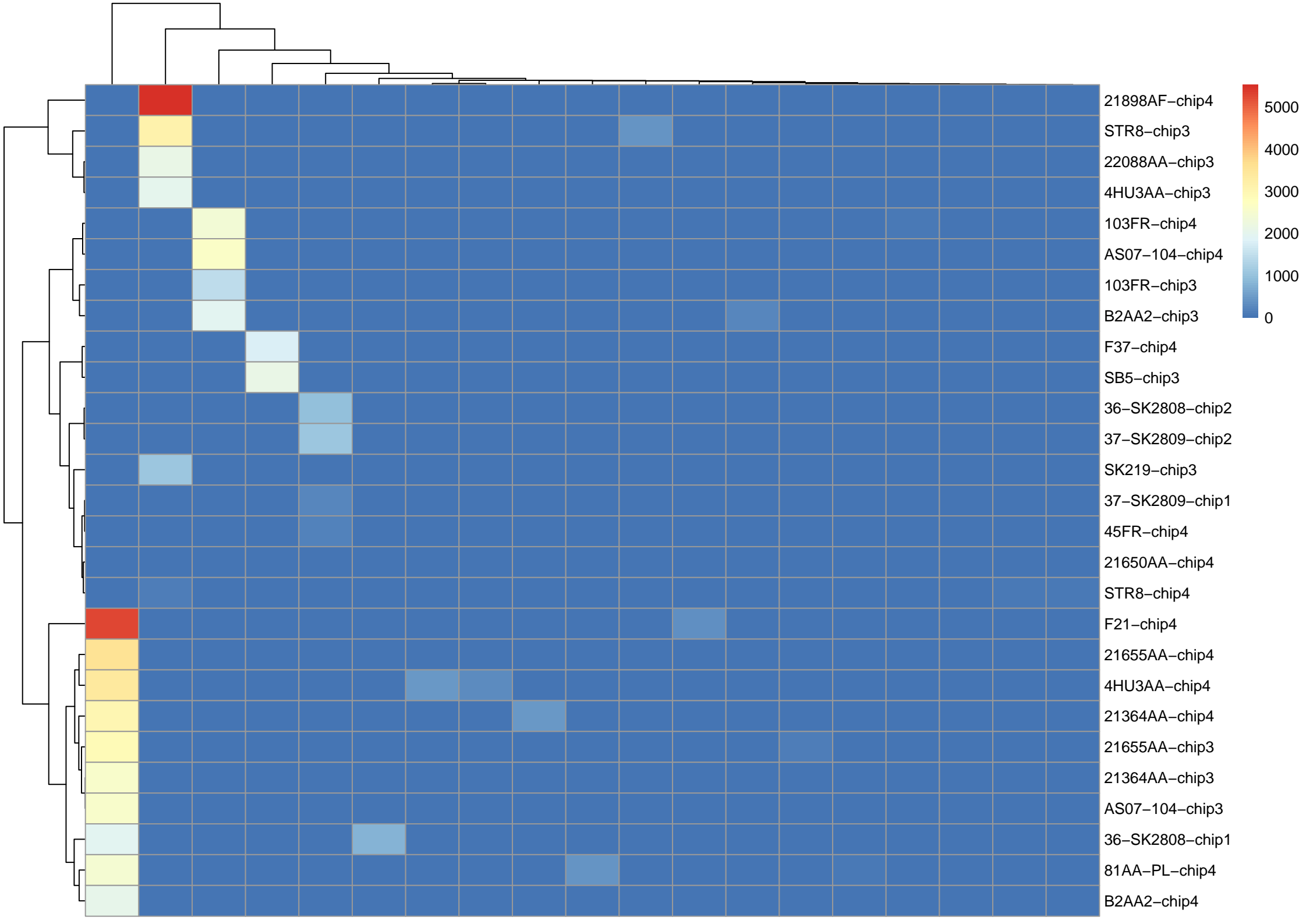

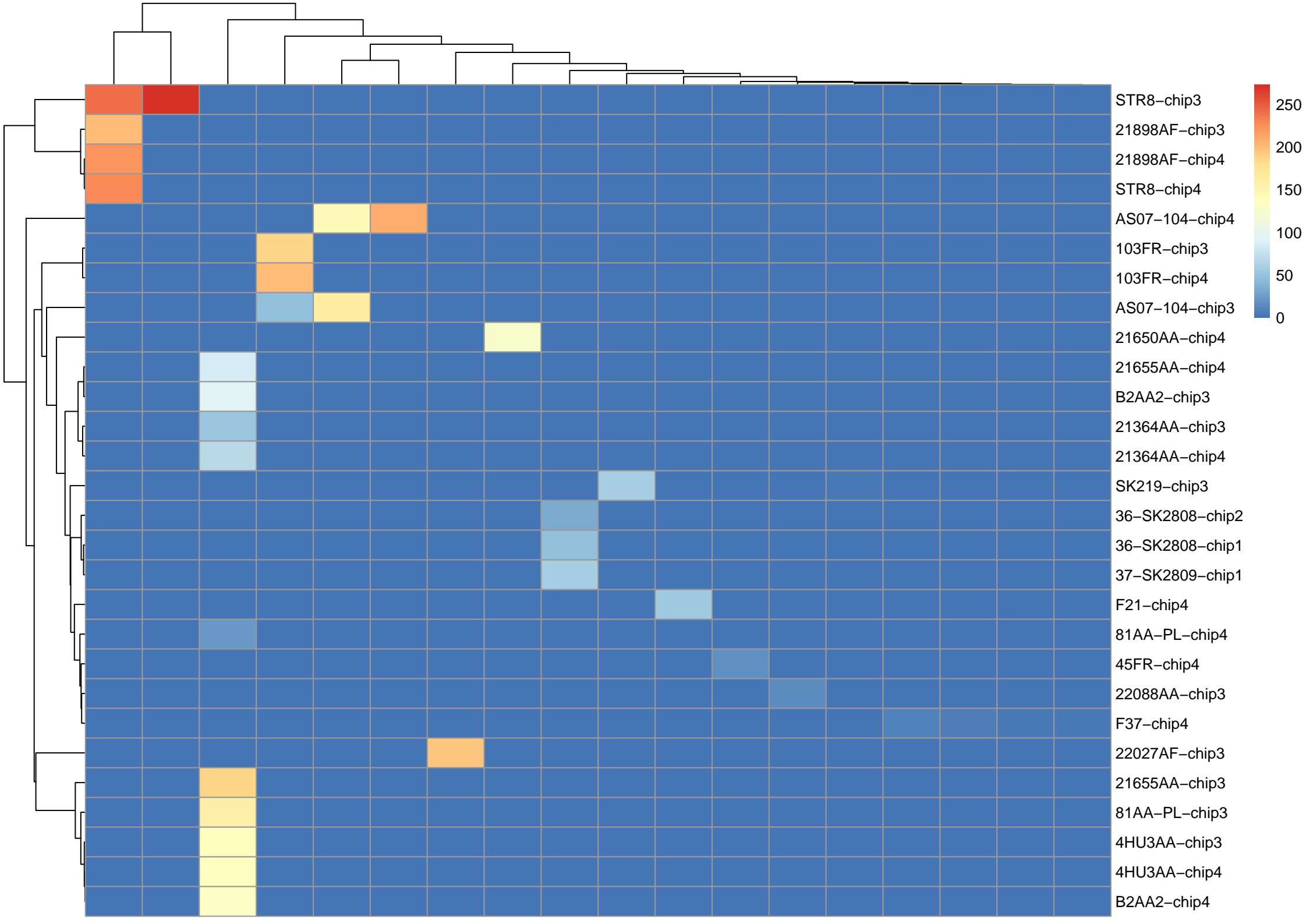

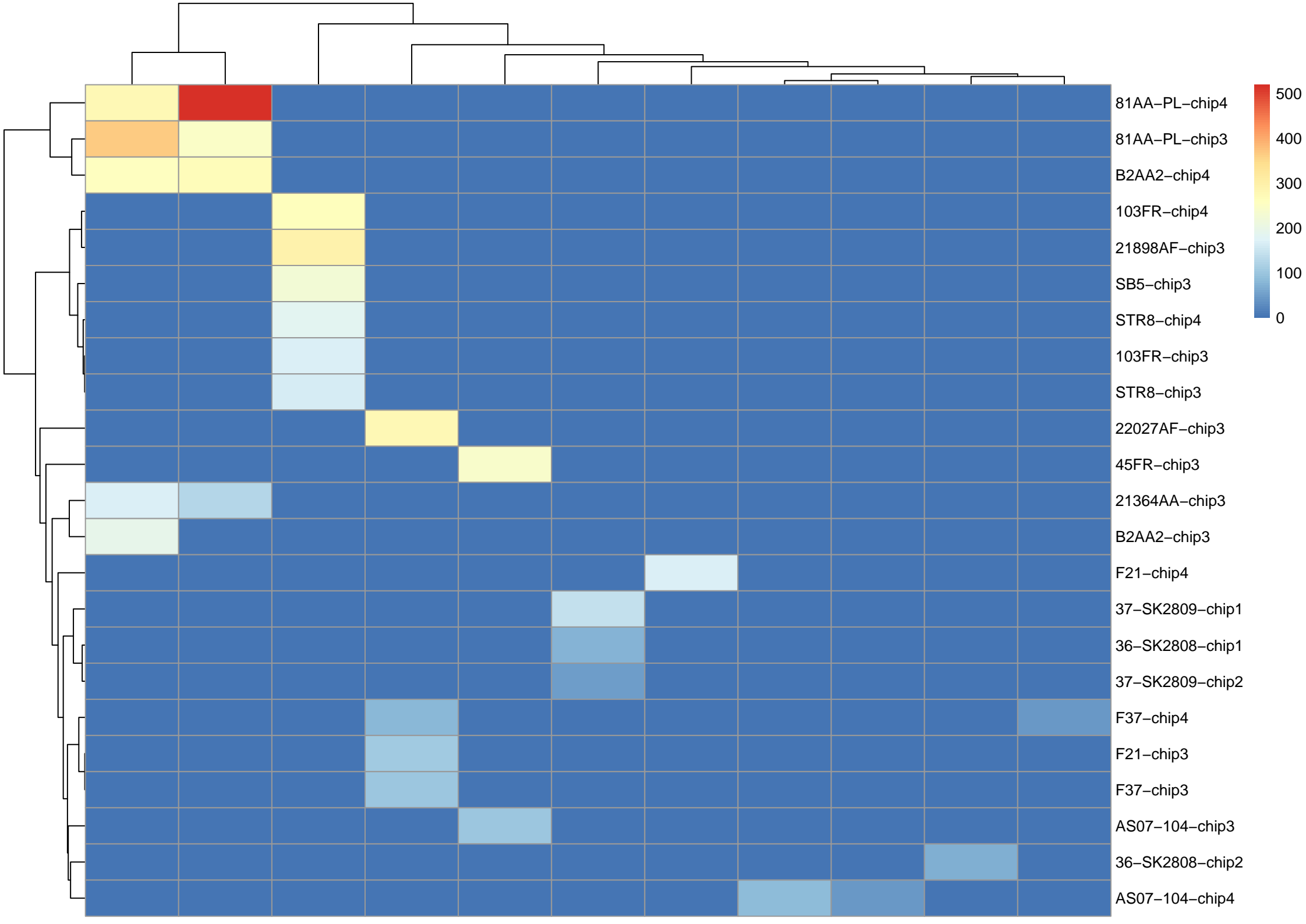

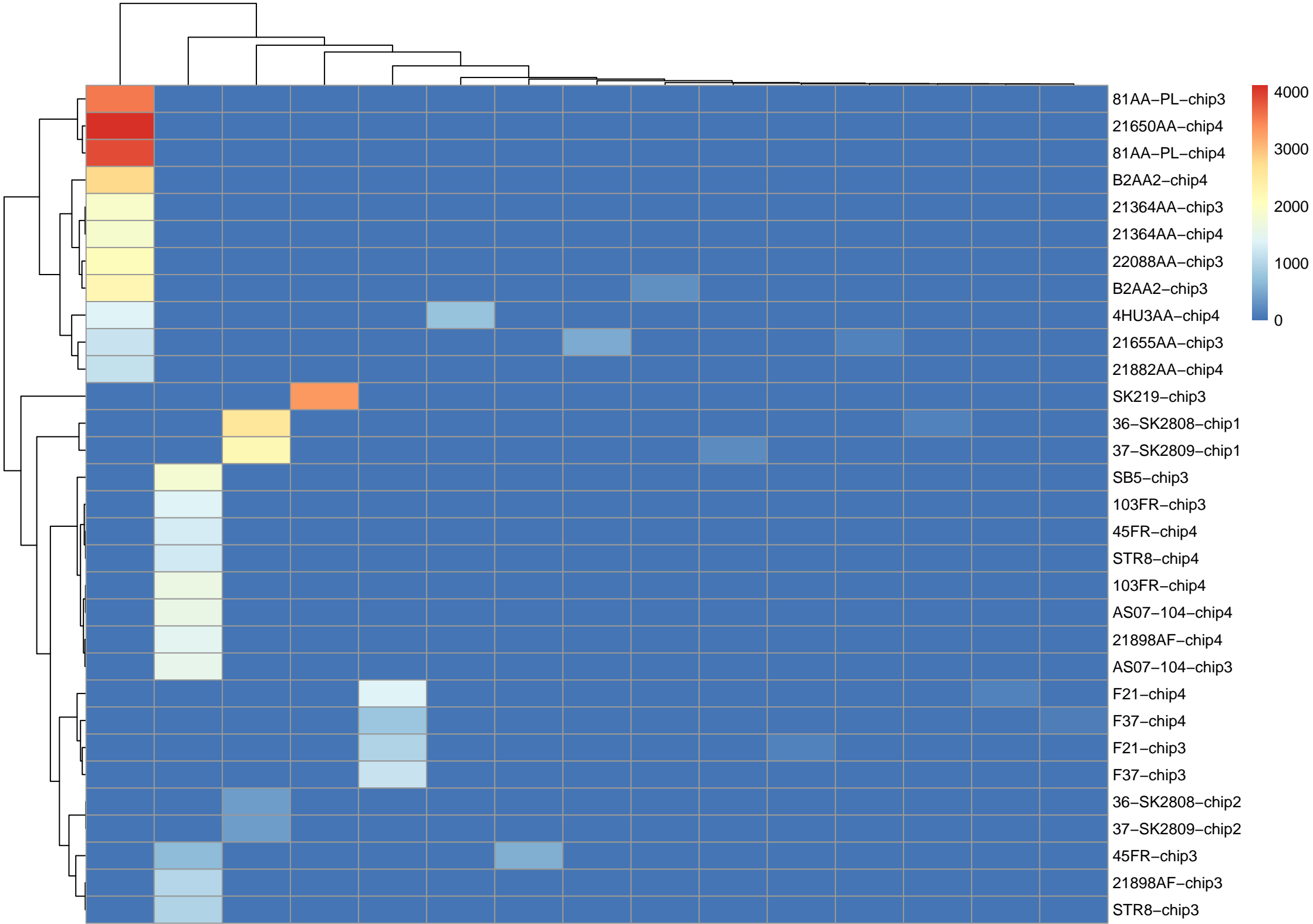

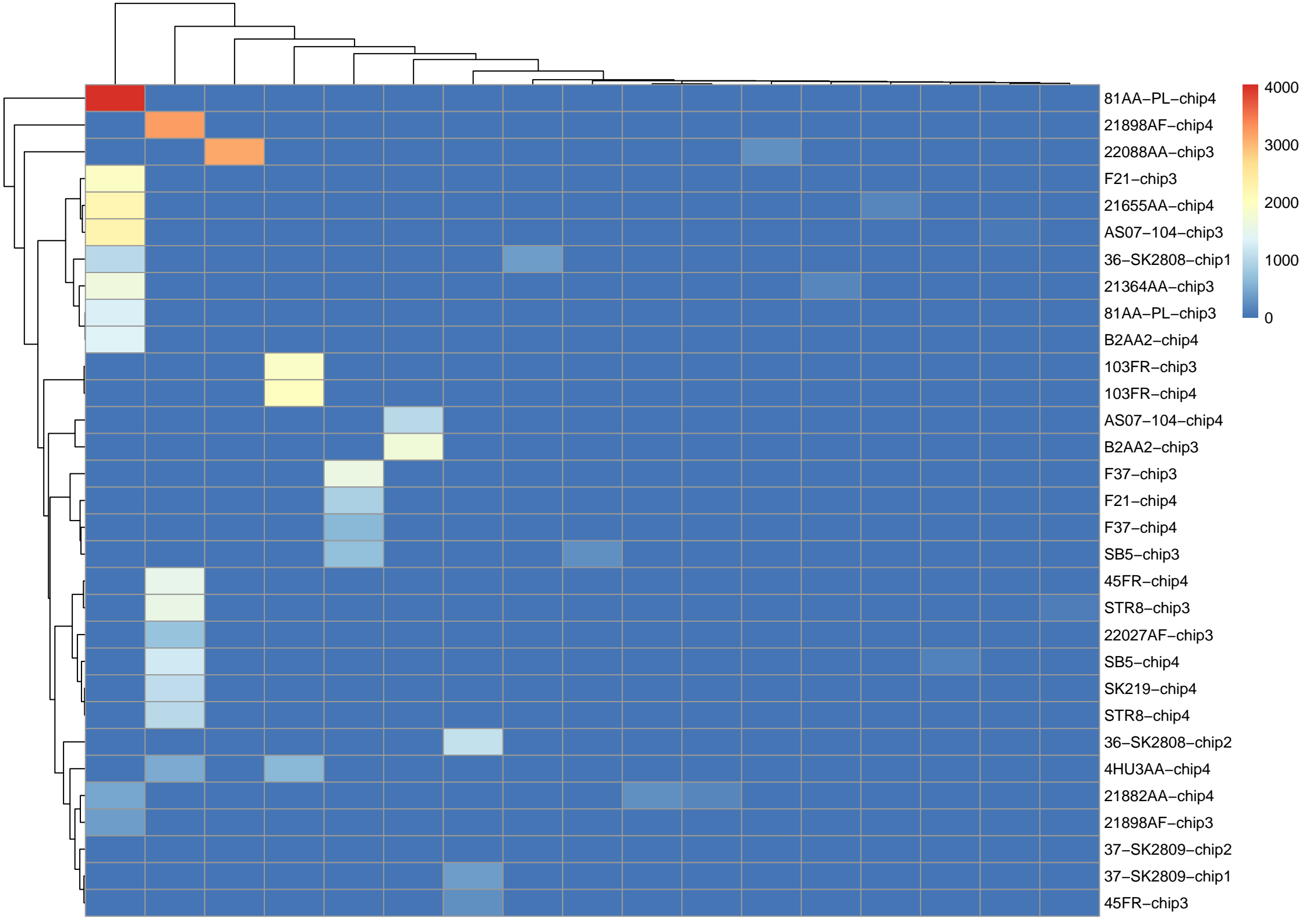

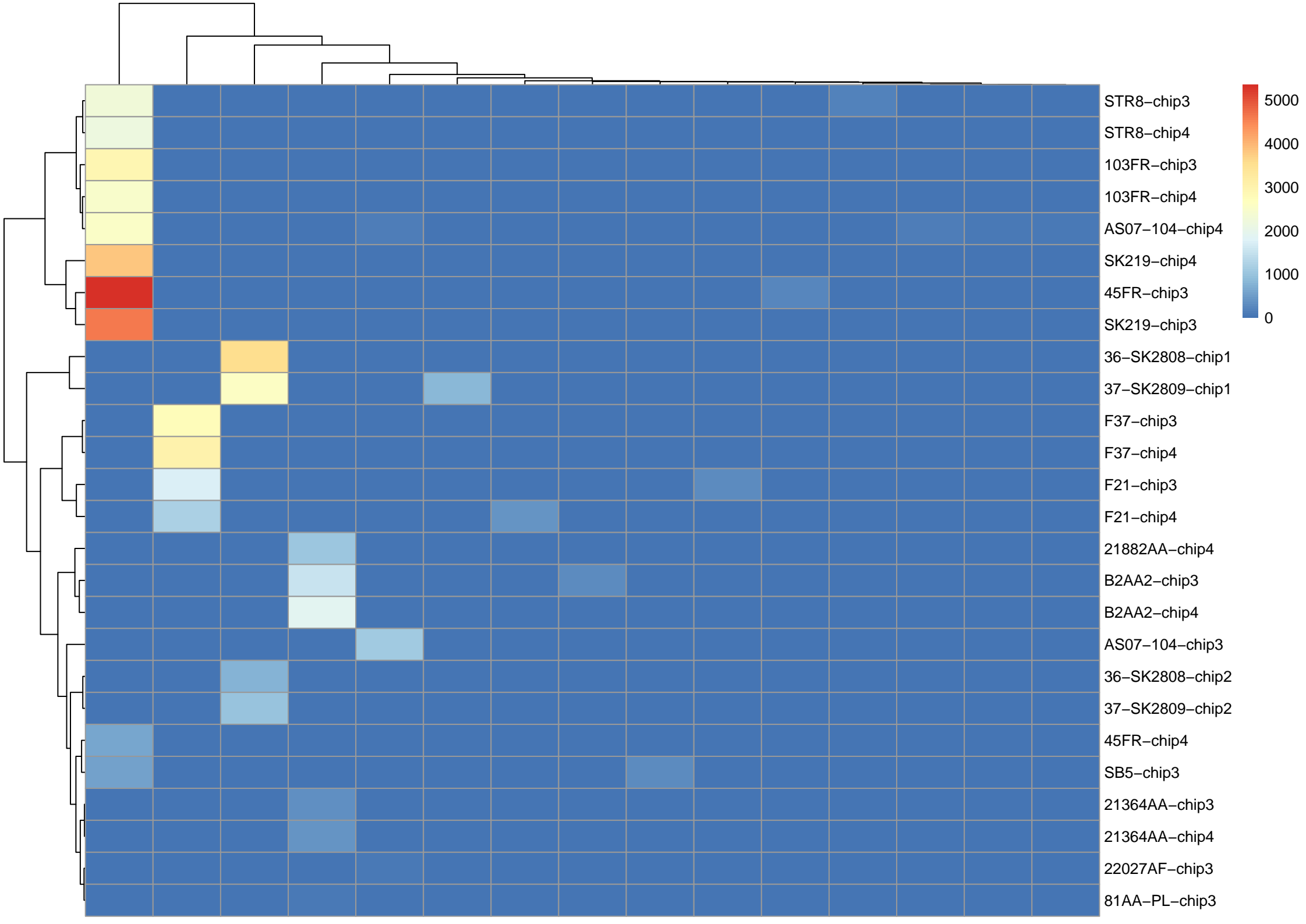

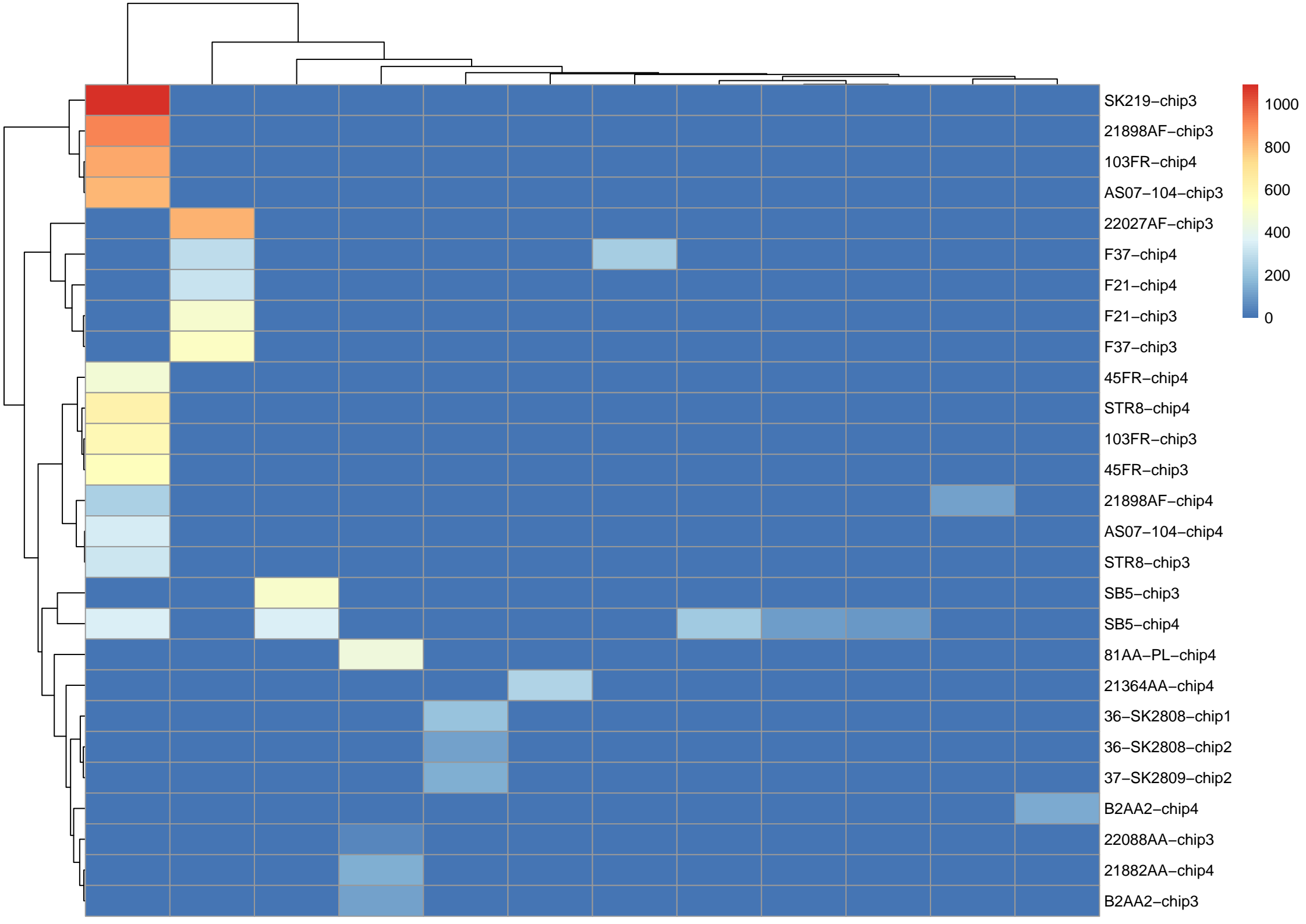

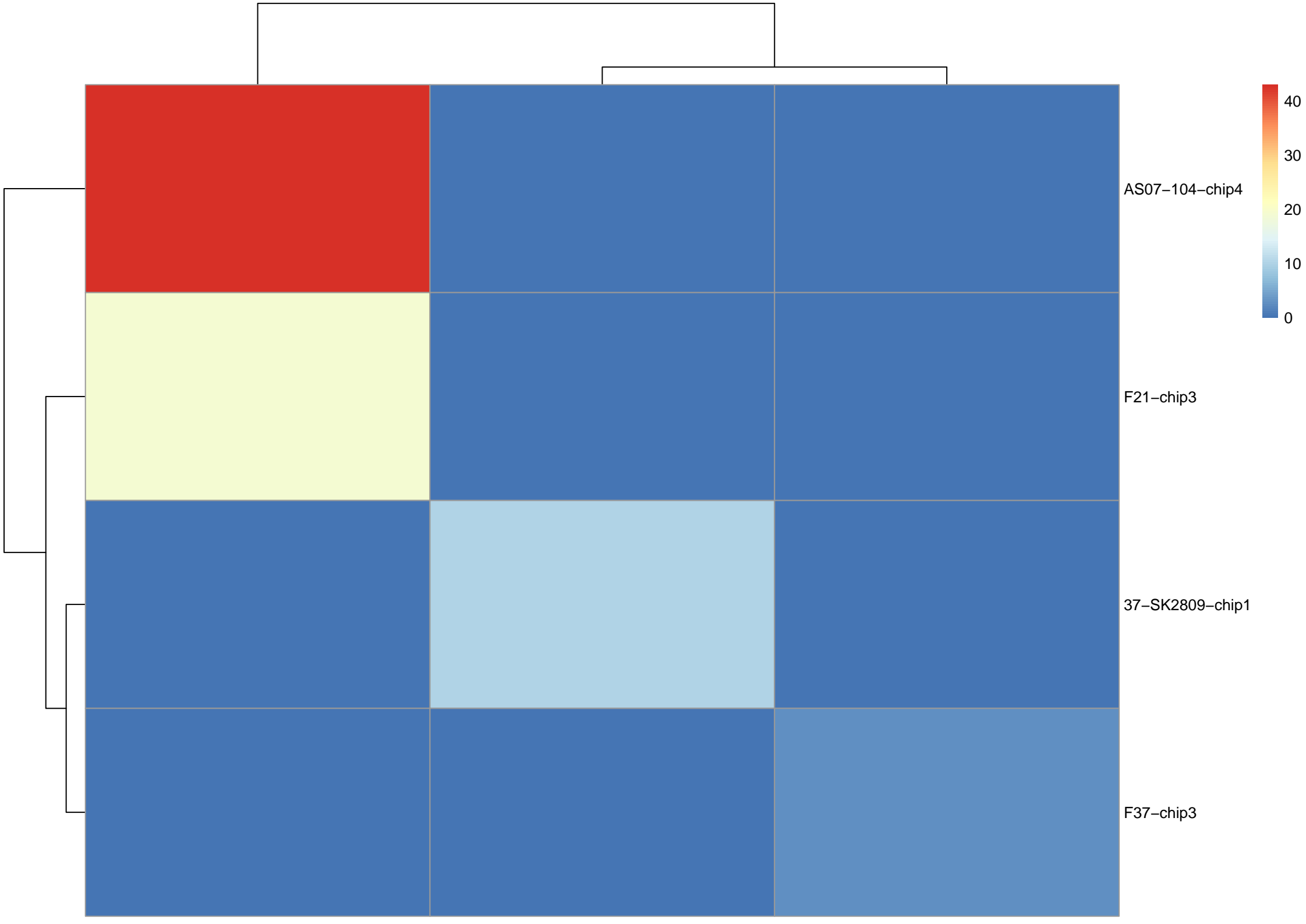

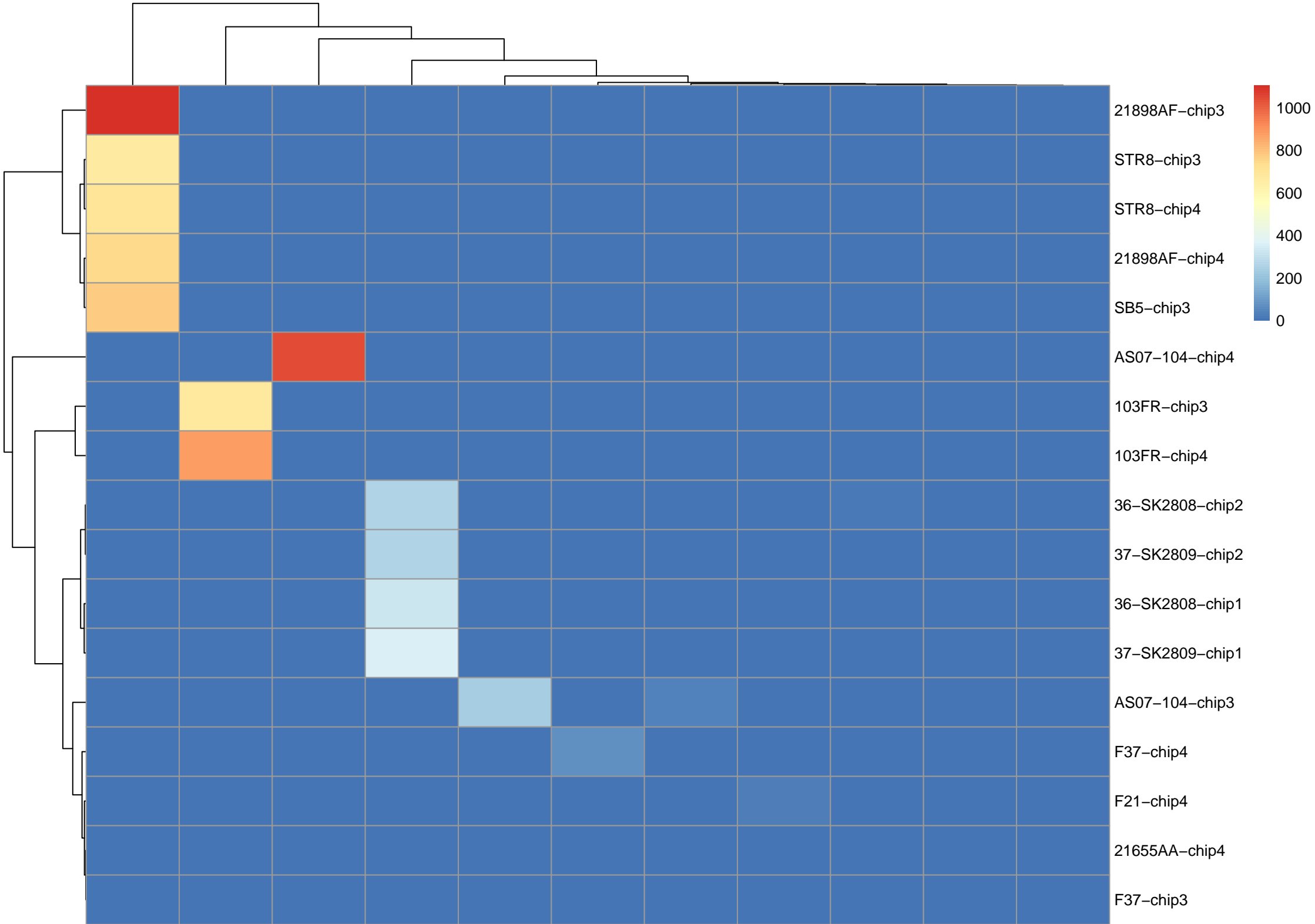

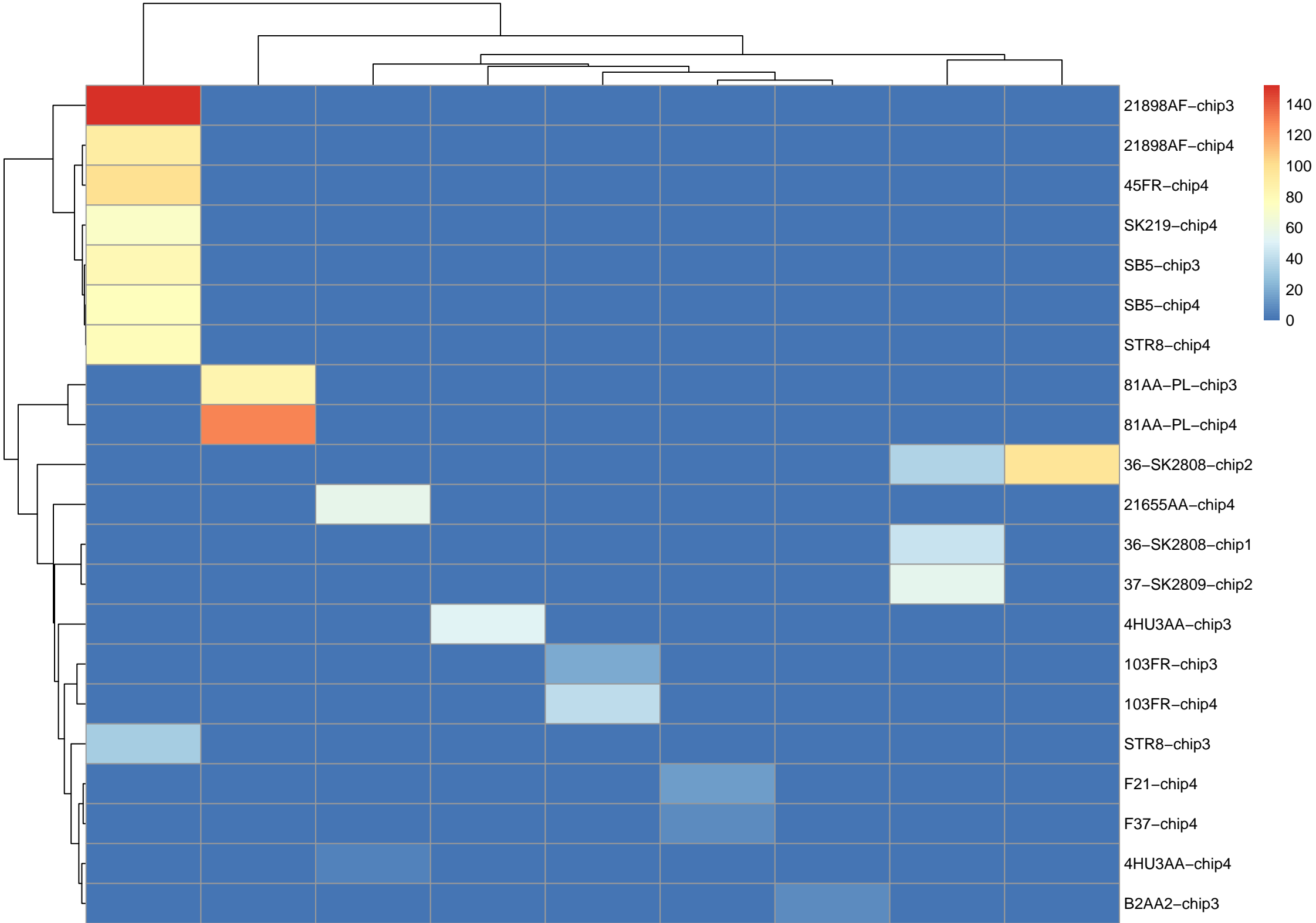

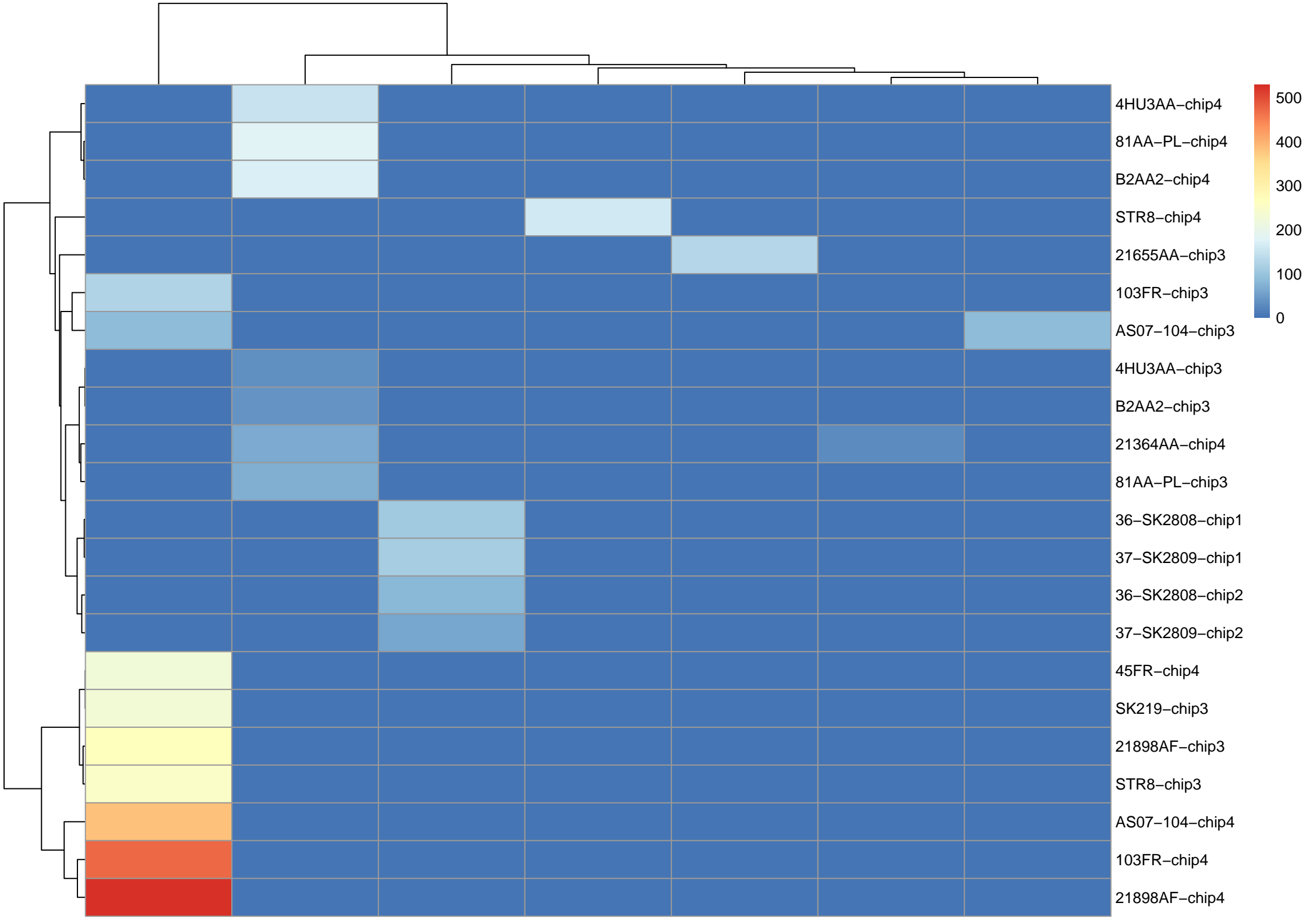

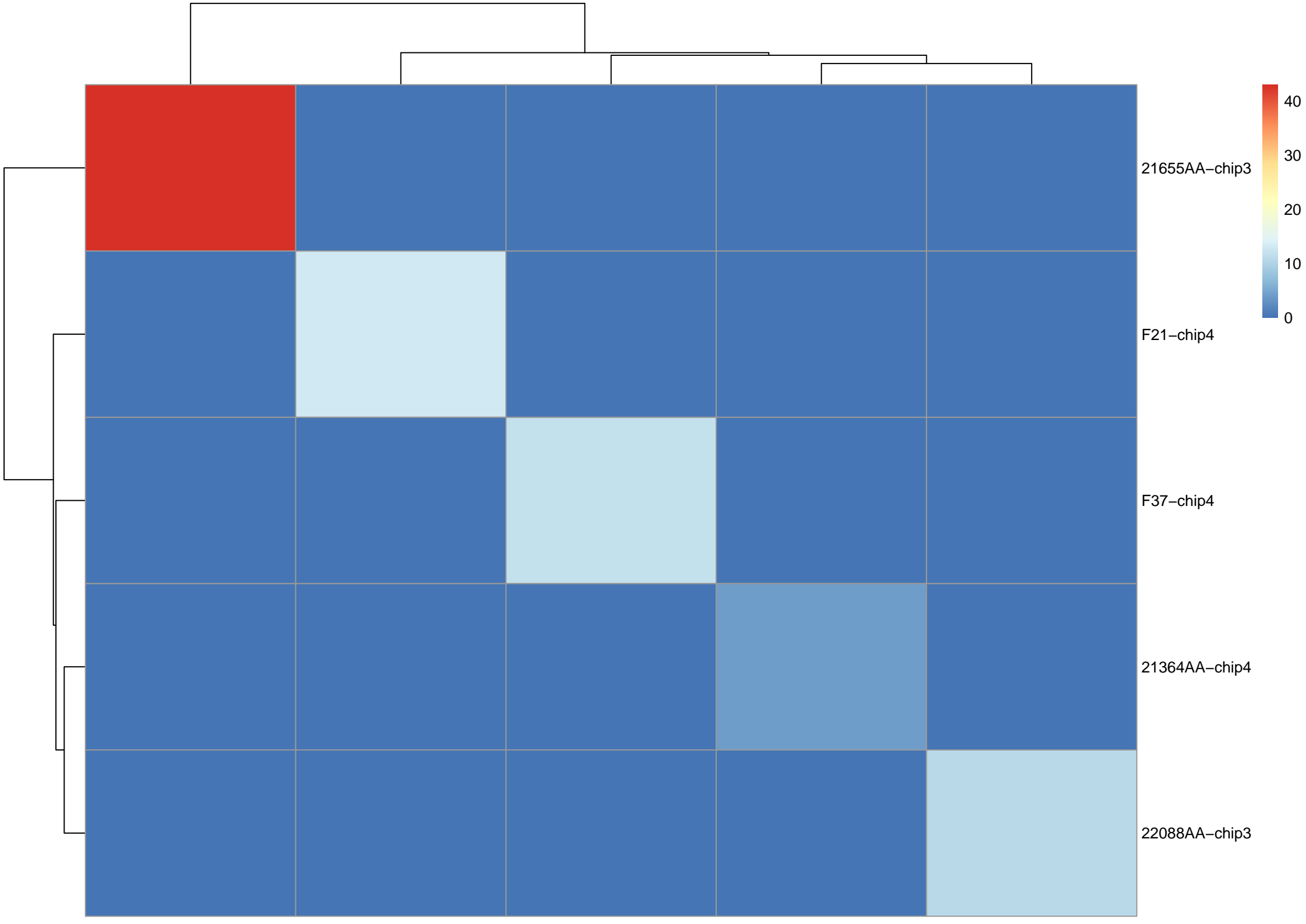

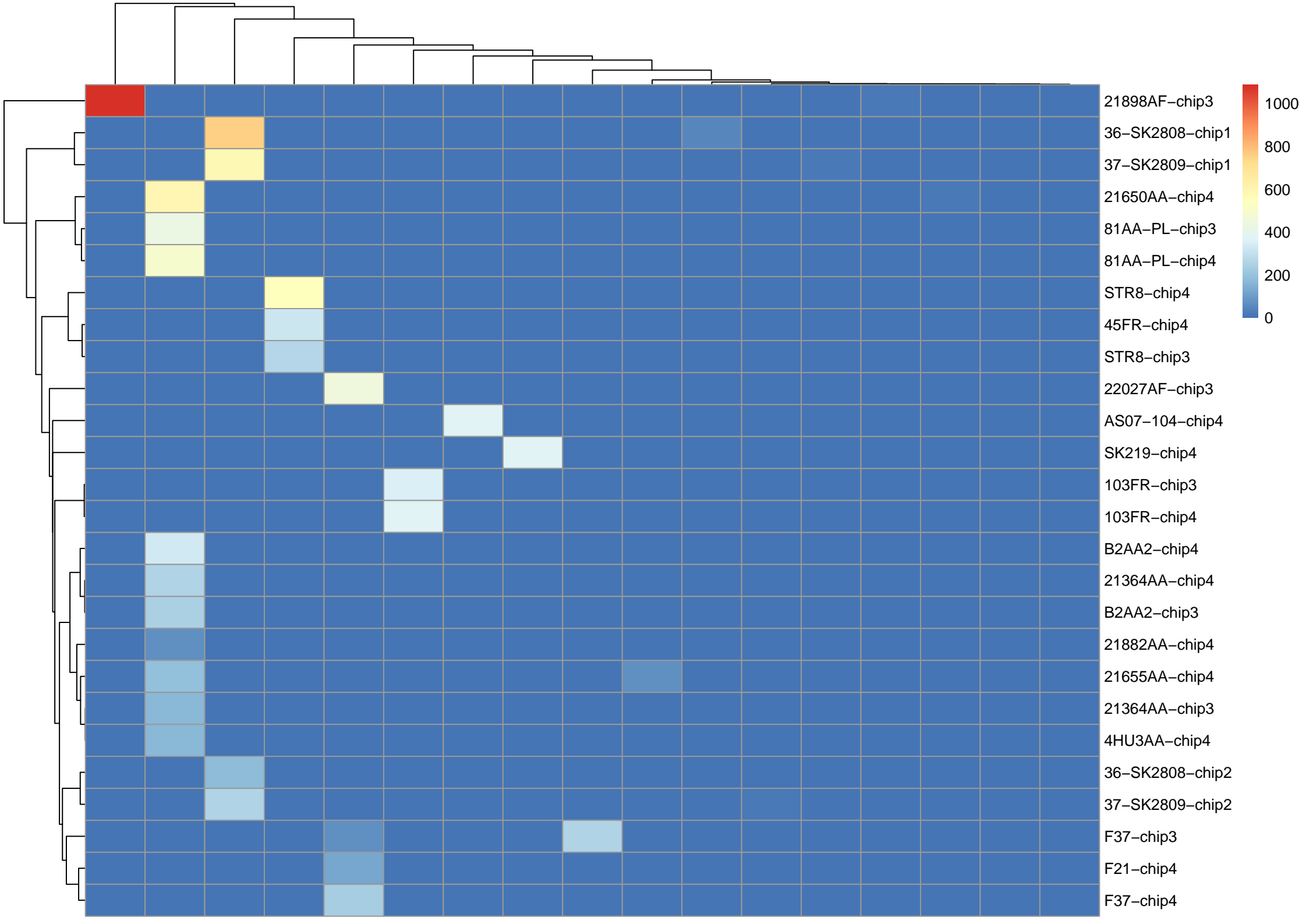

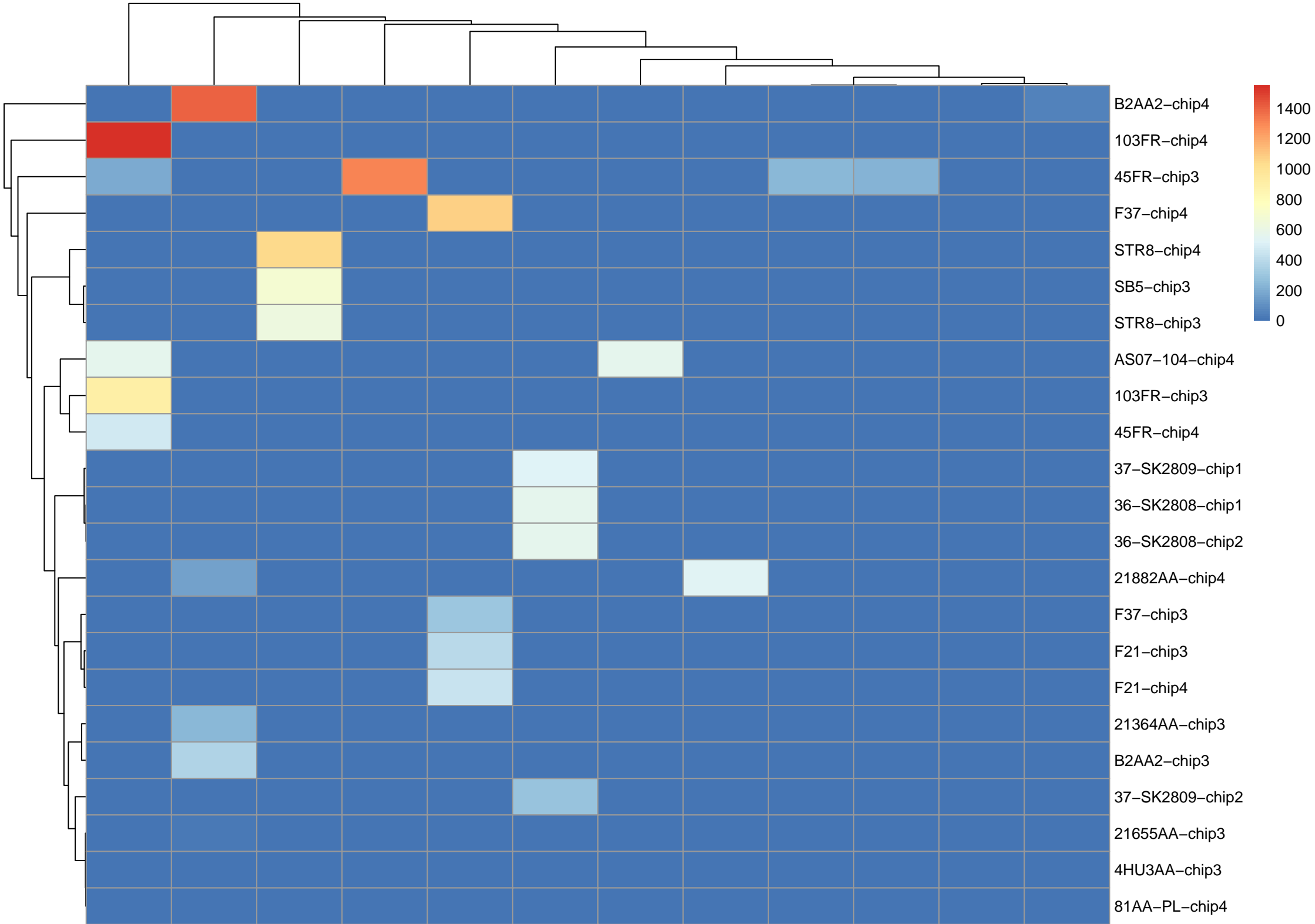

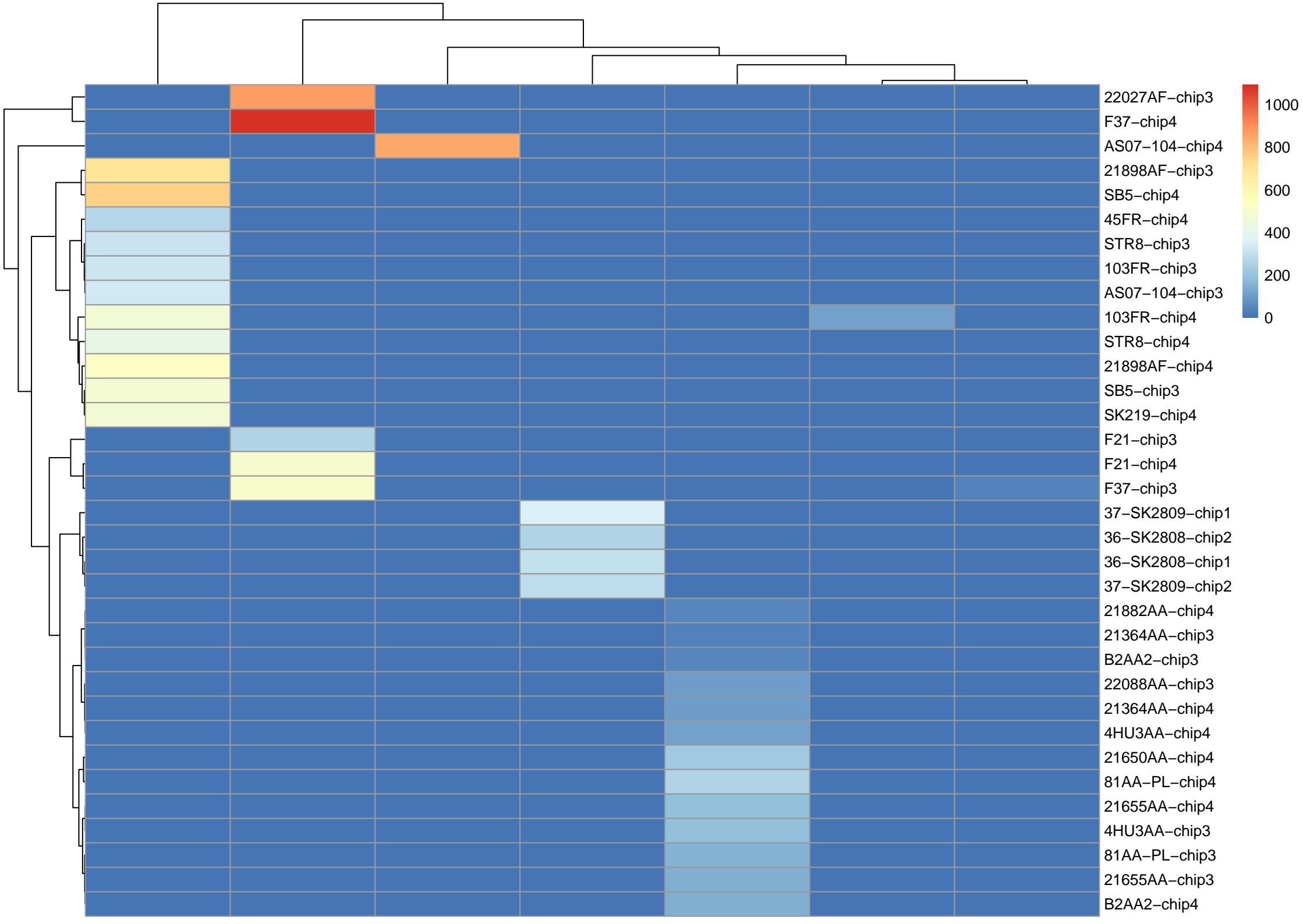

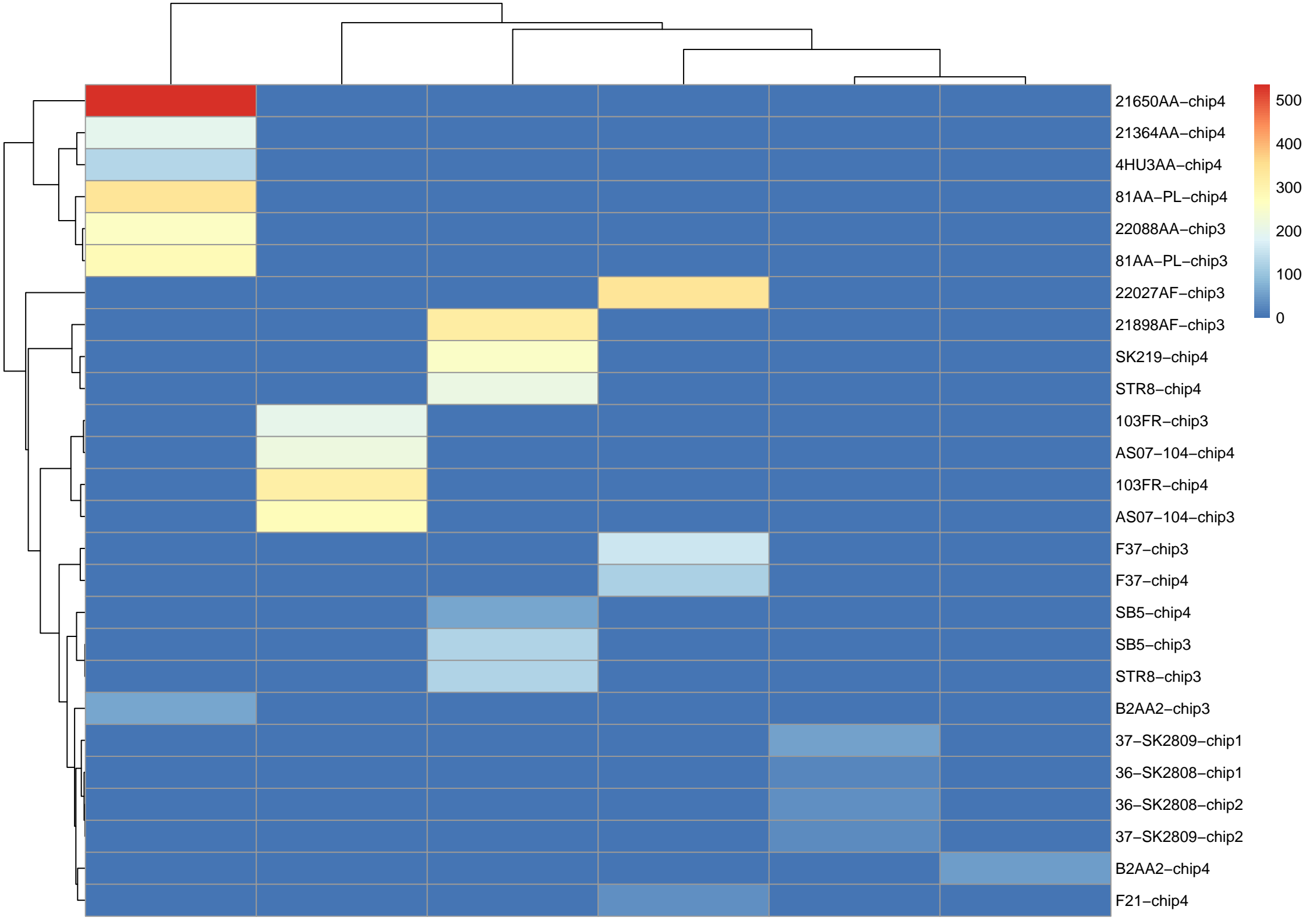

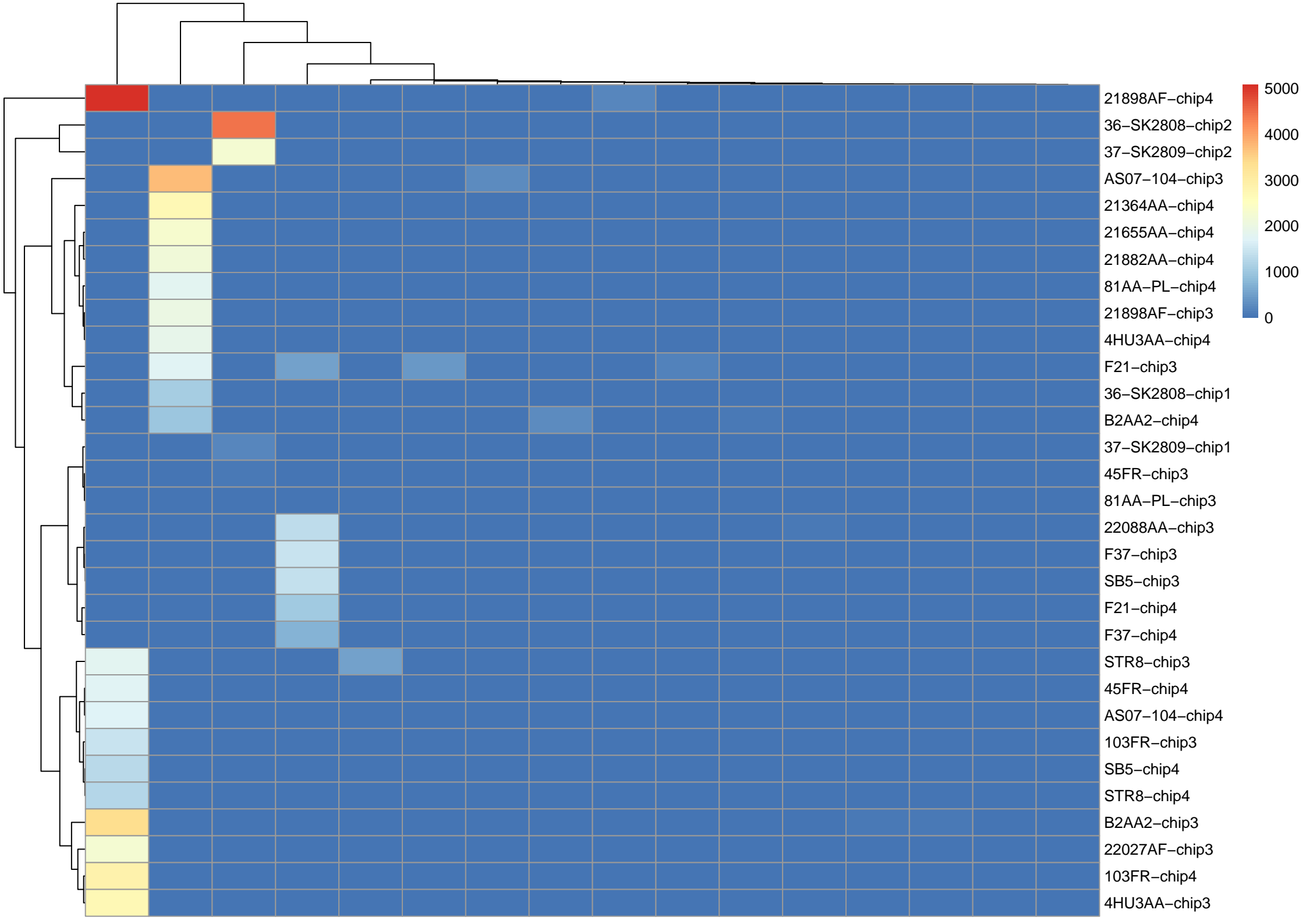

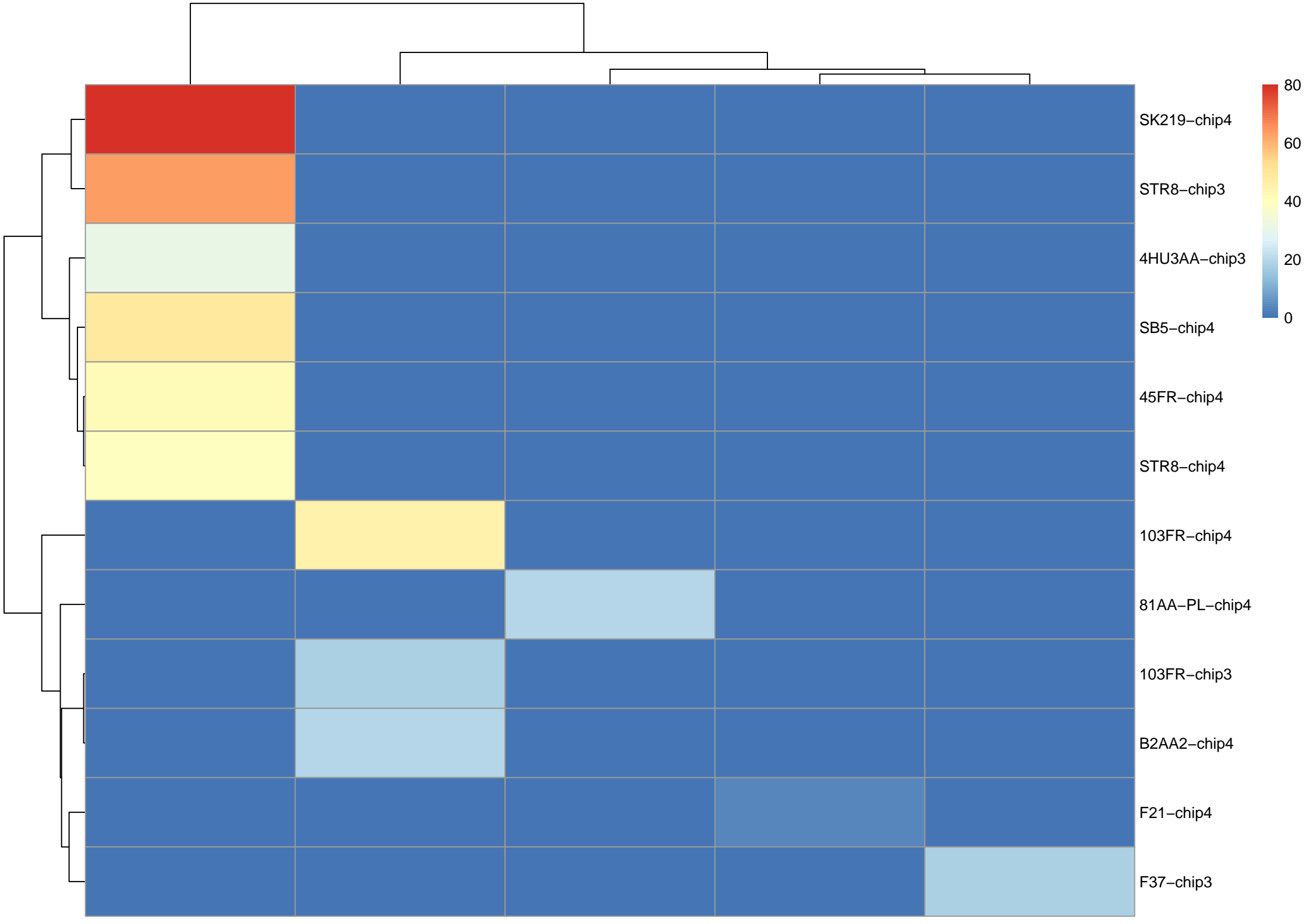

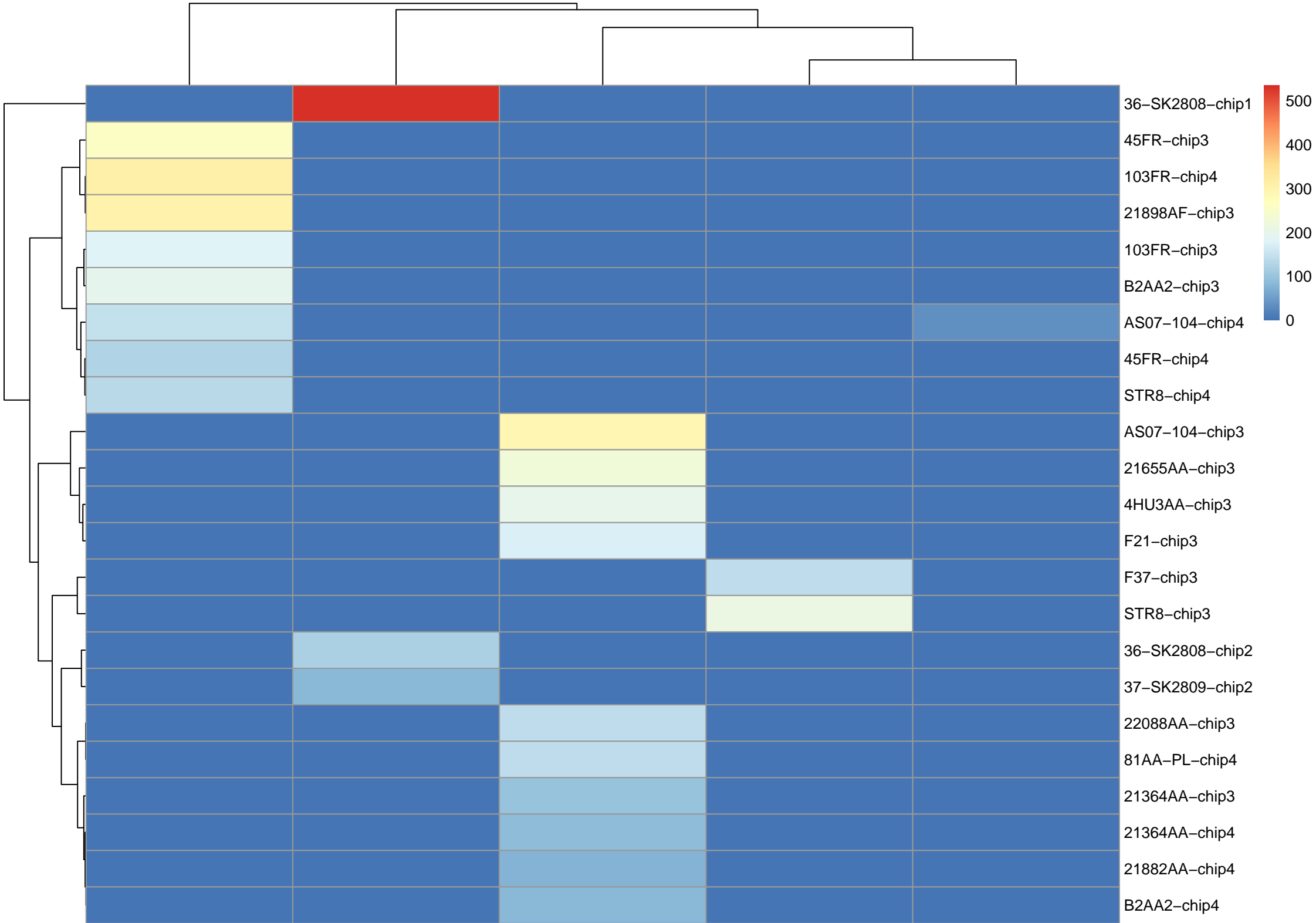

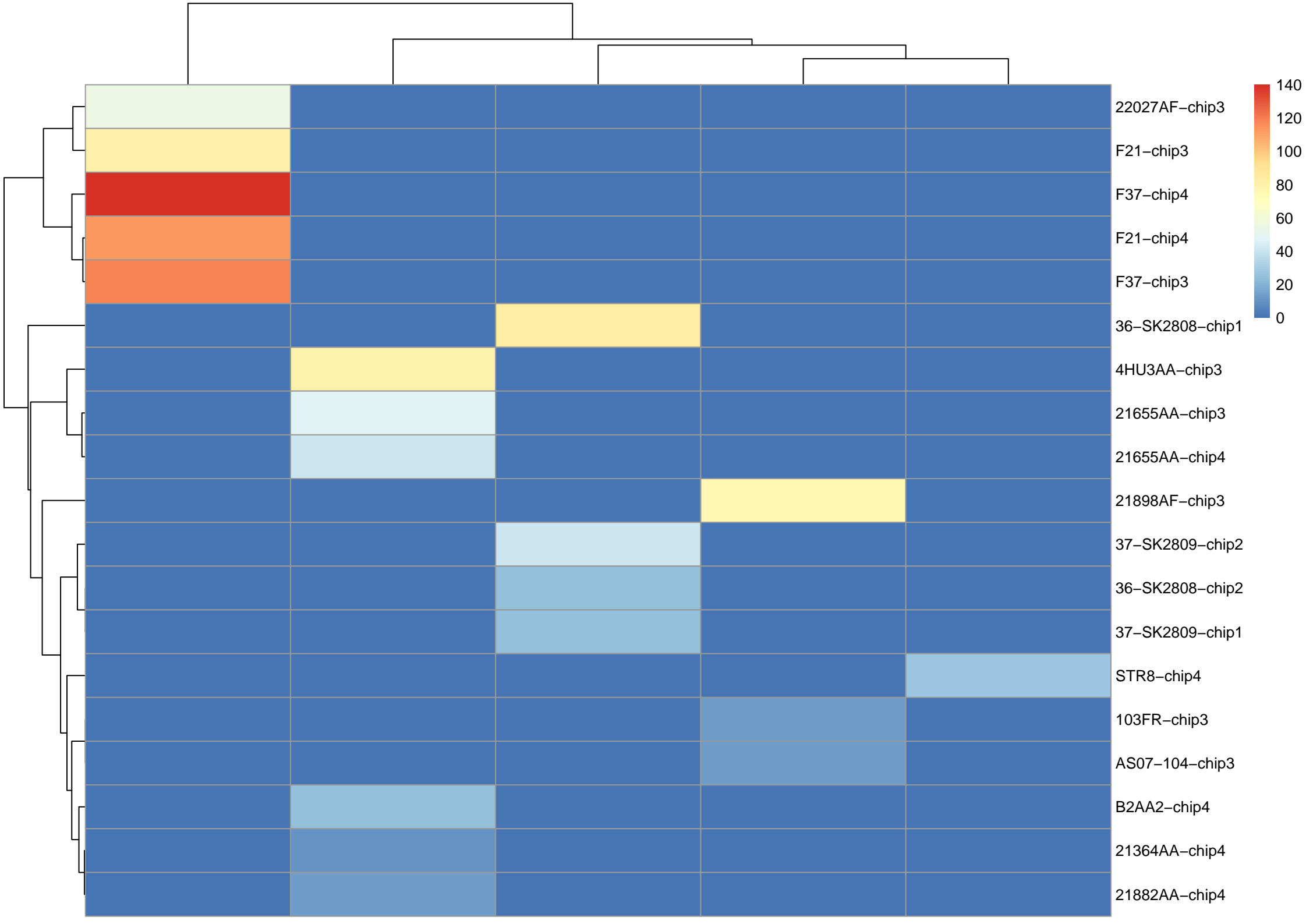

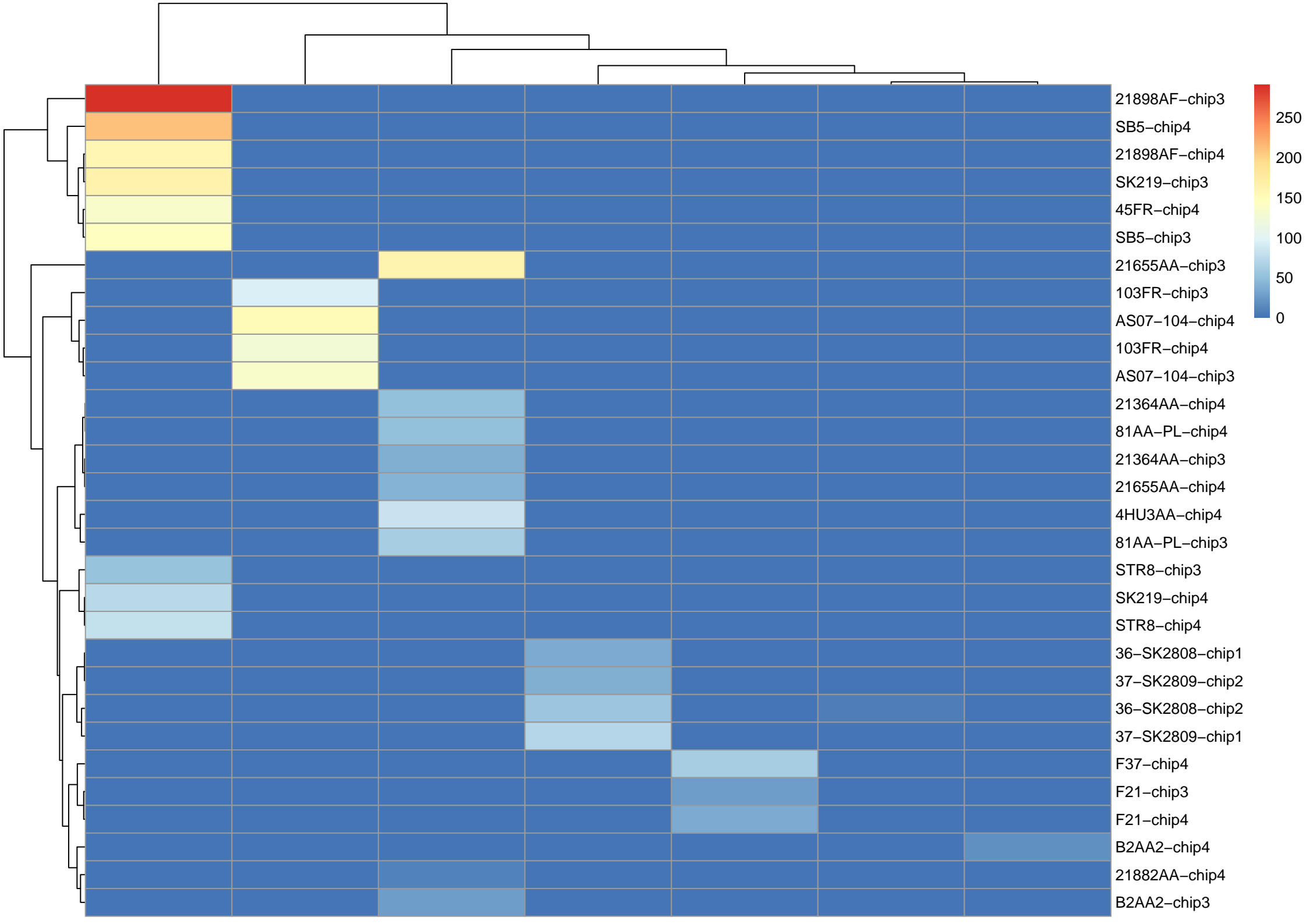

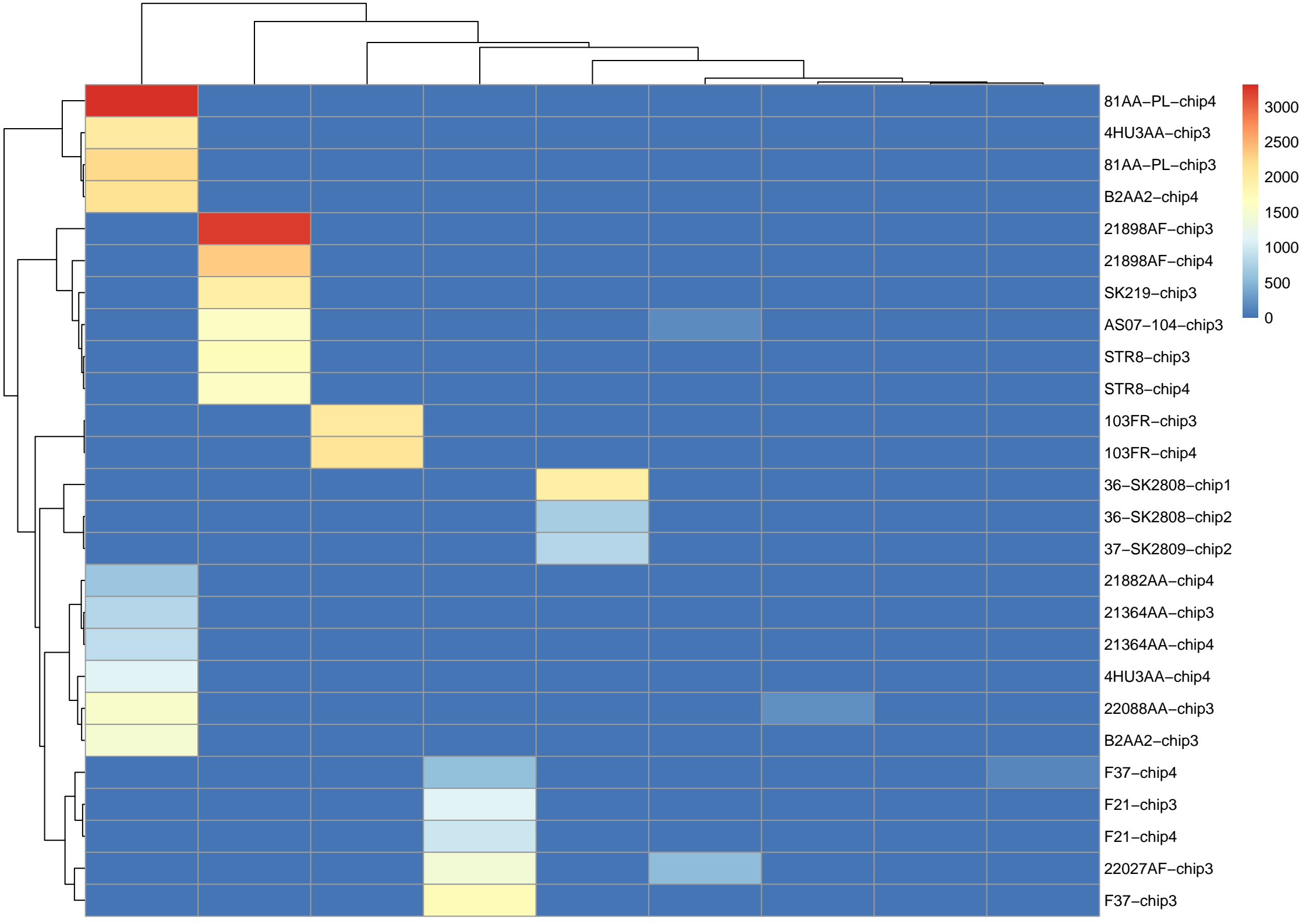

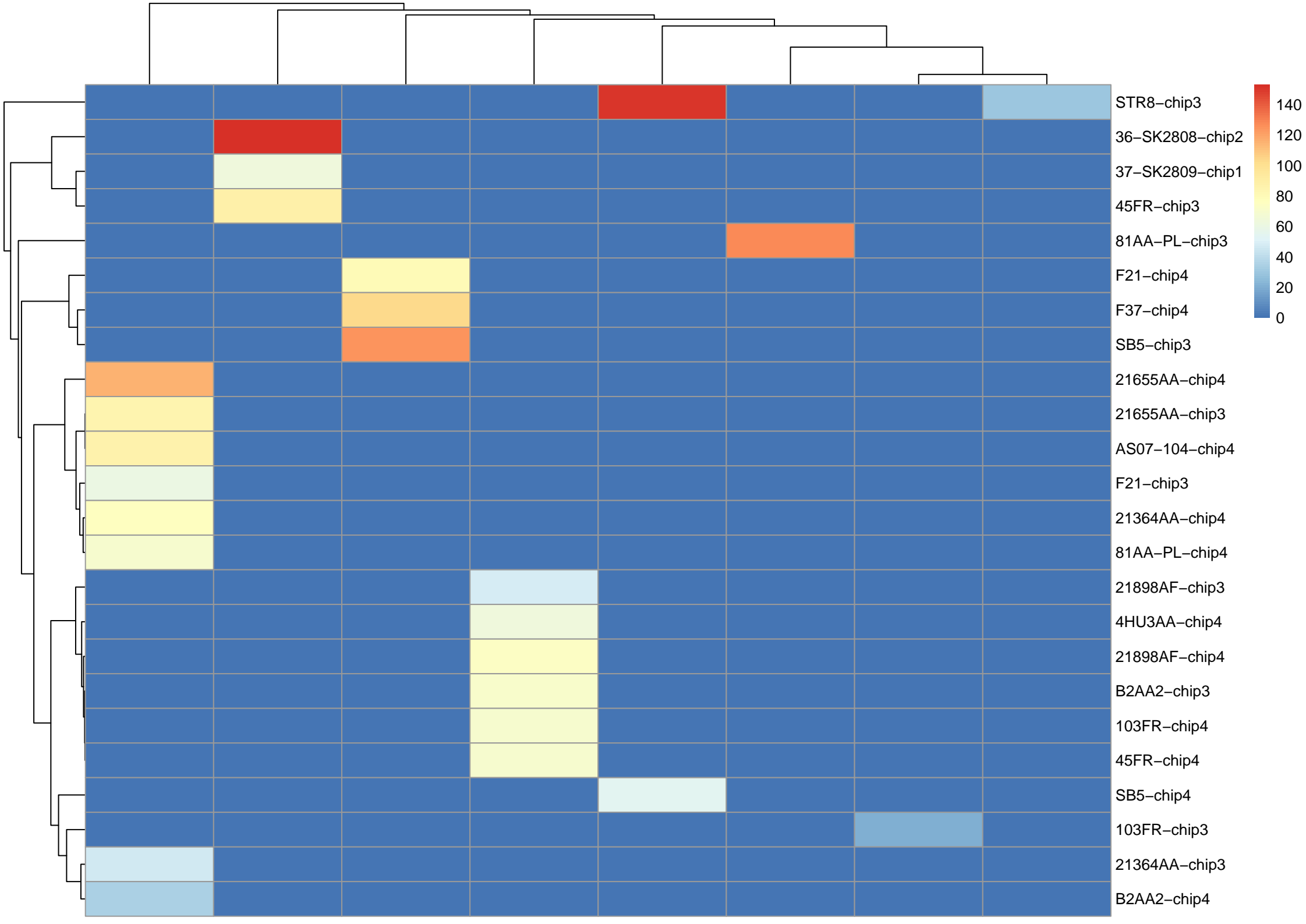

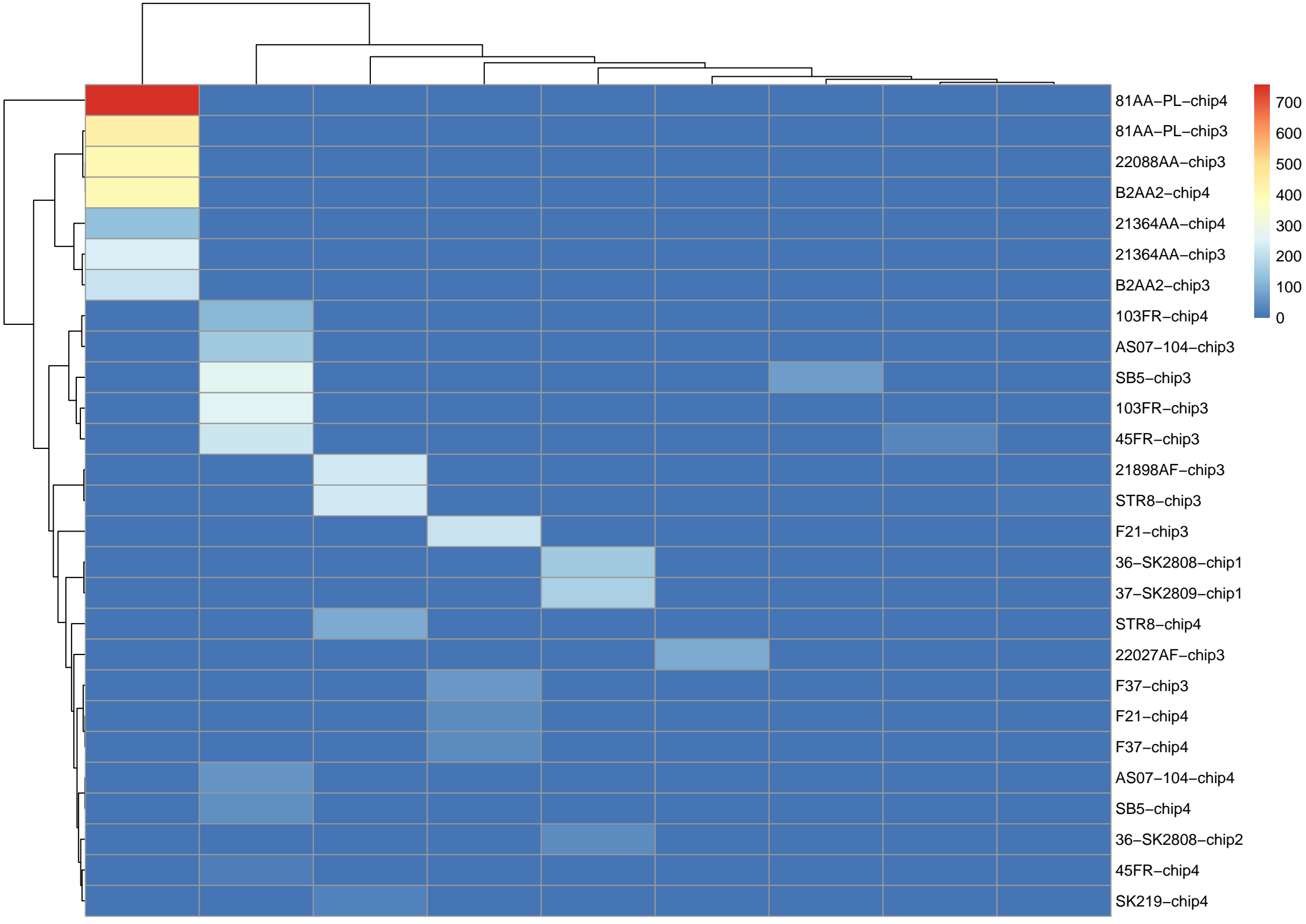

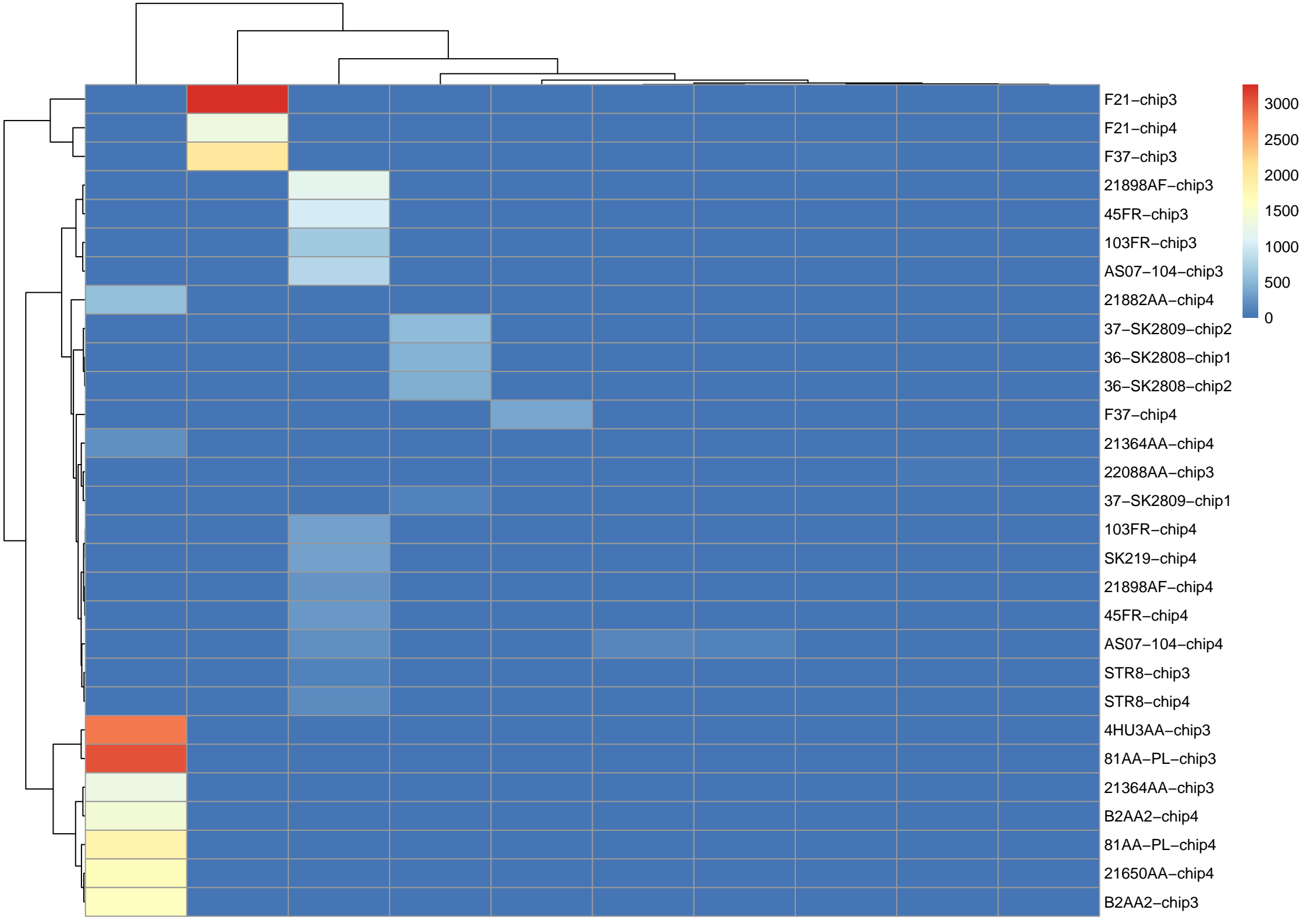

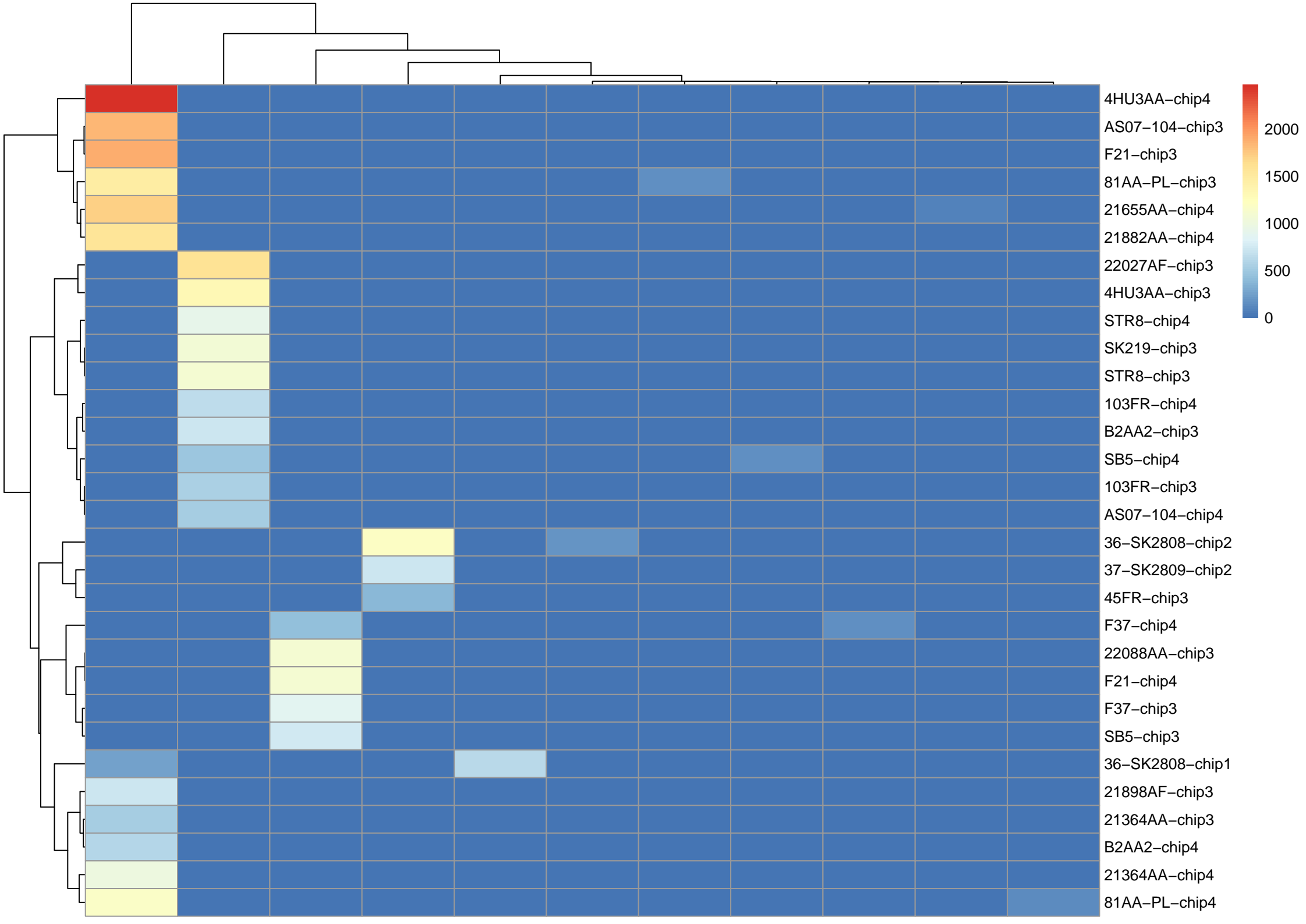

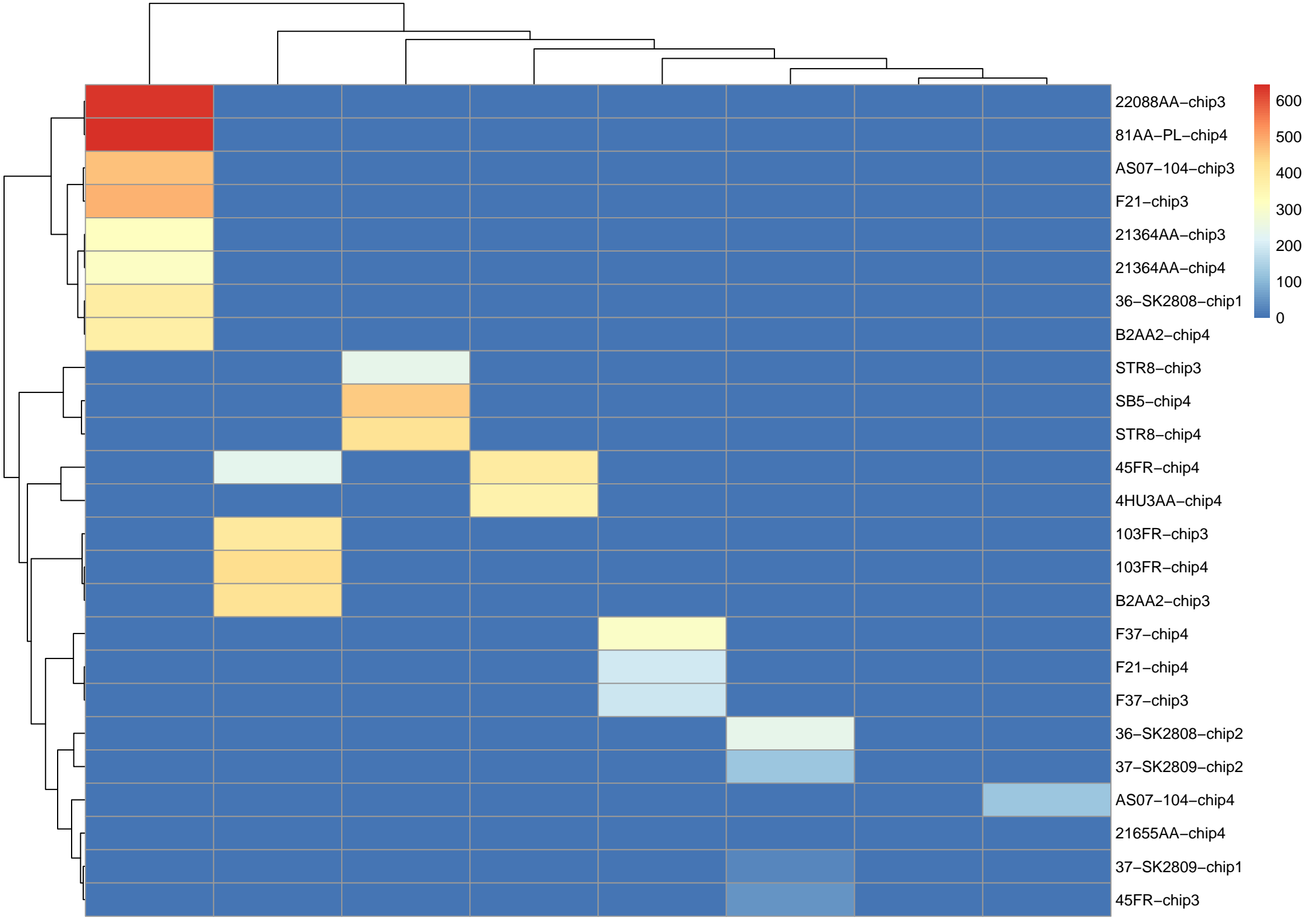

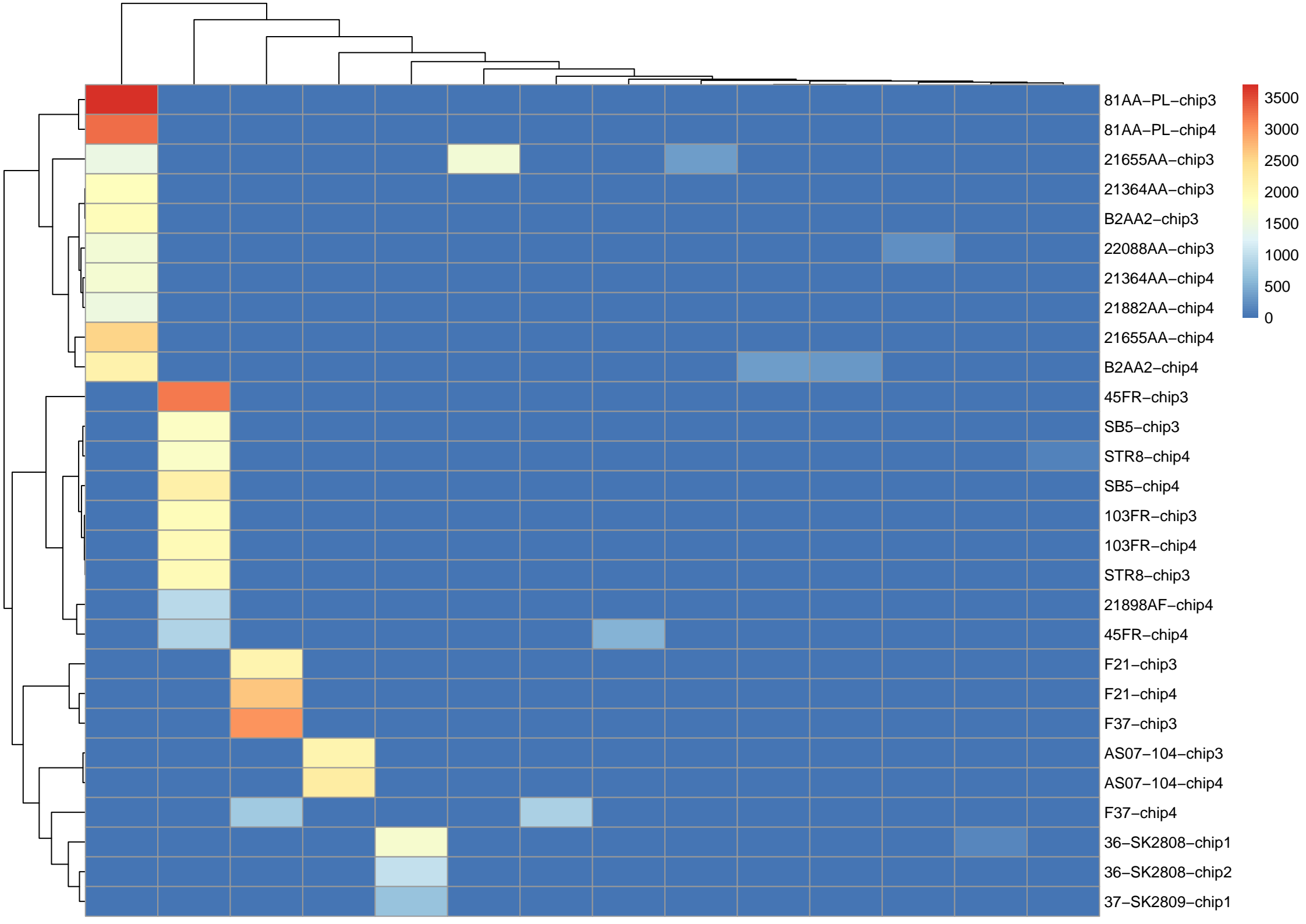

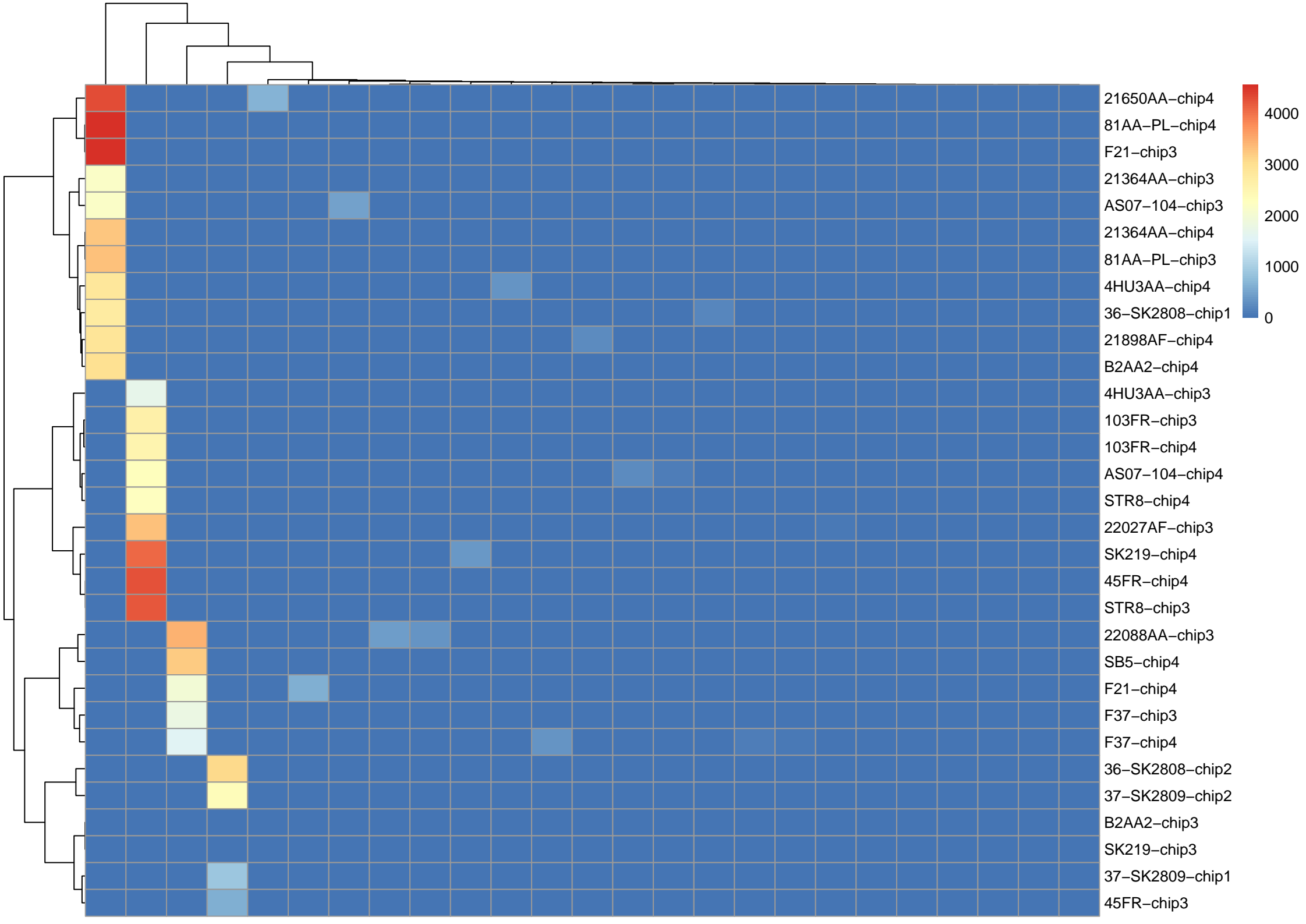

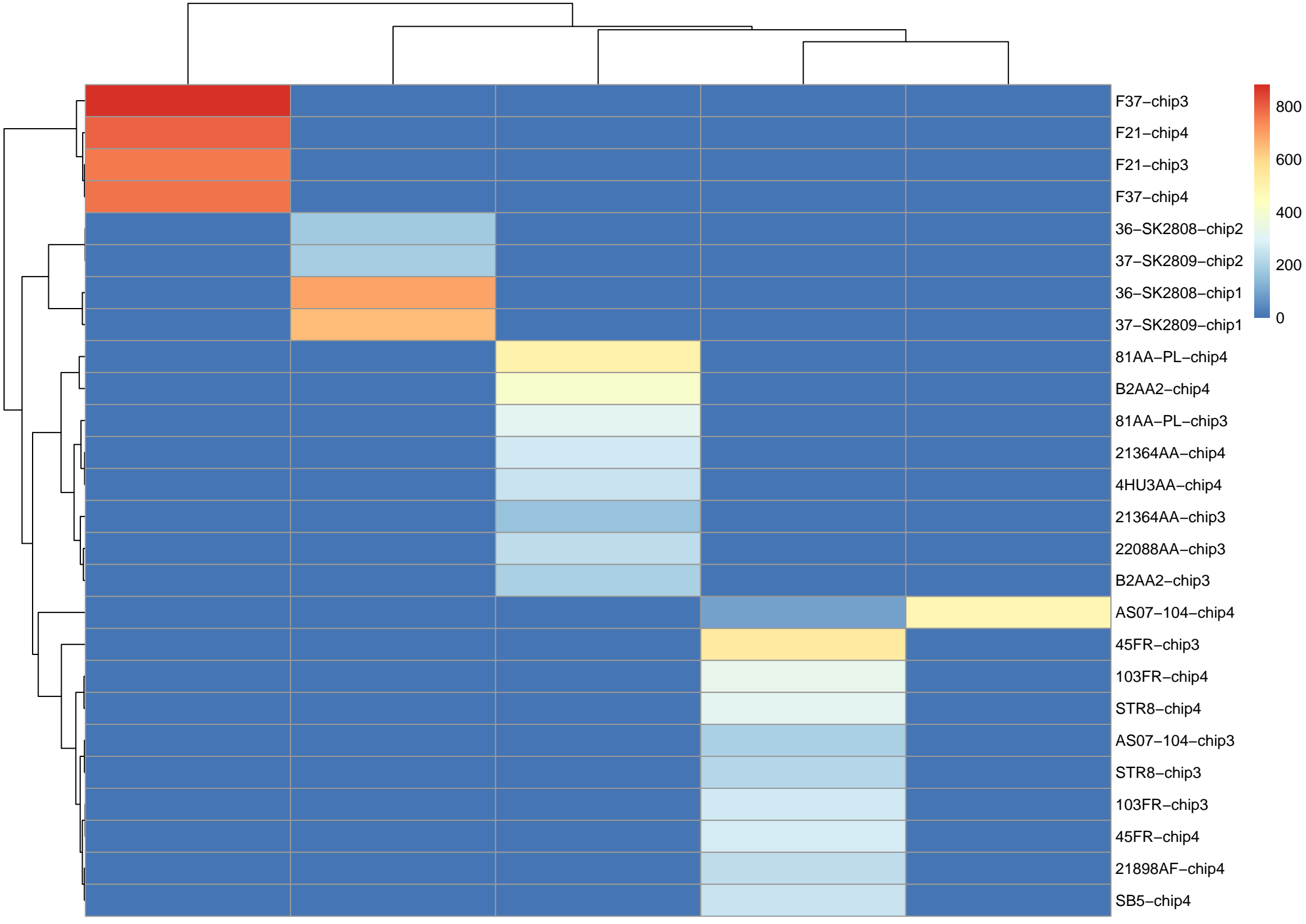

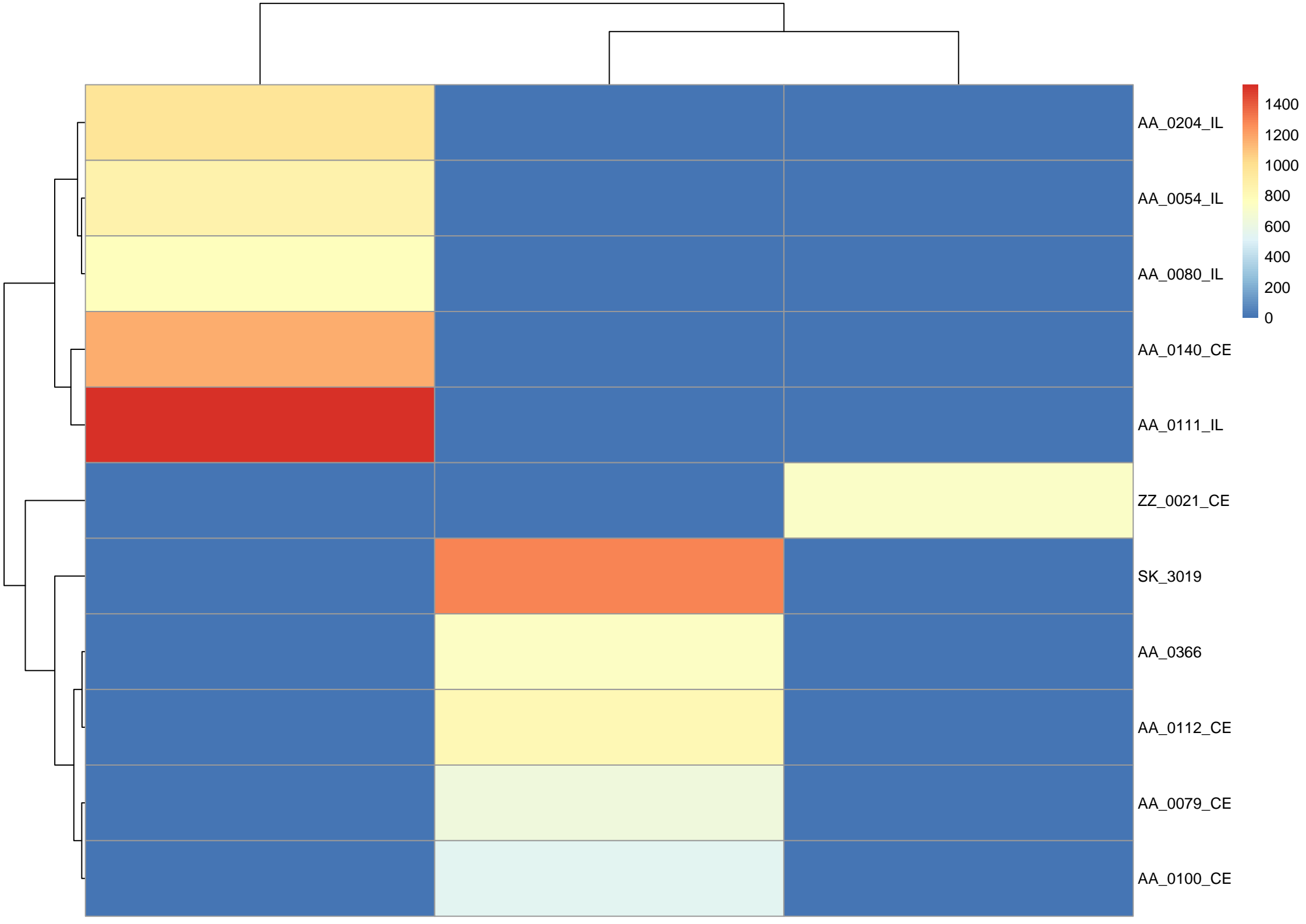

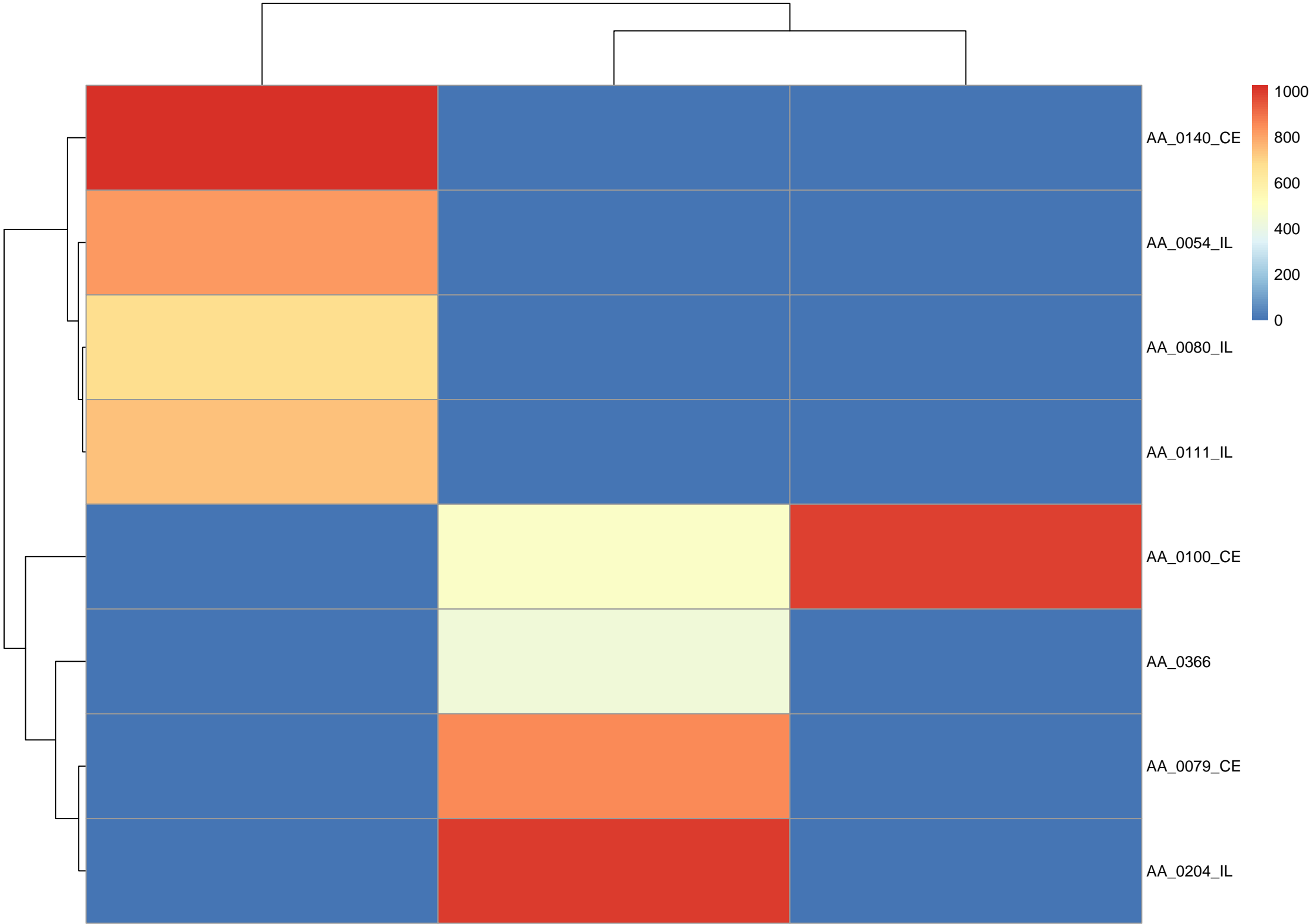

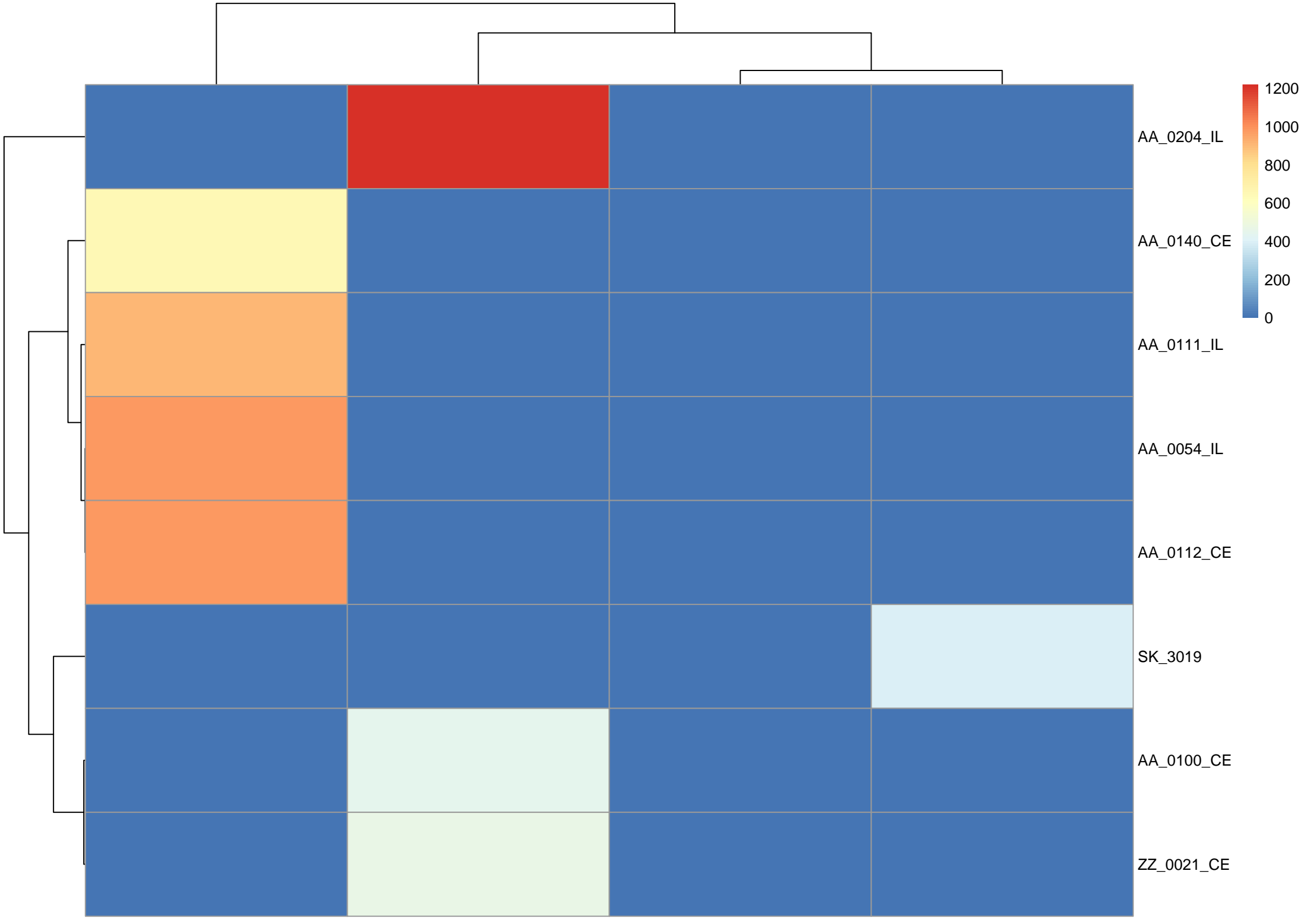

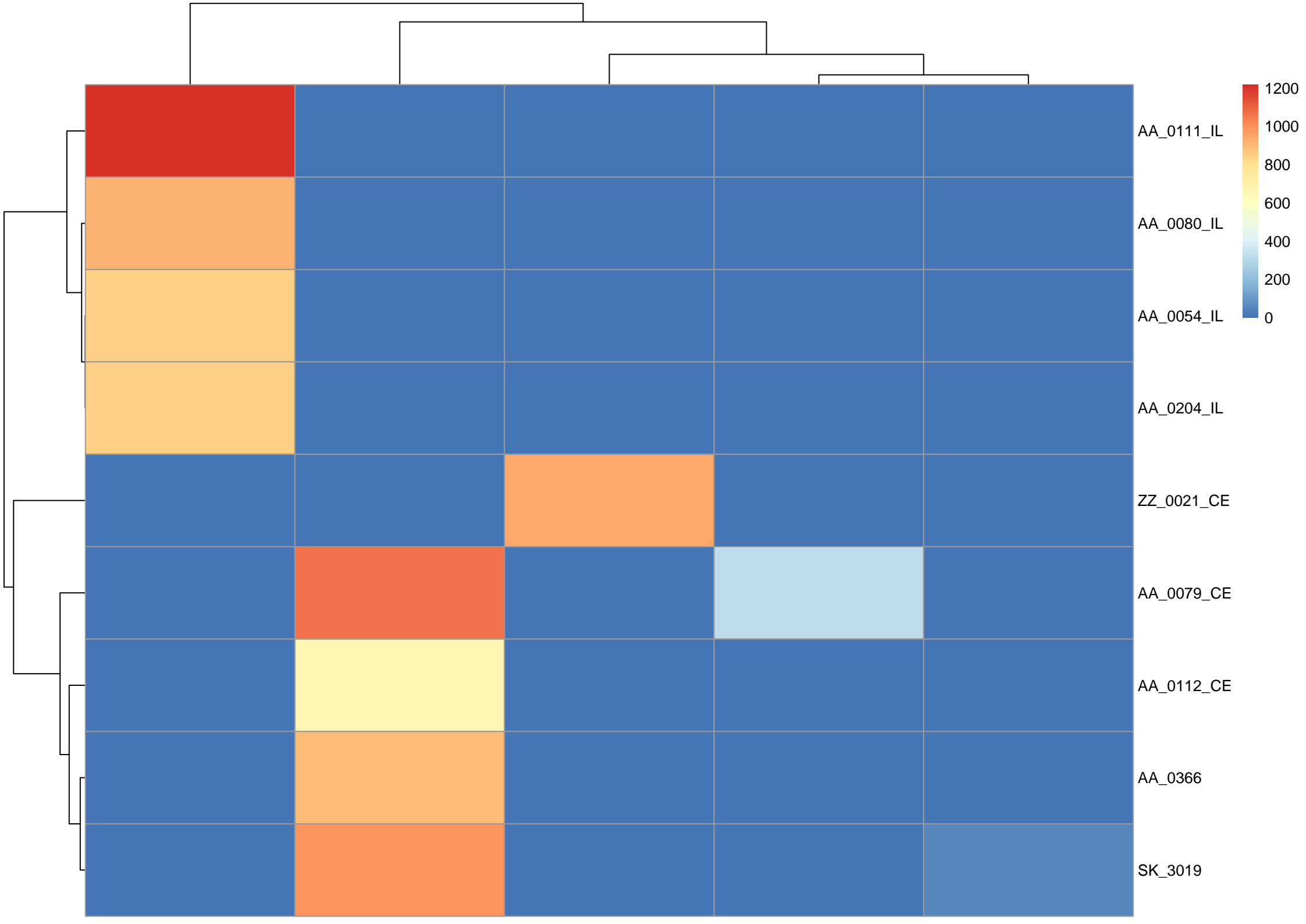

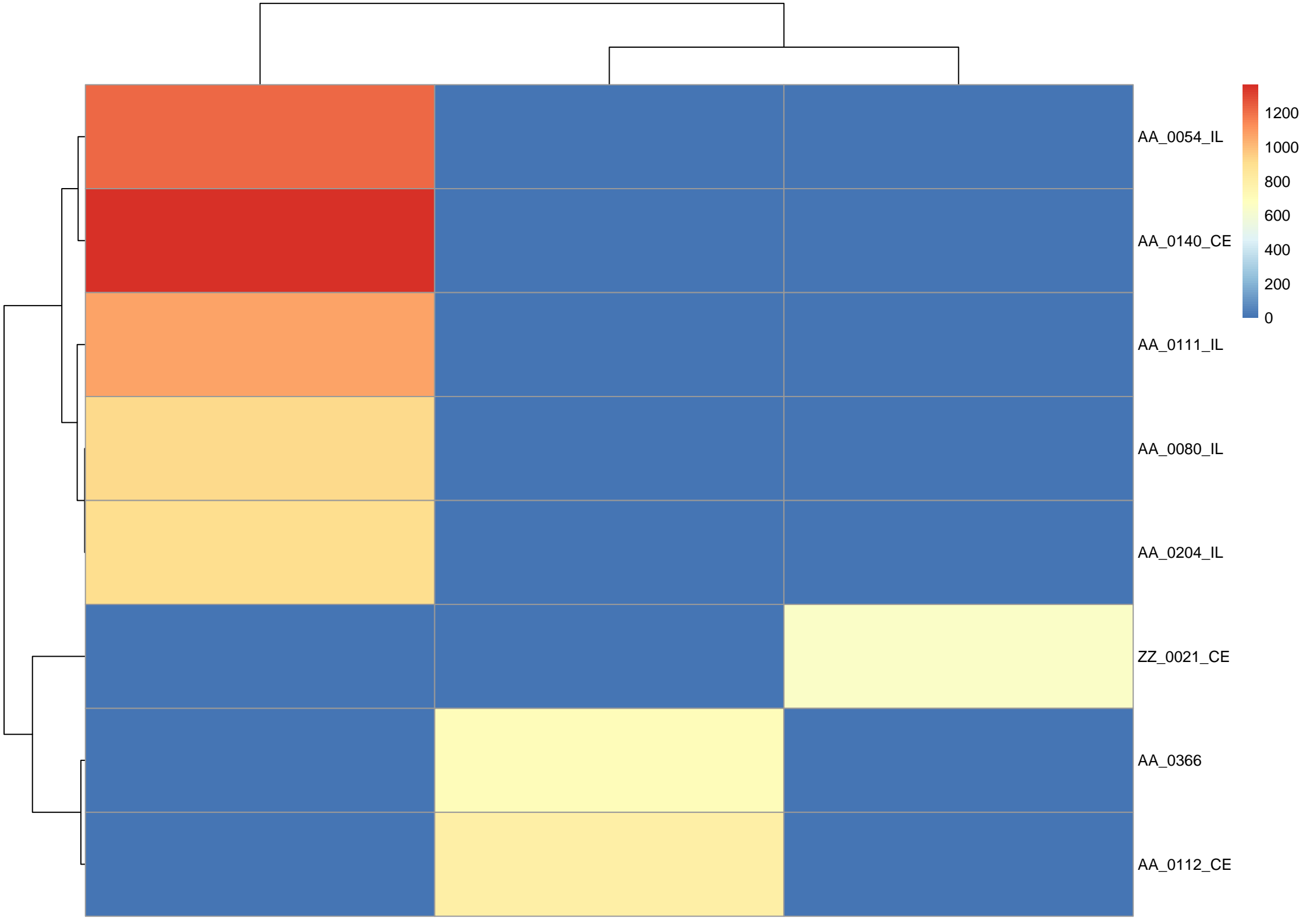

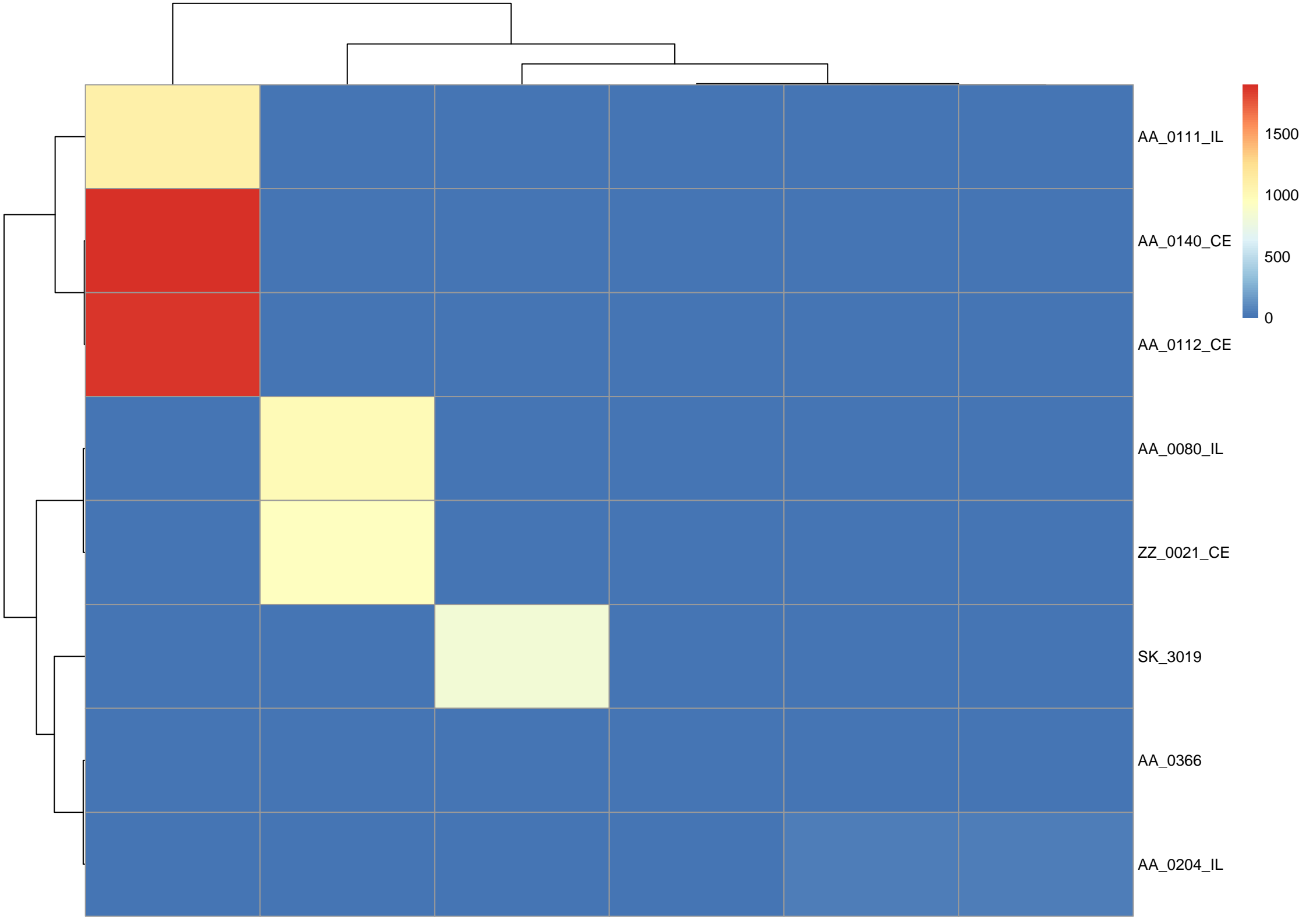

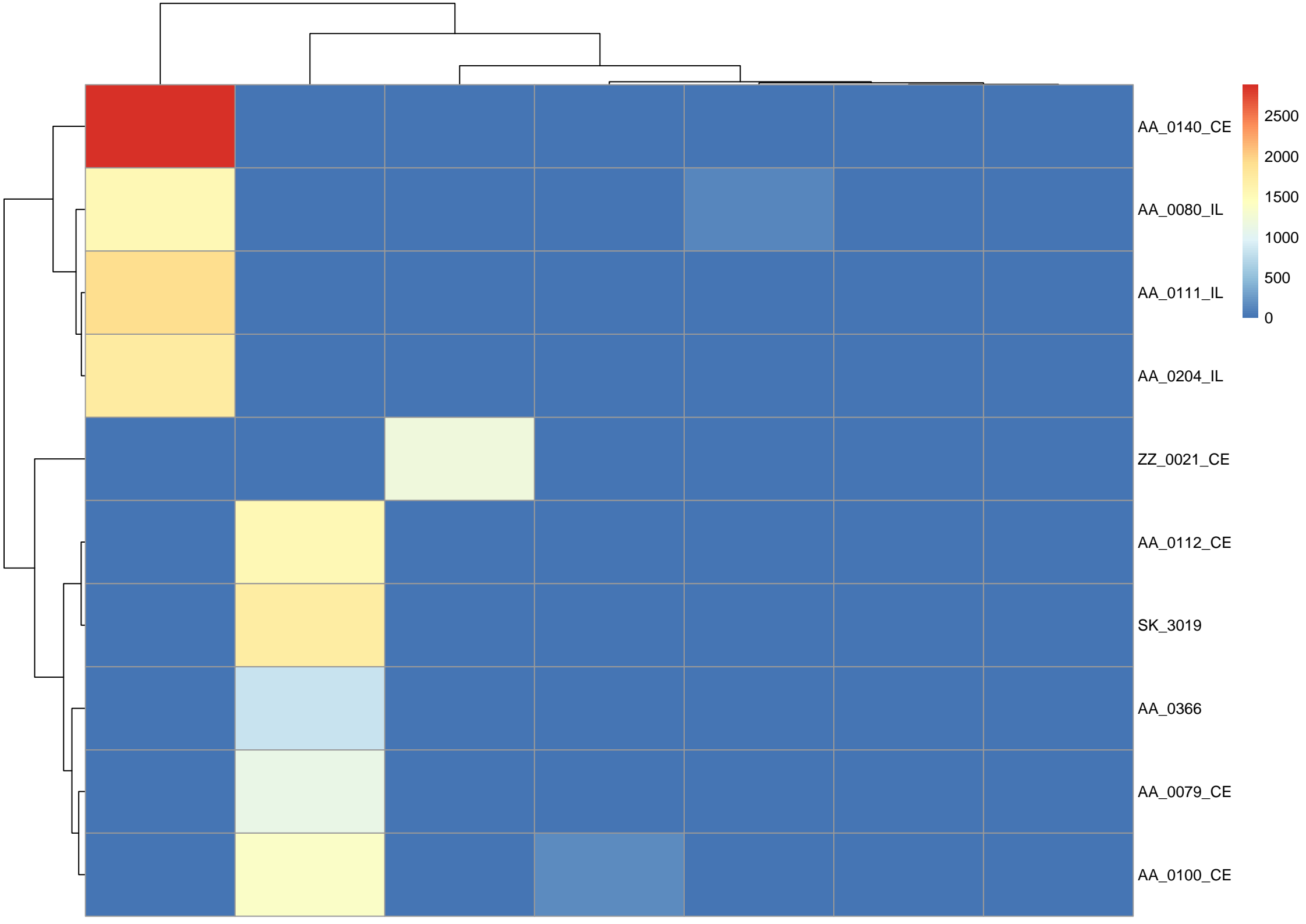

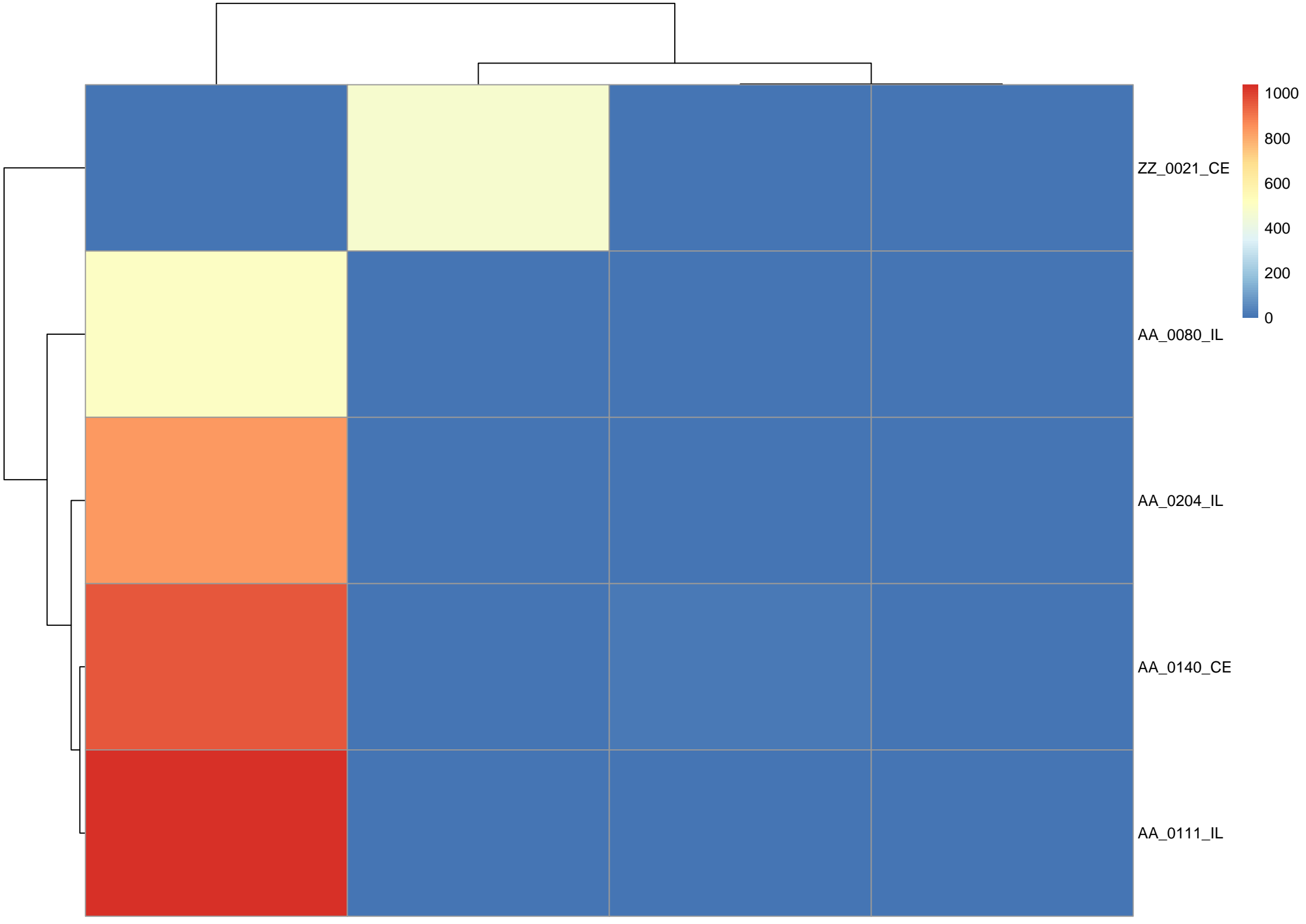

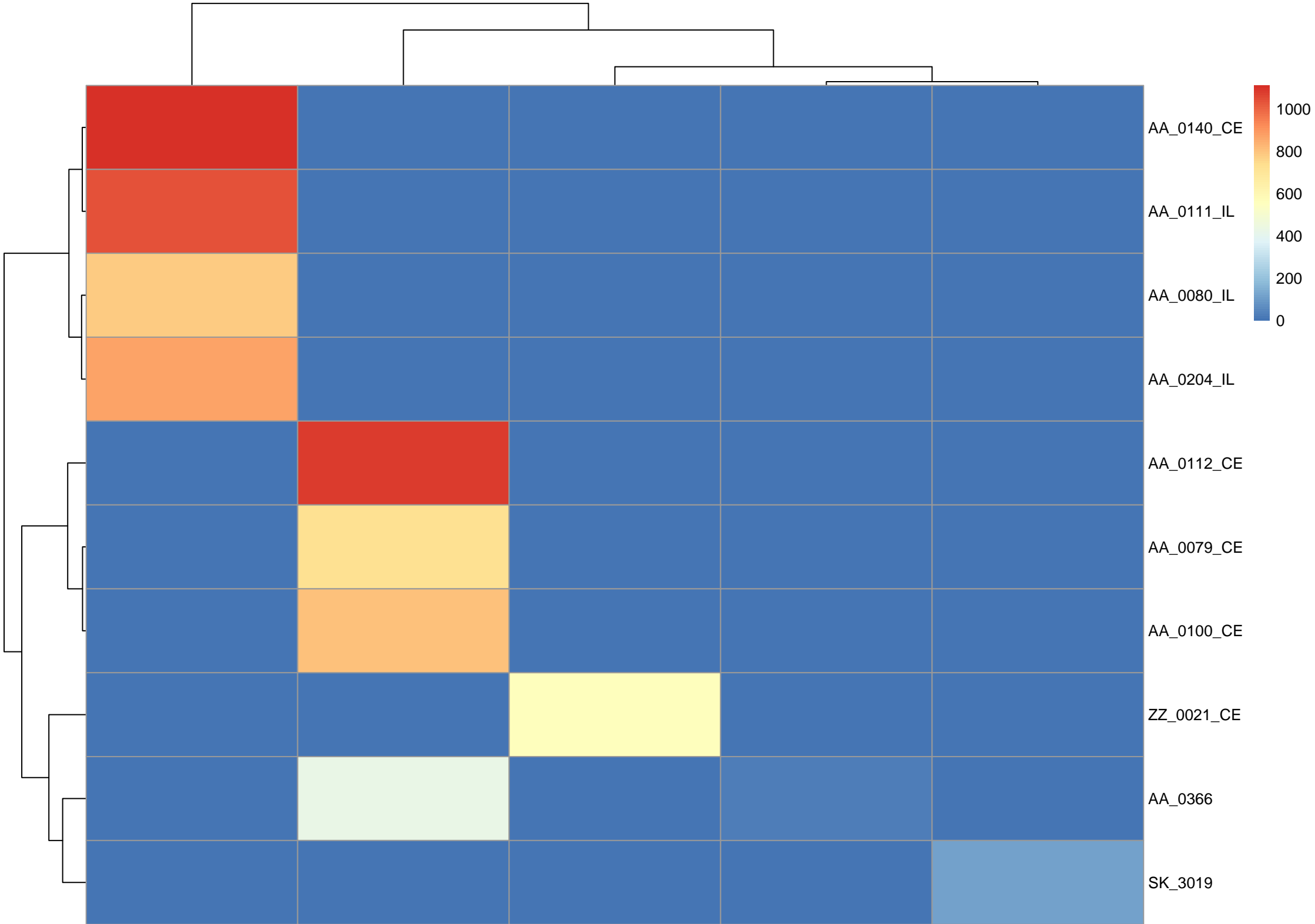

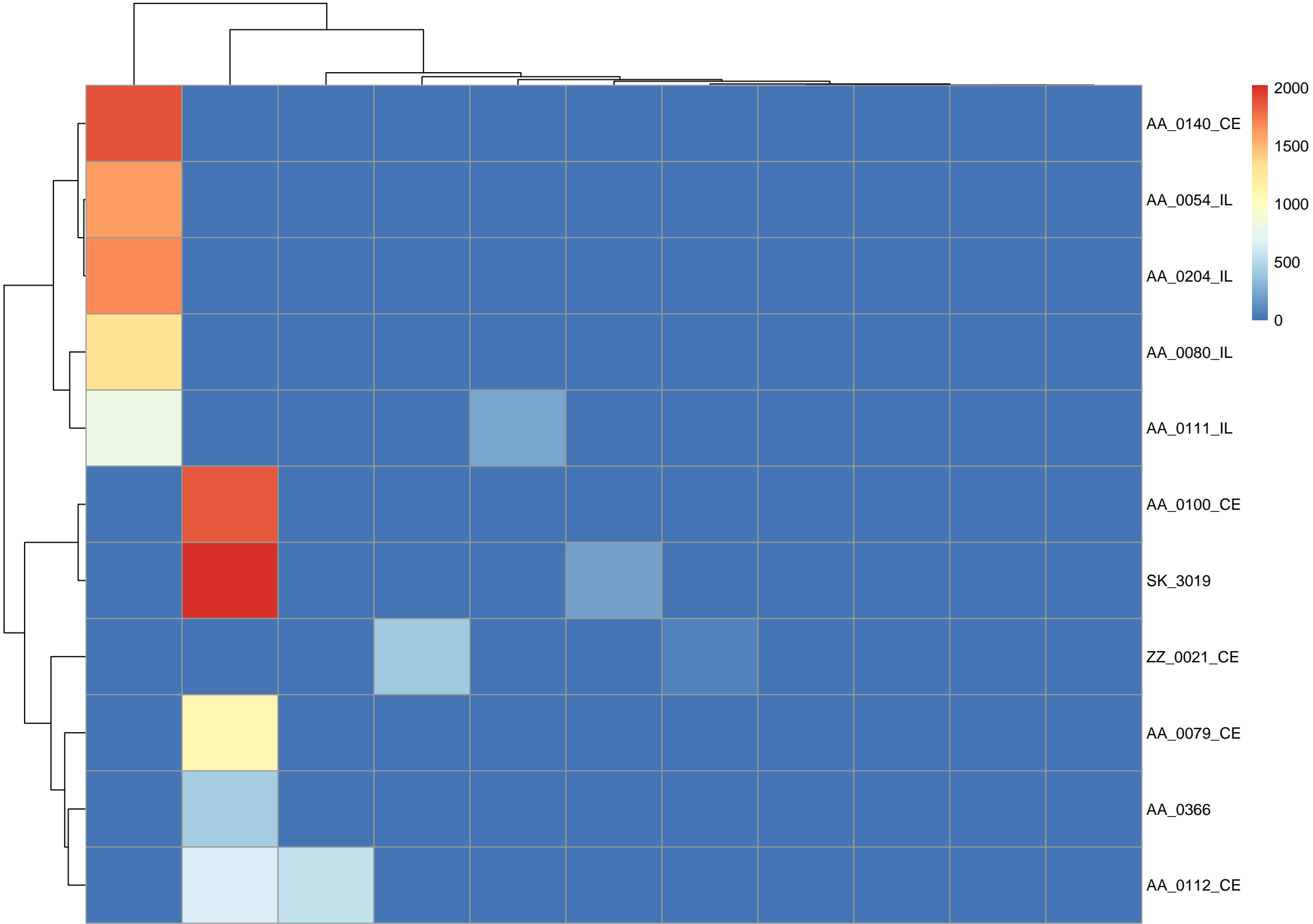

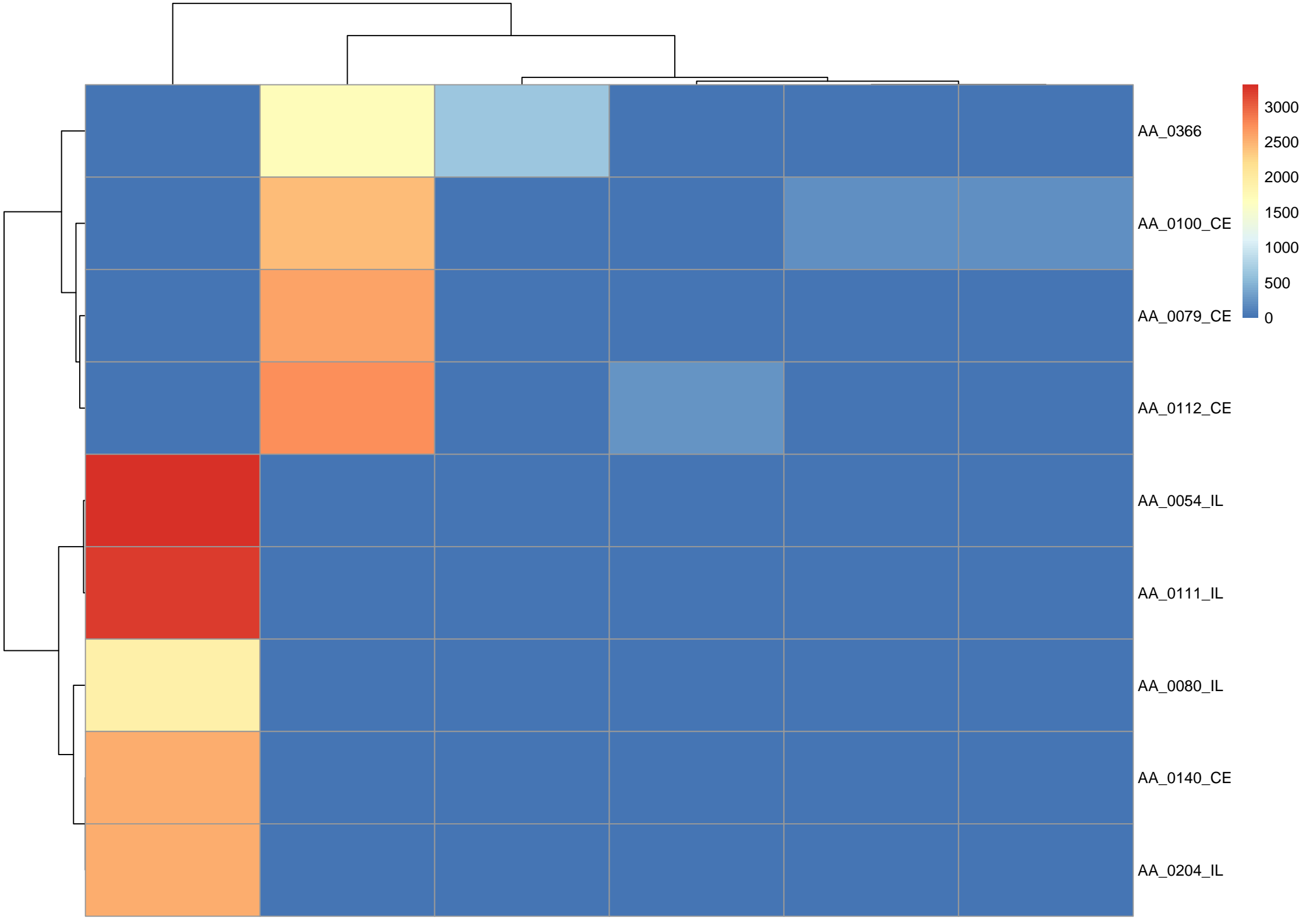

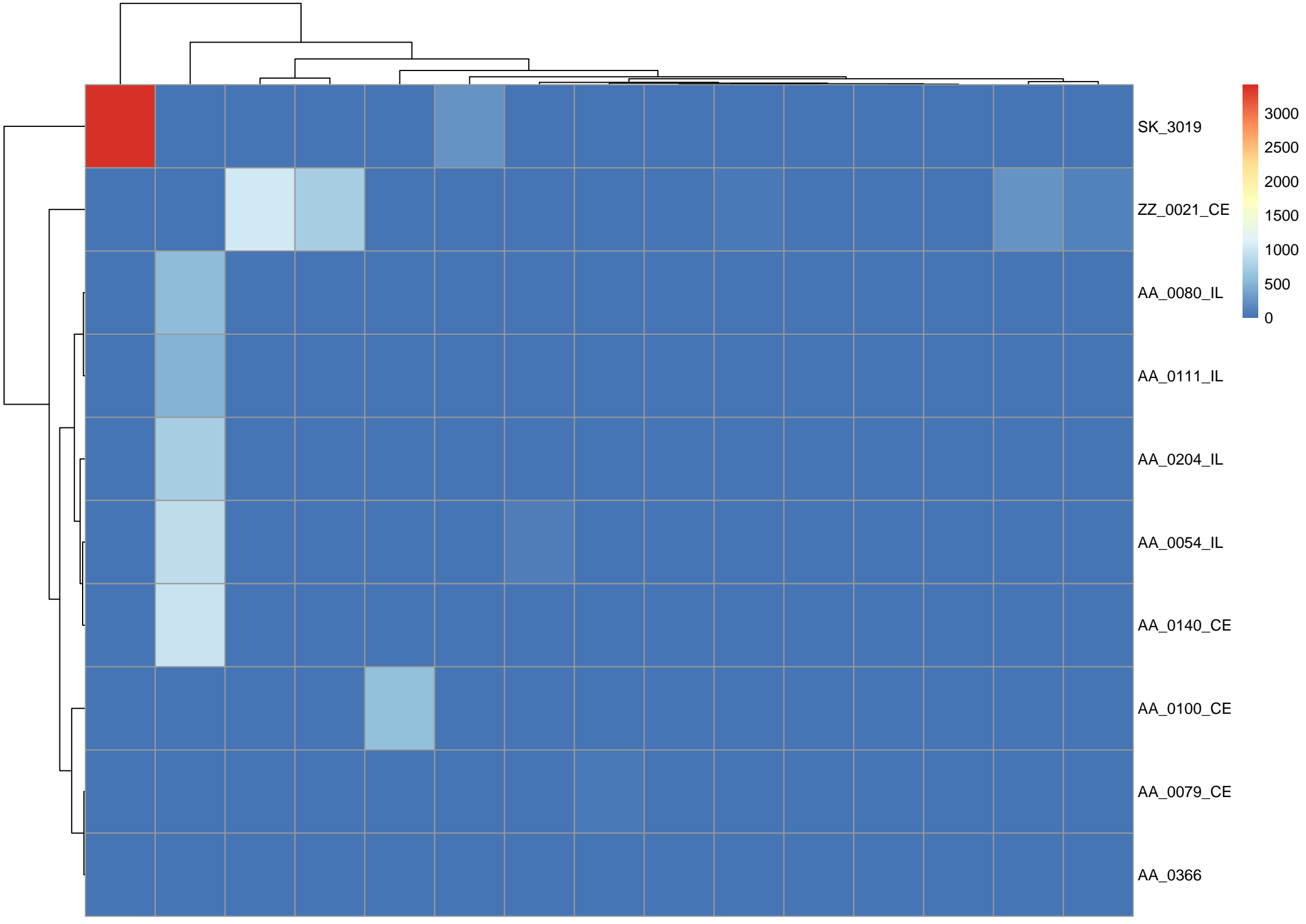

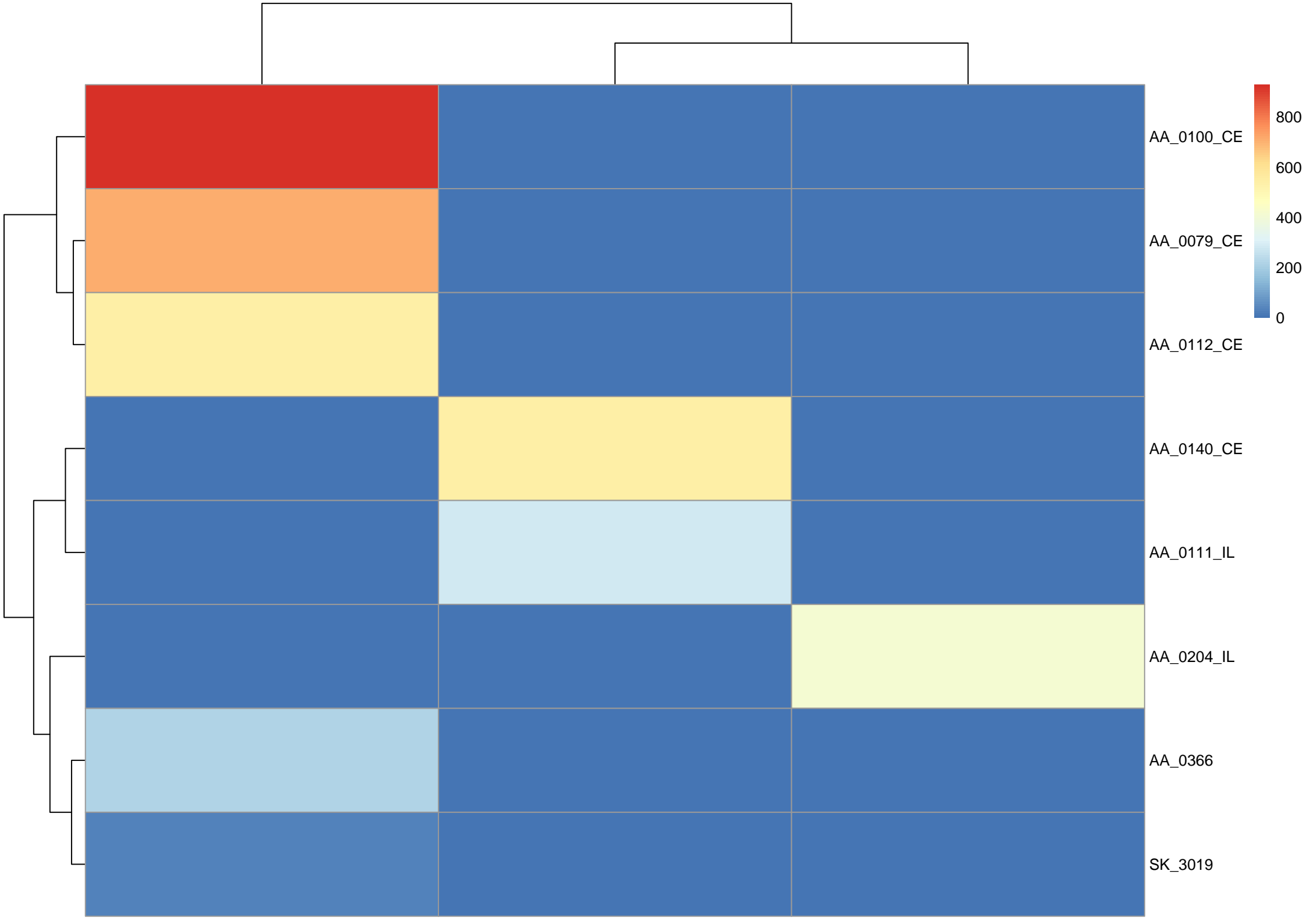

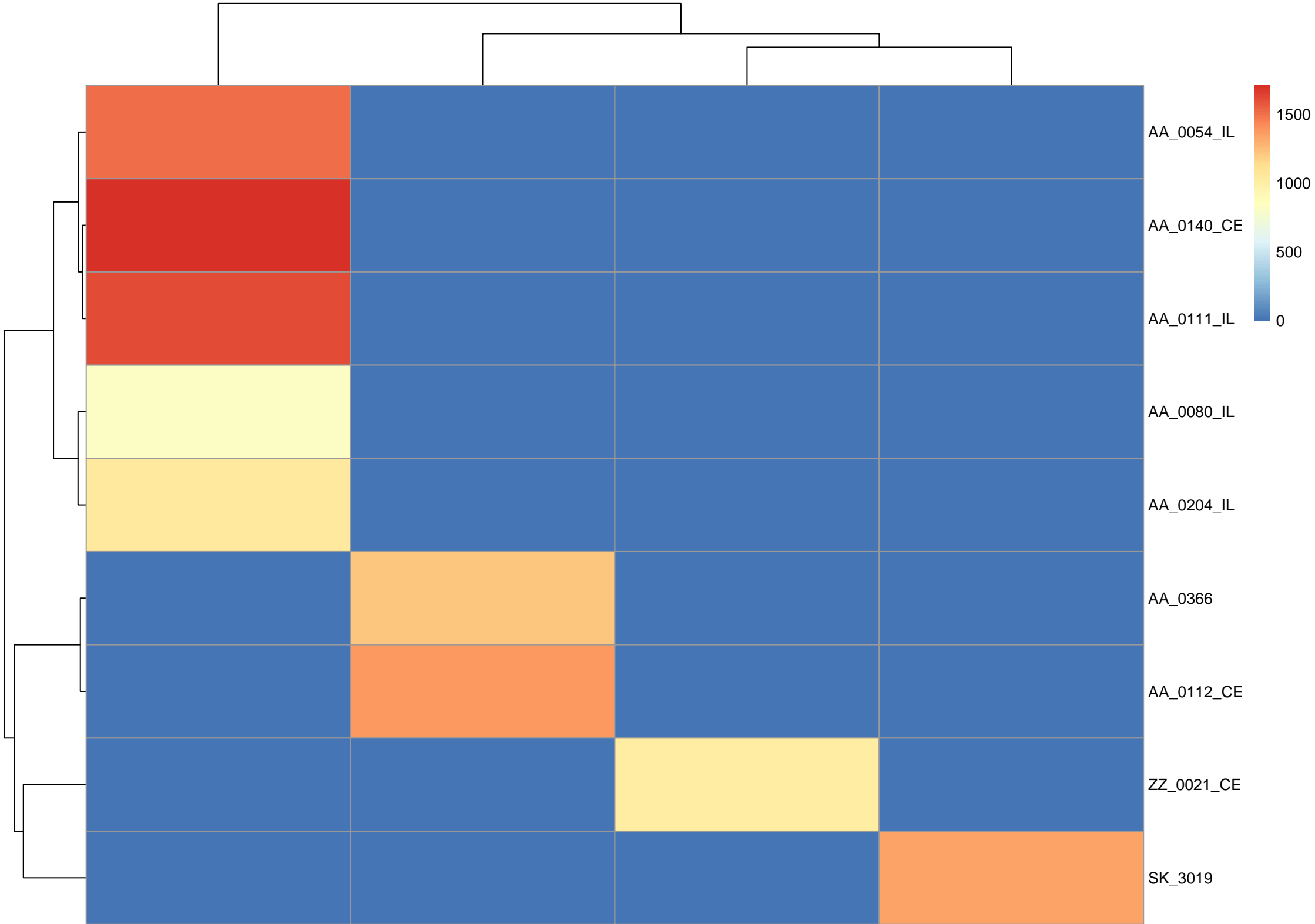

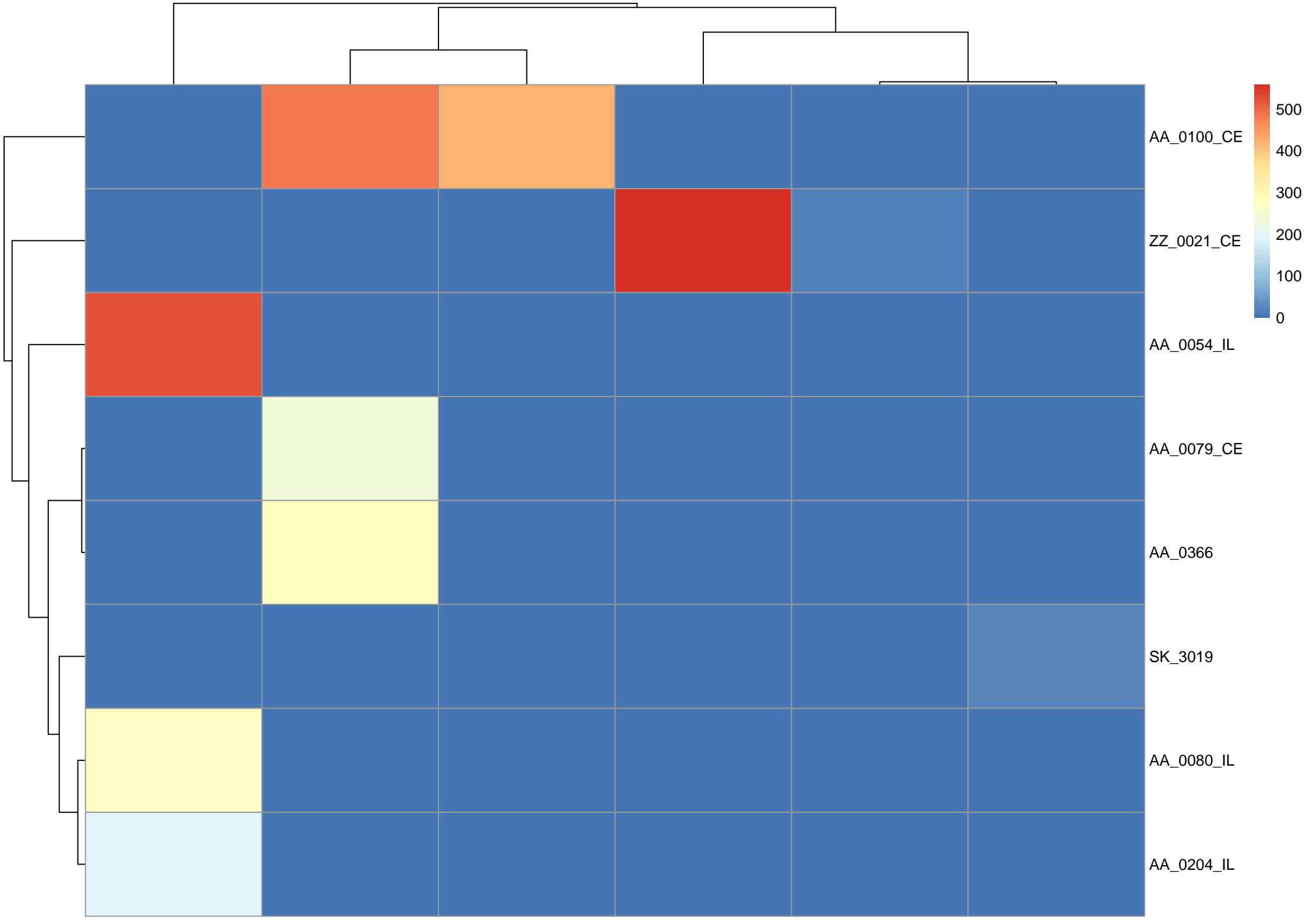

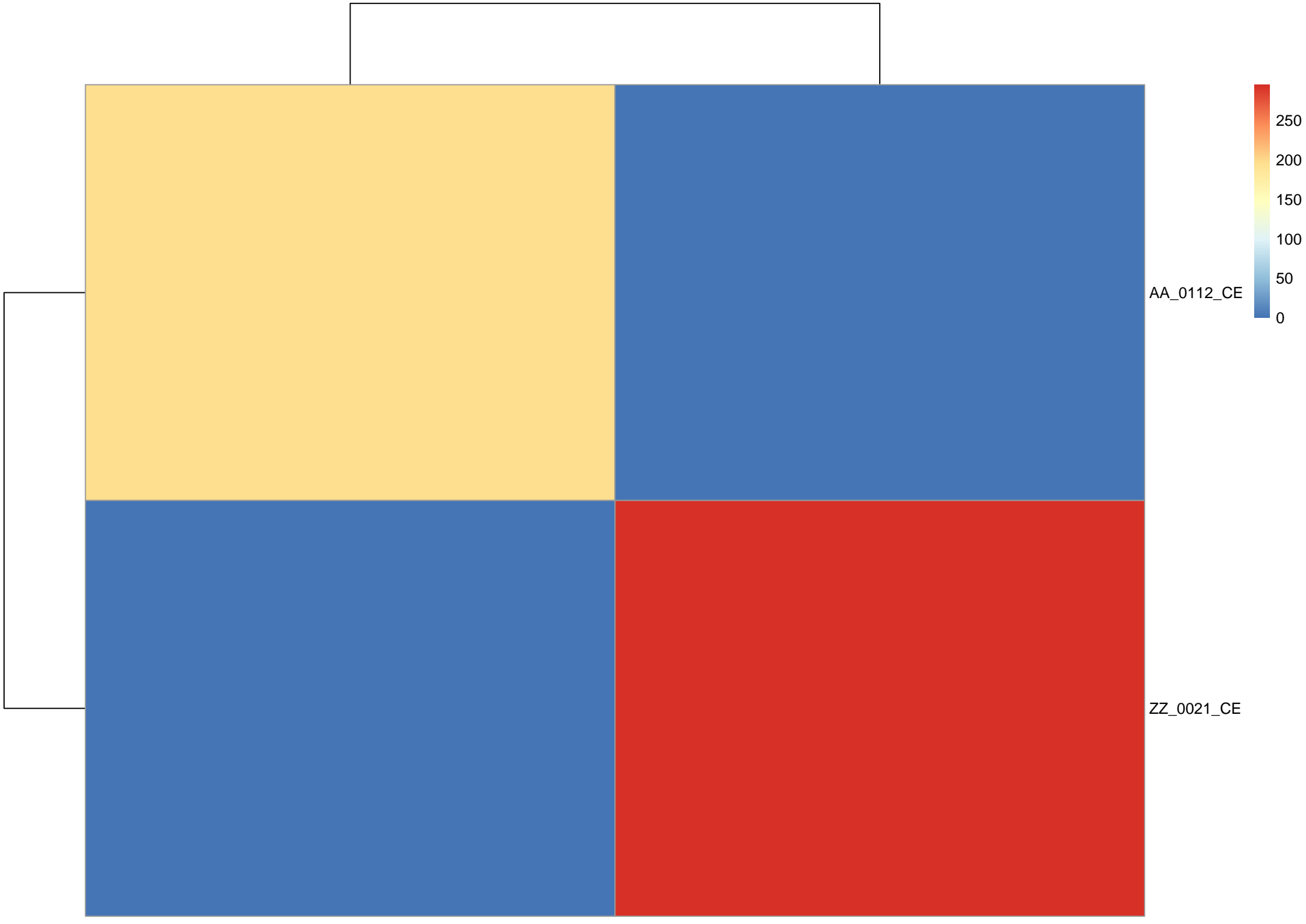

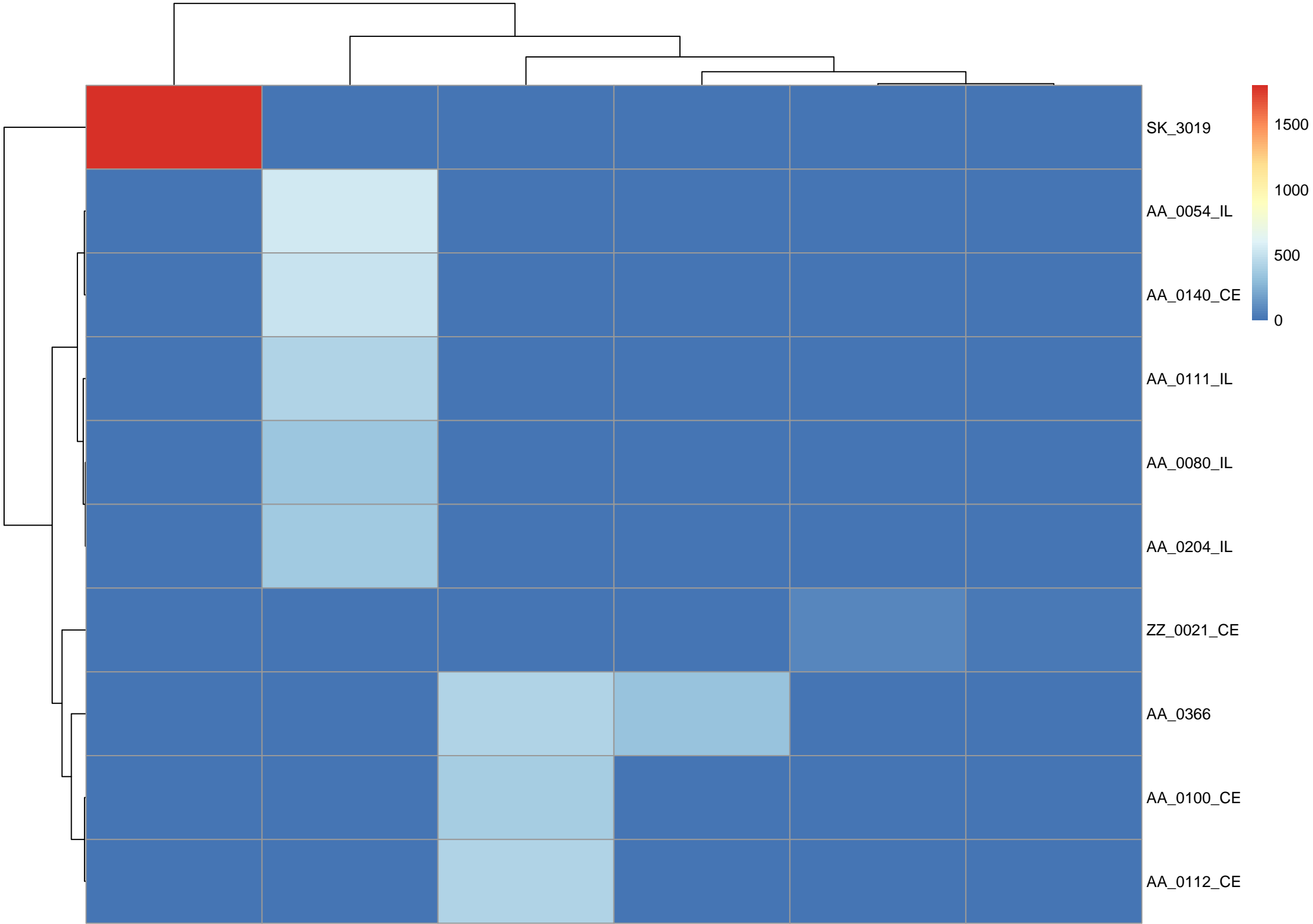

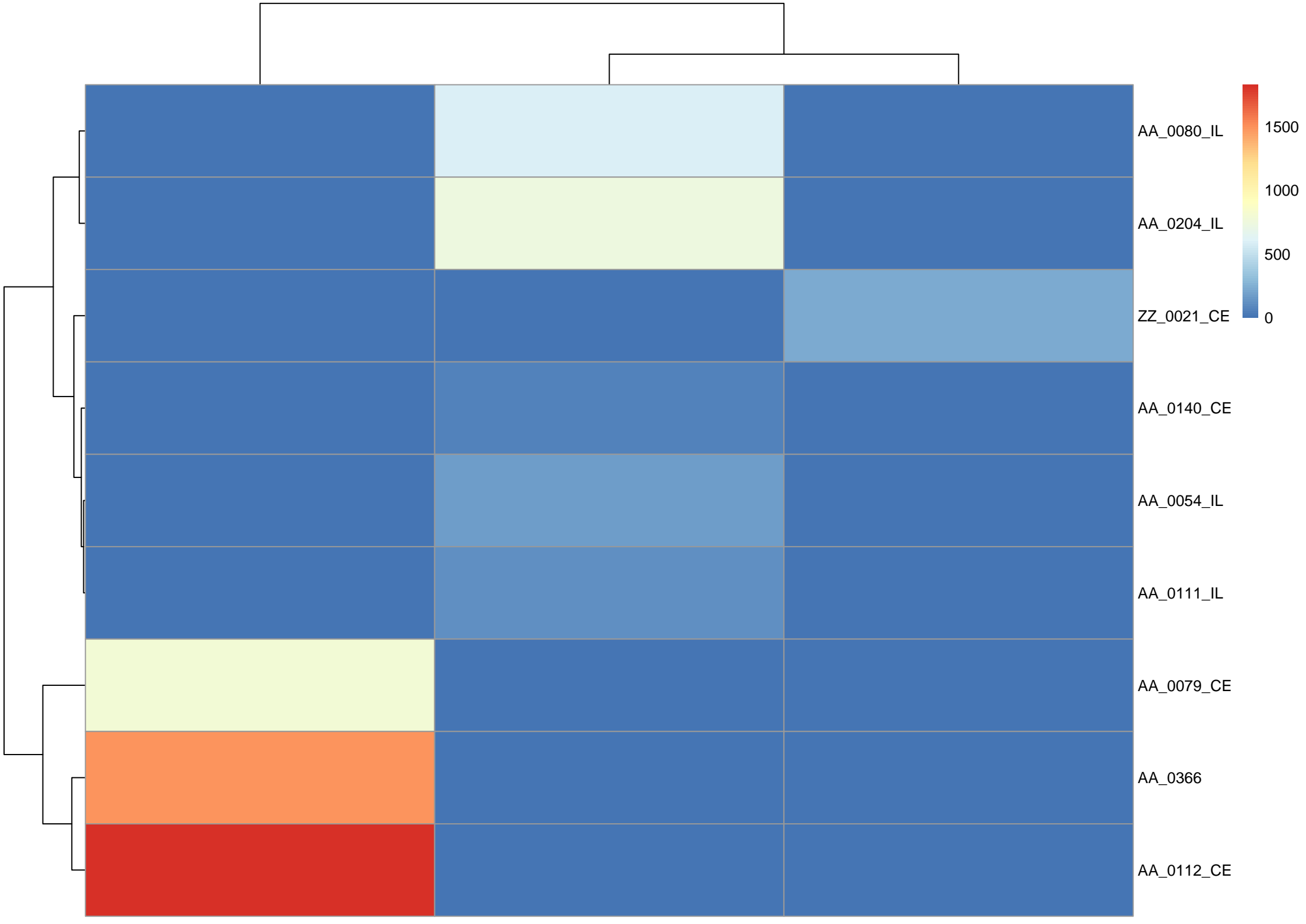

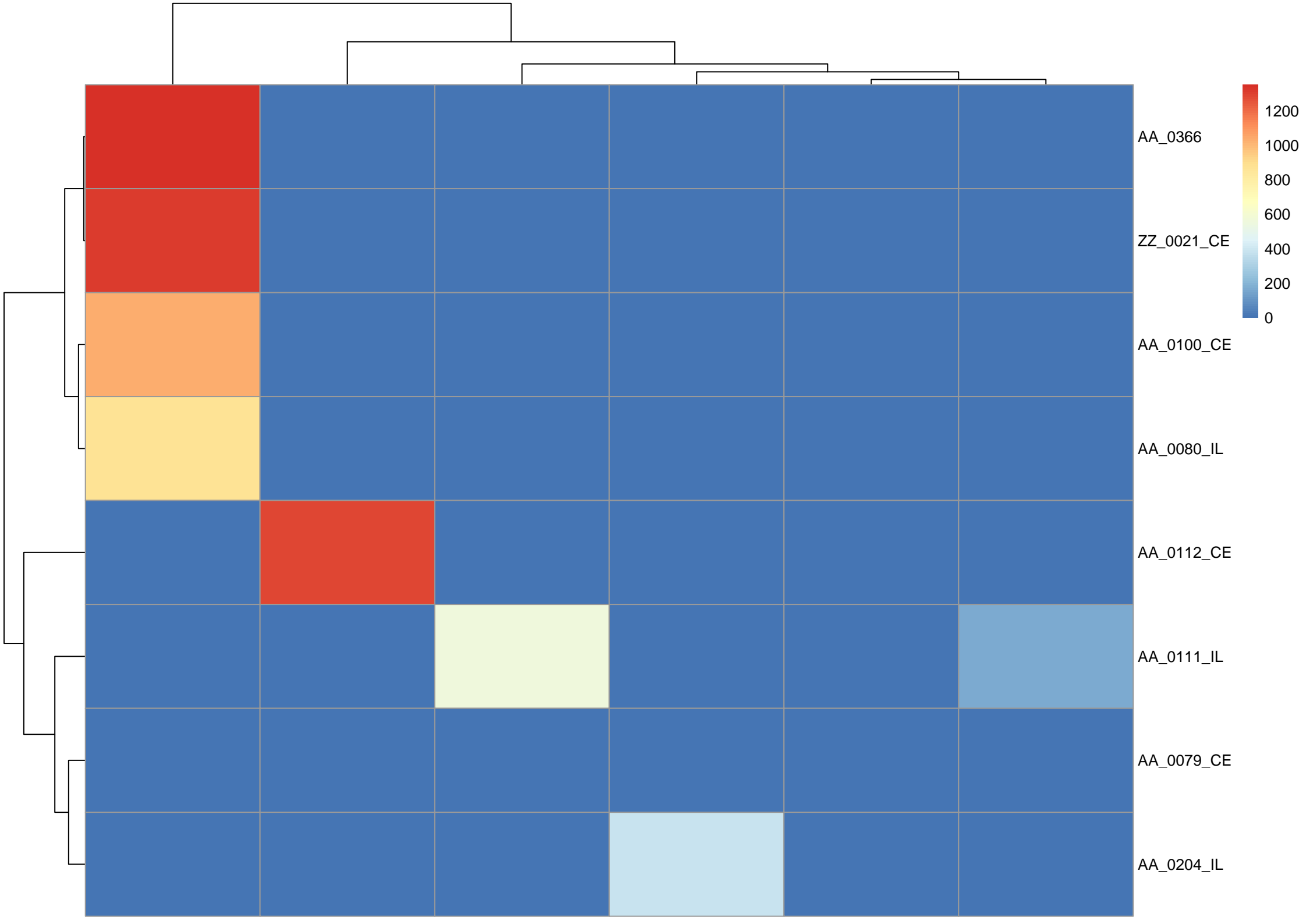

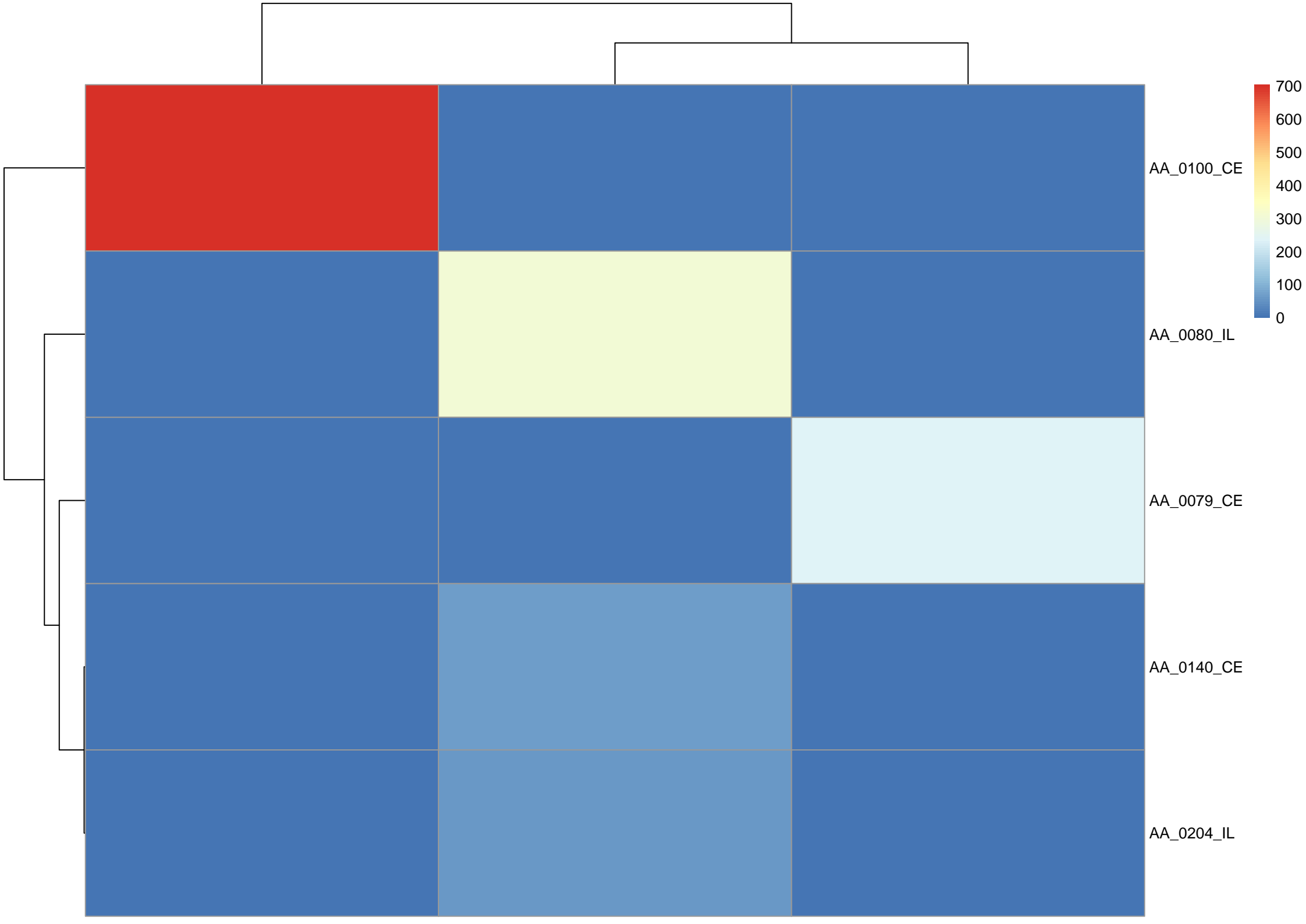

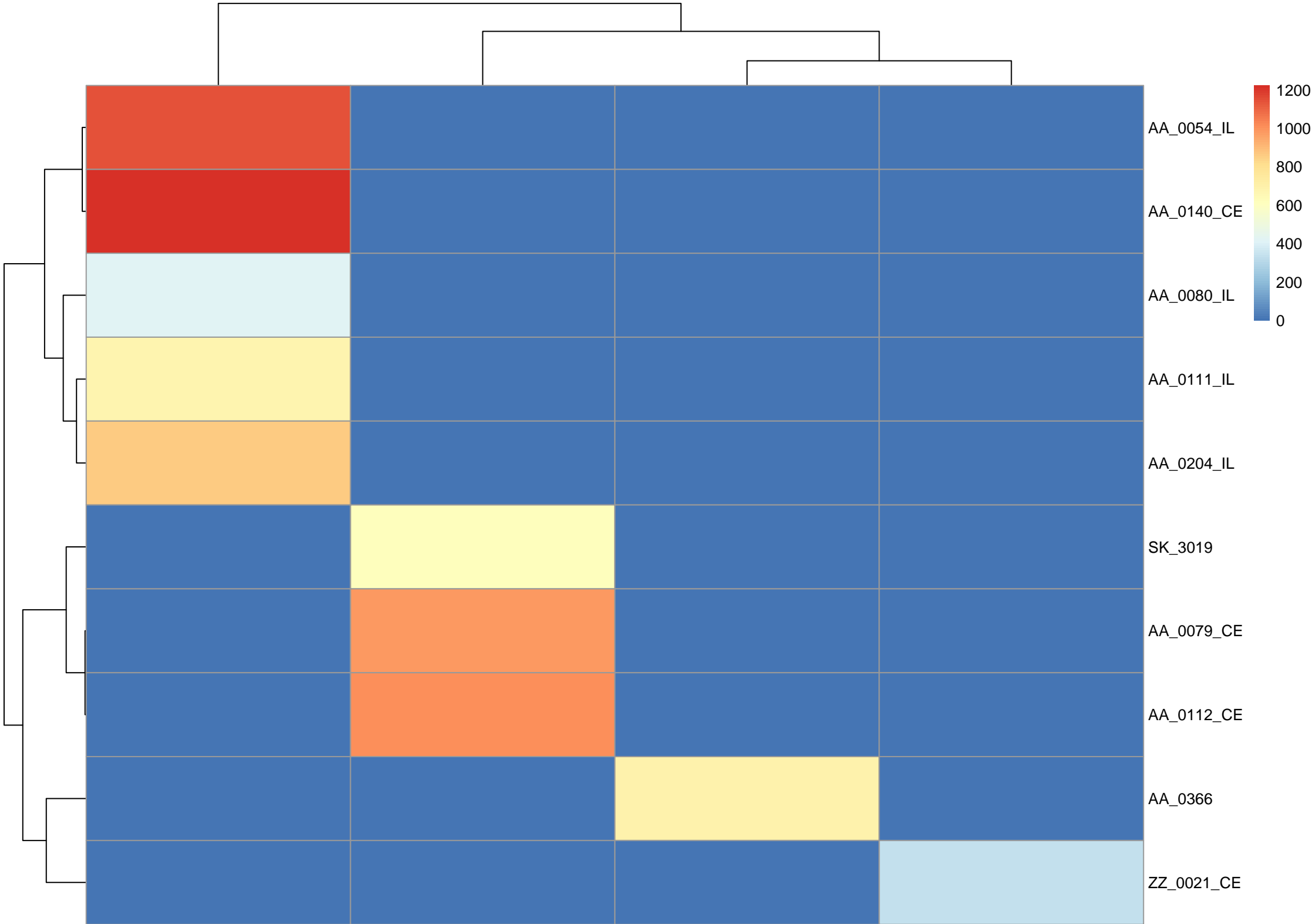

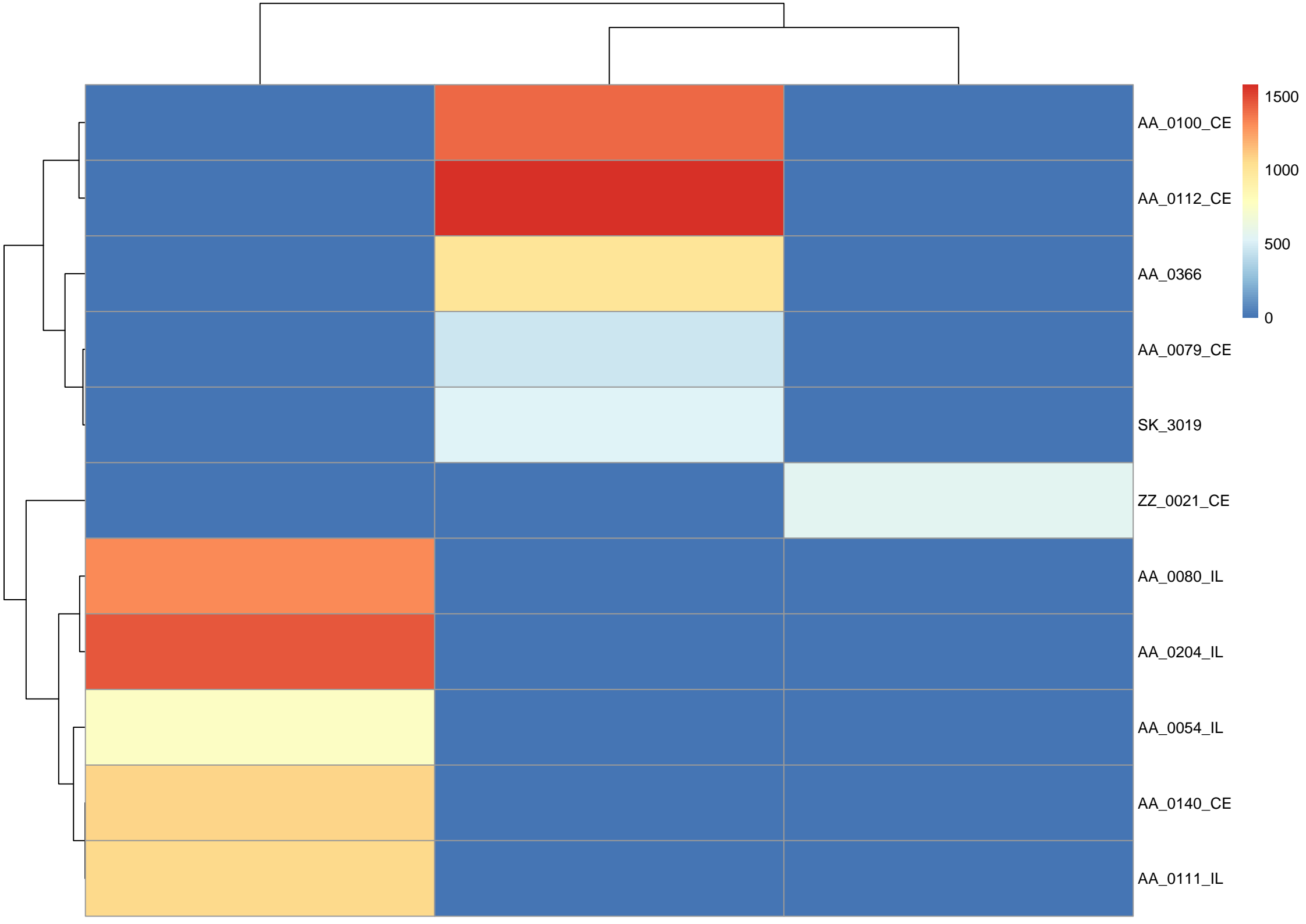

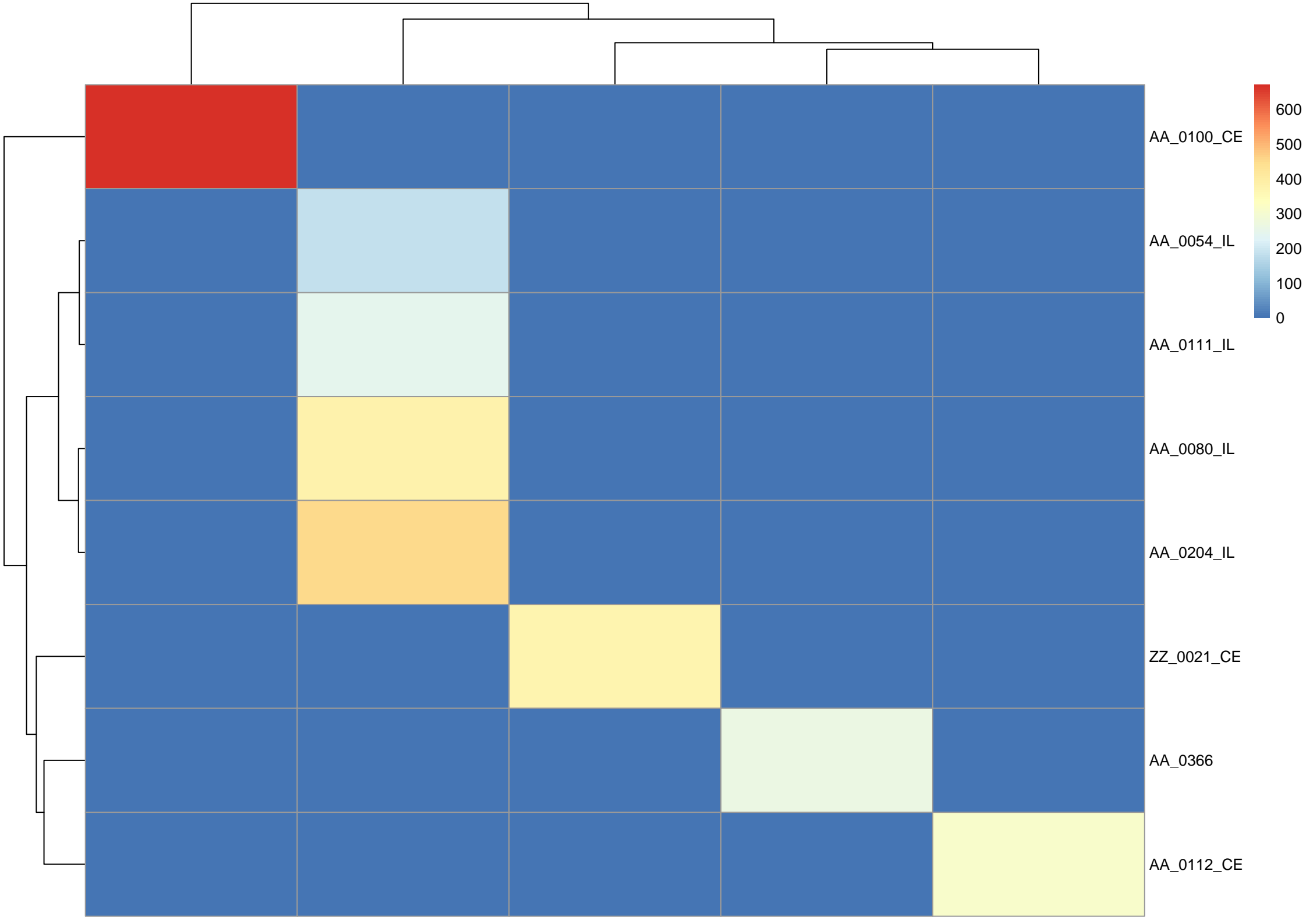

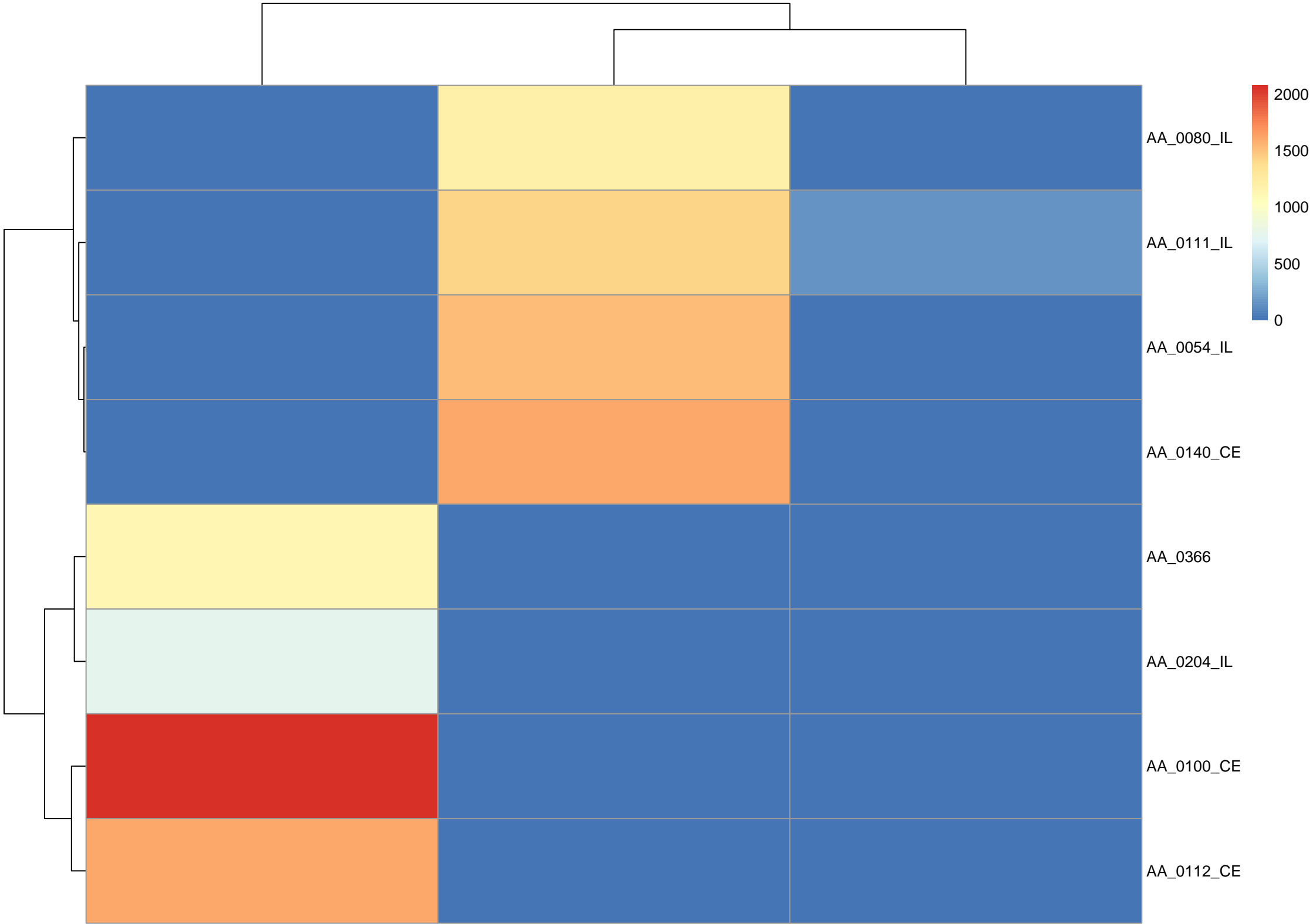

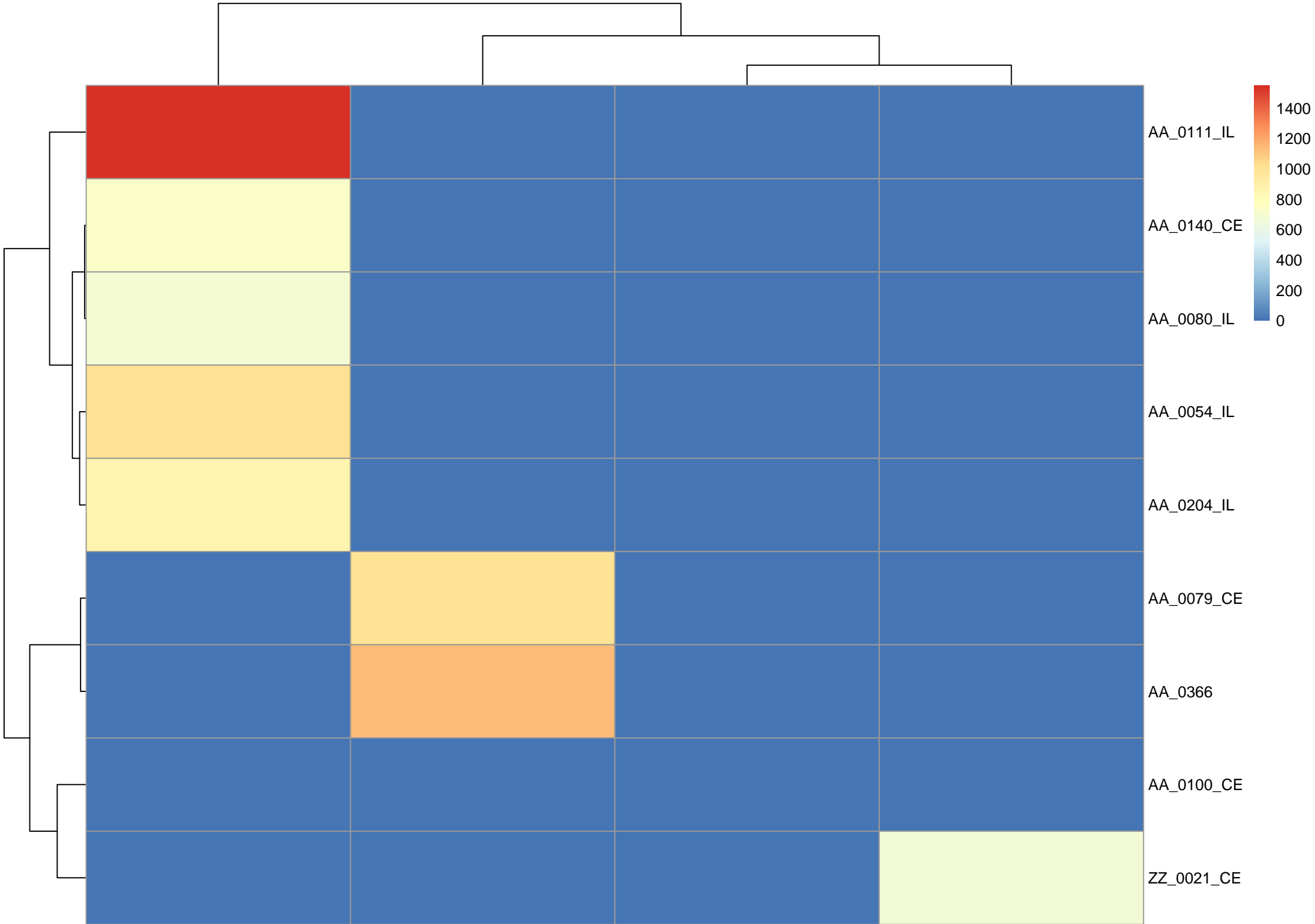

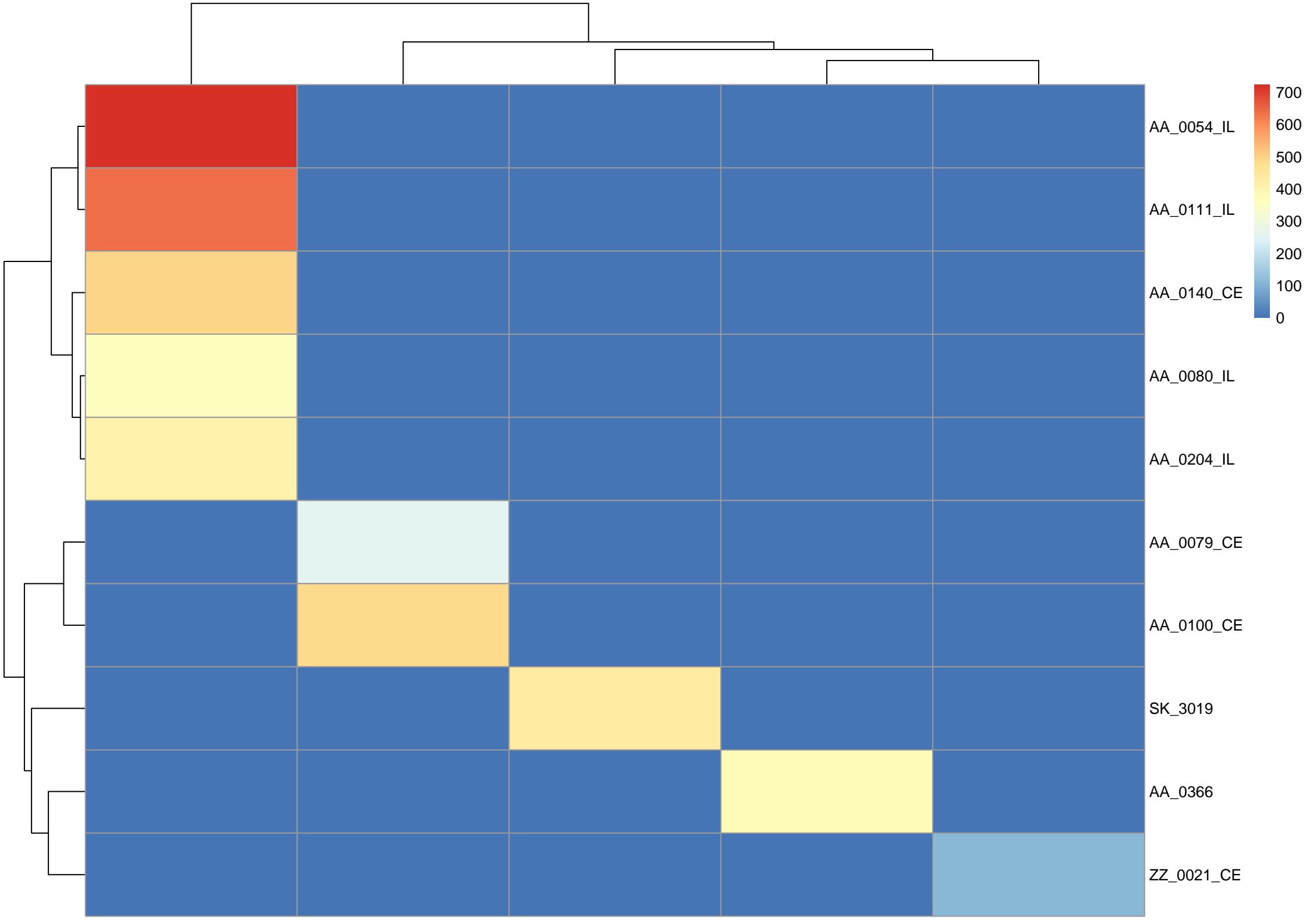

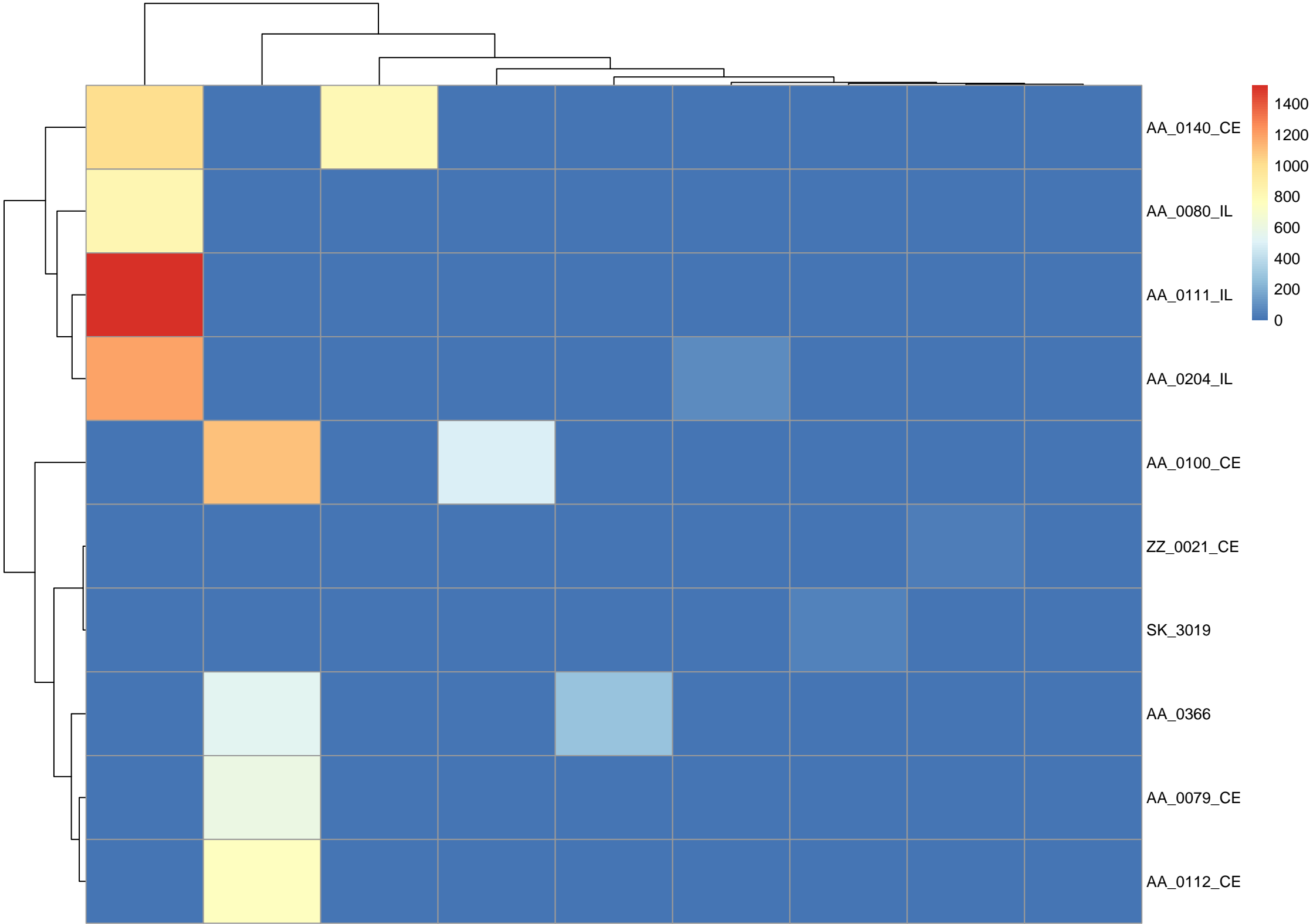

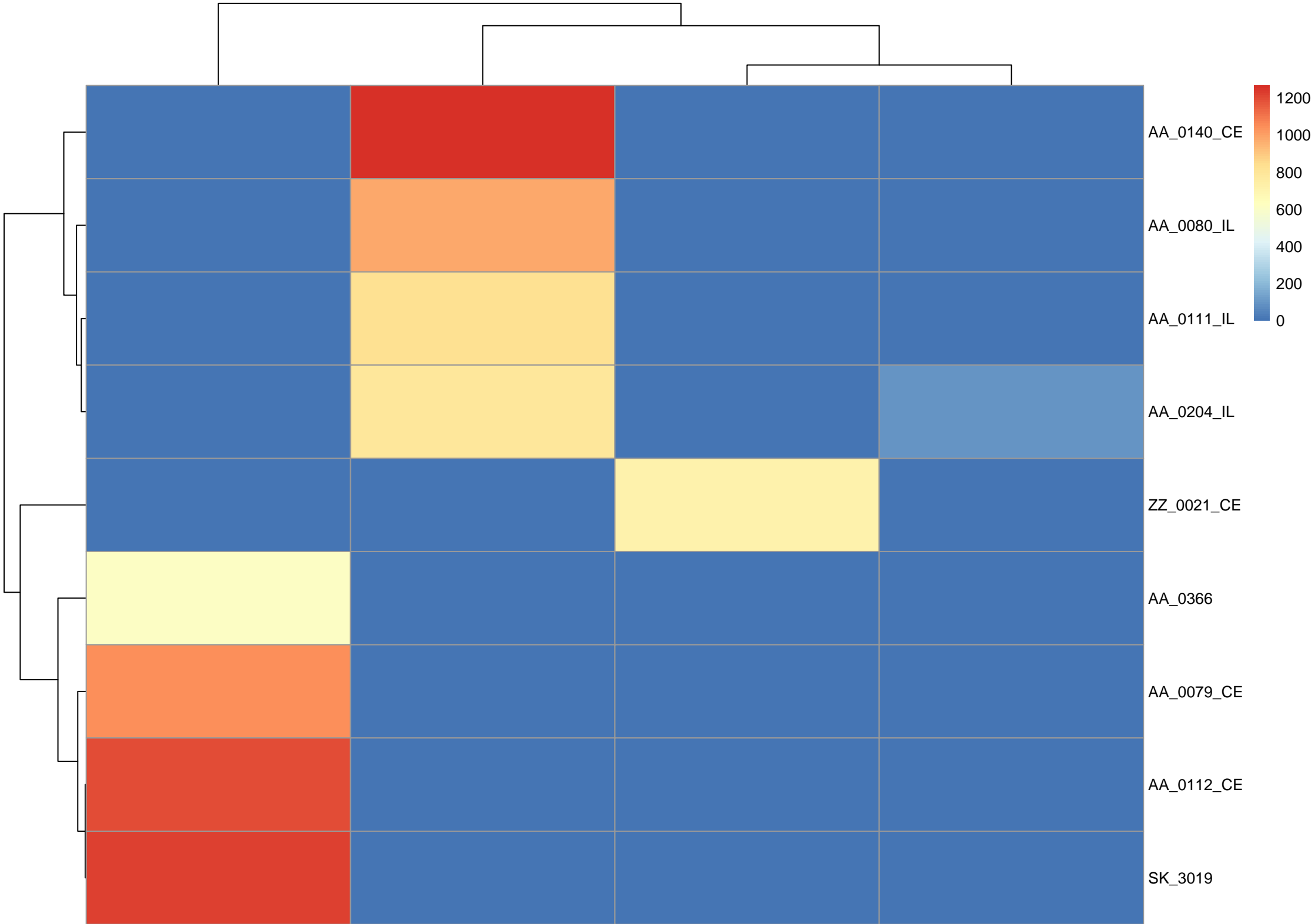

Supplement: Supplementary file 6 [file ECE3-10-1378-s006.pdf]

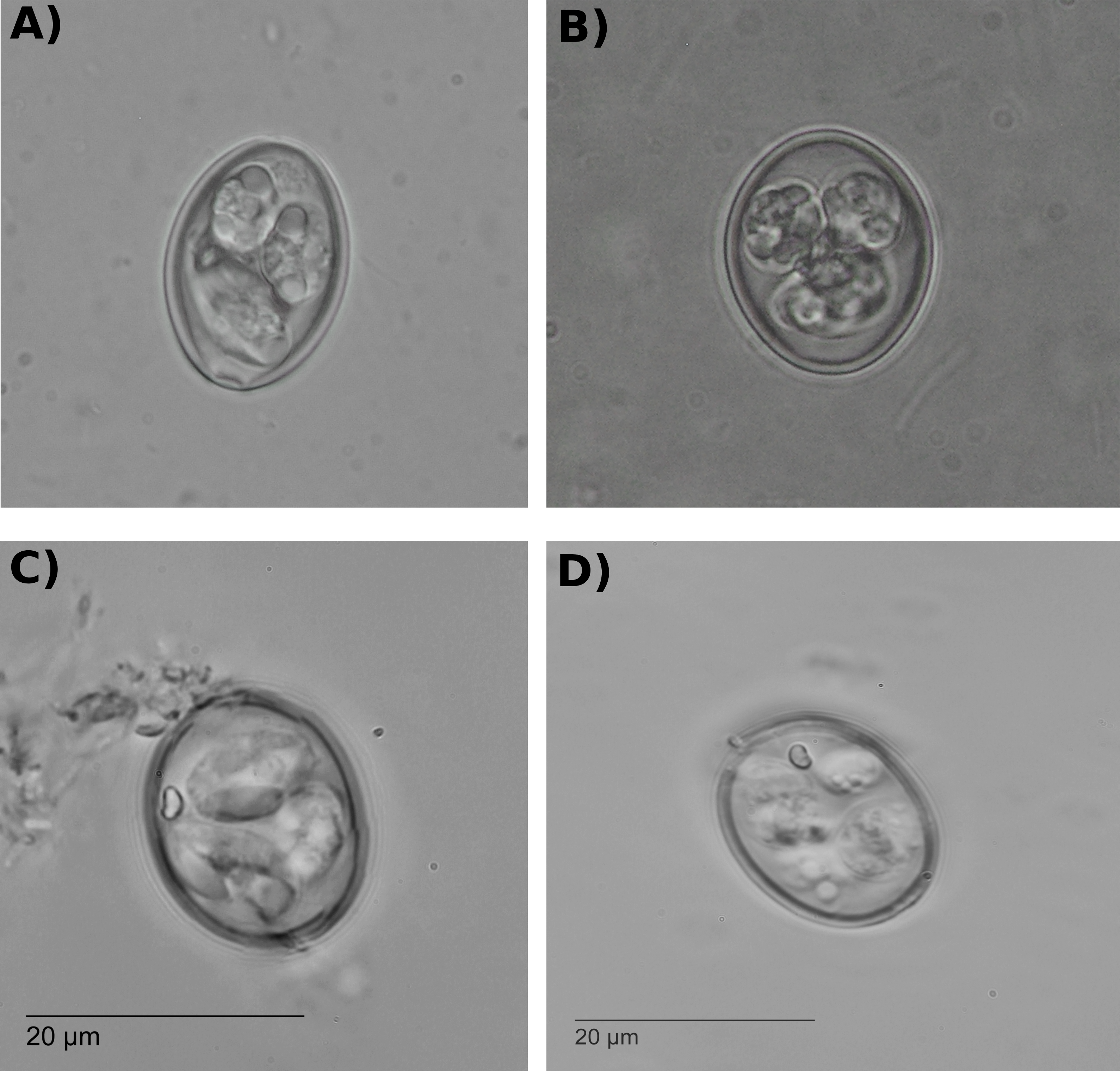

Supplement: Supplementary file 11 [file ECE3-10-1378-s011.tiff]

**A)**

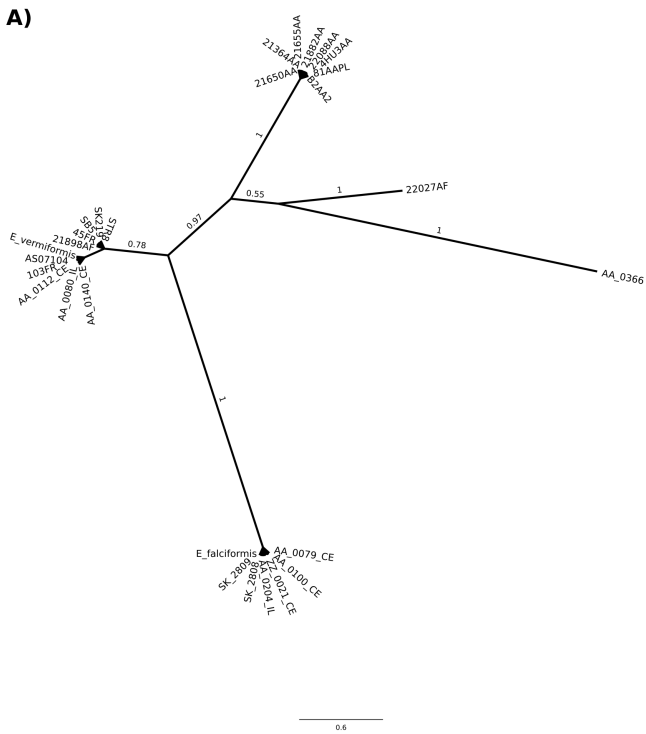

**B)**

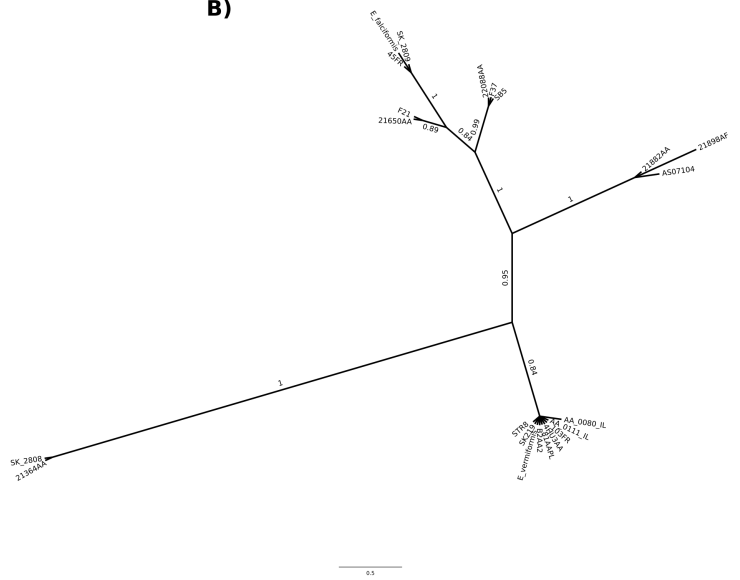

**C)**

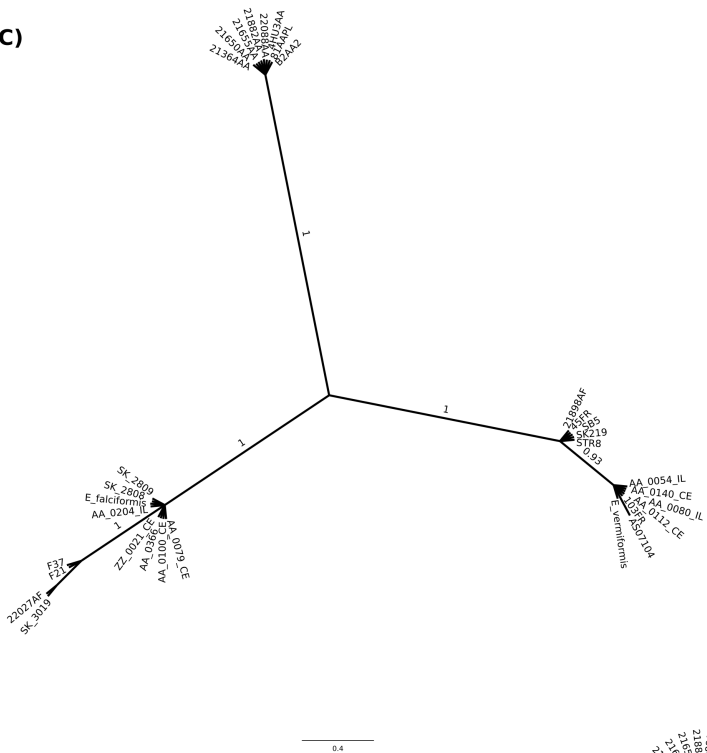

**D)**

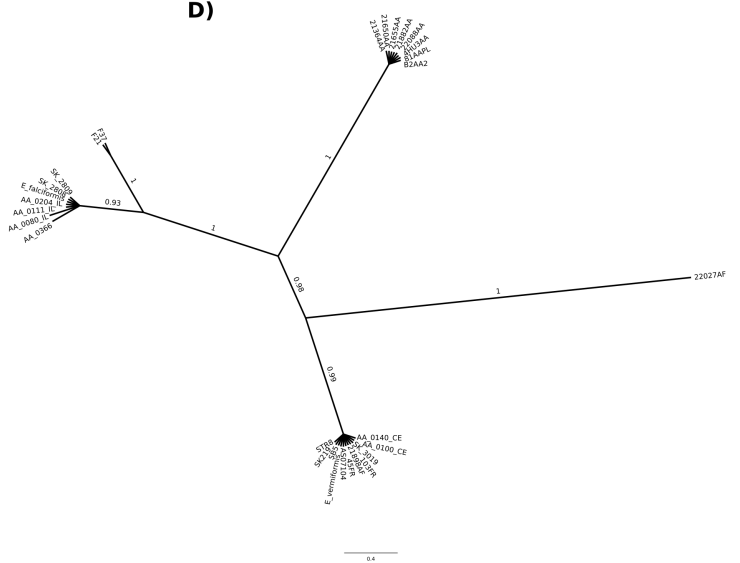

**E)**

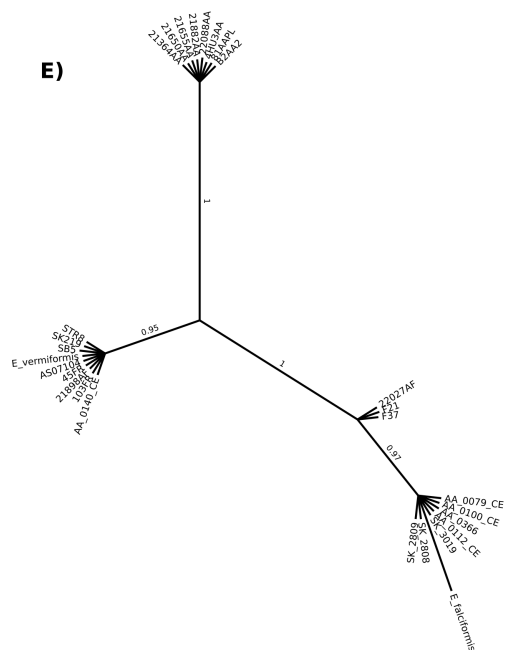

Supplement: Supplementary file 12 [file ECE3-10-1378-s012.pdf]
