# Supplementary material for: Generalist Eimeria species in rodents: Multilocus analyses indicate inadequate resolution of established markers
Source: Ecol Evol. 2020 Jan 11;10(3):1378–89. doi: 10.1002/ece3.5992 (PMC7029063; doi:10.1002/ece3.5992)
Supplement: Supplementary file 7 [file ECE3-10-1378-s007.pdf]

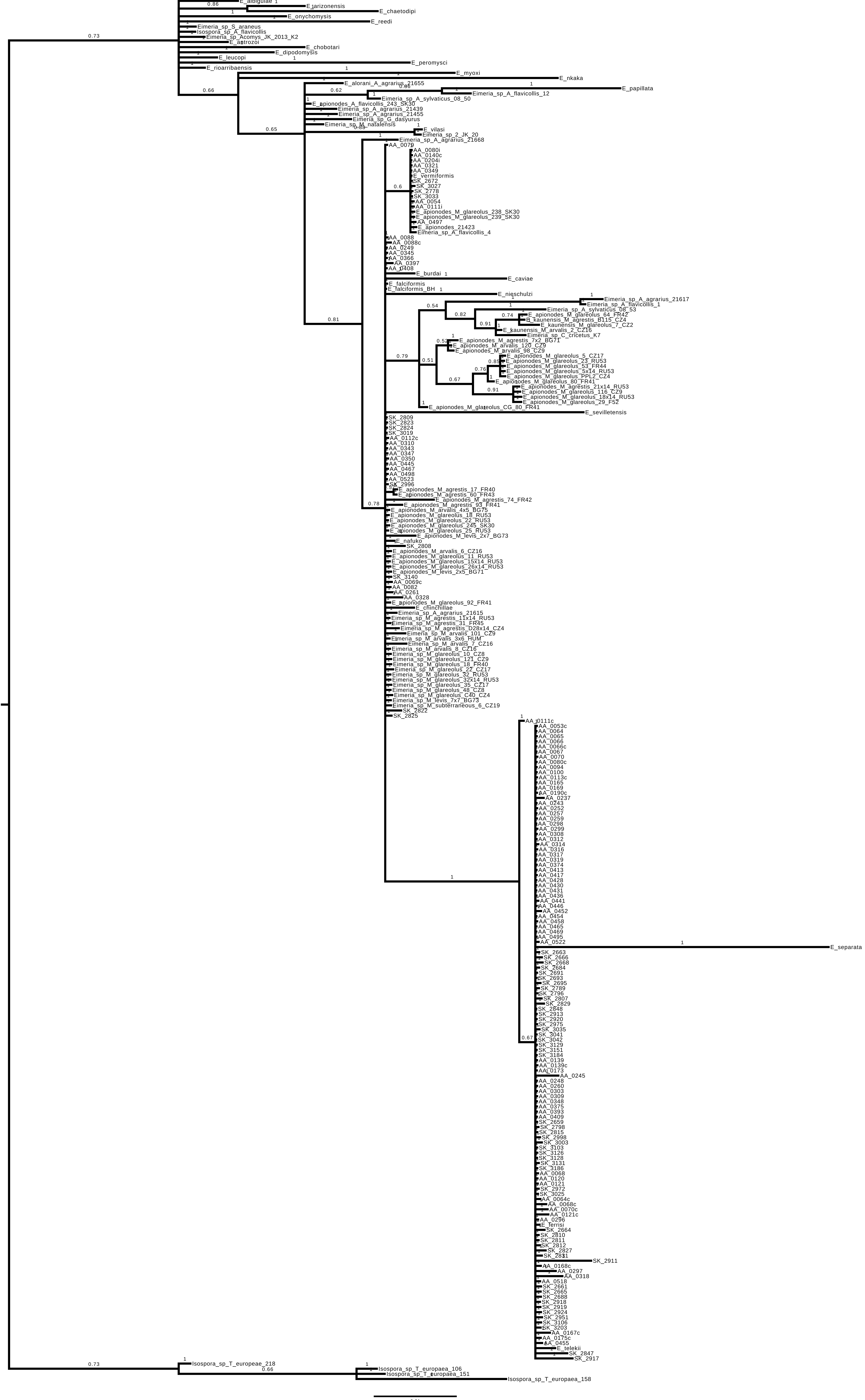

E_albigulae
E_arizonensis
E_chaetodipi
E_onychomysis
E_reedi
Eimeria_sp_S_araneus
Isospora_sp_A_flavicollis
Eimeria_sp_Acomys_JK_2013_K2
E_antrozoi
E_chobotari
E_dipodomysis
E_leucopi
E_peromysci
E_rioarribaensis
E_myoxi
E_nkaka
E_alorani_A_agrarius_21655
E_papillata
Eimeria_sp_A_flavicollis_12
Eimeria_sp_A_sylvaticus_08_50
E_apionodes_A_flavicollis_243_SK30
Eimeria_sp_A_agrarius_21439
Eimeria_sp_A_agrarius_21455
Eimeria_sp_G_dasyurus
Eimeria_sp_M_natalensis
E_vilasi
Eimeria_sp_2_JK_20
Eimeria_sp_A_agrarius_21668
AA_0079
AA_0080i
AA_0140c
AA_0204i
AA_0321
AA_0349
E_vermiformis
SK_2672
SK_3027
SK_2778
SK_3033
AA_0054
AA_0111i
E_apionodes_M_glareolus_238_SK30
E_apionodes_M_glareolus_239_SK30
AA_0497
E_apionodes_21423
Eimeria_sp_A_flavicollis_4
AA_0088
AA_0088c
AA_0249
AA_0345
AA_0366
AA_0397
AA_0408
E_burdai
E_caviae
E_falciformis
E_falciformis_BH
E_nieschulzi
Eimeria_sp_A_agrarius_21617
Eimeria_sp_A_flavicollis_1
Eimeria_sp_A_sylvaticus_08_53
E_apionodes_M_glareolus_64_FR42
E_kaunensis_M_agrestis_B115_CZ4
E_kaunensis_M_glareolus_7_CZ2
E_kaunensis_M_arvalis_2_CZ16
Eimeria_sp_C_cricetus_K7
E_apionodes_M_agrestis_7x2_BG71
E_apionodes_M_arvalis_120_CZ9
E_apionodes_M_arvalis_98_CZ9
E_apionodes_M_glareolus_5_CZ17
E_apionodes_M_glareolus_23_RU53
E_apionodes_M_glareolus_53_FR44
E_apionodes_M_glareolus_5x14_RU53
E_apionodes_M_glareolus_PPL2_CZ4
E_apionodes_M_glareolus_80_FR41
E_apionodes_M_agrestis_21x14_RU53
E_apionodes_M_glareolus_116_CZ9
E_apionodes_M_glareolus_18x14_RU53
E_apionodes_M_glareolus_29_F52
E_apionodes_M_glareolus_CG_80_FR41
E_sevilletensis
SK_2809
SK_2823
SK_2824
SK_3019
AA_0112c
AA_0310
AA_0343
AA_0347
AA_0350
AA_0445
AA_0467
AA_0498
AA_0523
SK_2996
E_apionodes_M_agrestis_17_FR40
E_apionodes_M_agrestis_60_FR43
E_apionodes_M_agrestis_74_FR42
E_apionodes_M_agrestis_93_FR41
E_apionodes_M_arvalis_4x5_BG75
E_apionodes_M_glareolus_18_RU53
E_apionodes_M_glareolus_22_RU53
E_apionodes_M_glareolus_245_SK30
E_apionodes_M_glareolus_25_RU53
E_apionodes_M_levis_2x7_BG73
E_nafuko
SK_2808
E_apionodes_M_arvalis_6_CZ16
E_apionodes_M_glareolus_11_RU53
E_apionodes_M_glareolus_15x14_RU53
E_apionodes_M_glareolus_26x14_RU53
E_apionodes_M_levis_2x5_BG71
SK_3140
AA_0069c
AA_0082
AA_0261
AA_0328
E_apionodes_M_glareolus_92_FR41
E_chinchillae
Eimeria_sp_A_agrarius_21615
Eimeria_sp_M_agrestis_11x14_RU53
Eimeria_sp_M_agrestis_31_FR45
Eimeria_sp_M_agrestis_D28x14_CZ4
Eimeria_sp_M_arvalis_101_CZ9
Eimeria_sp_M_arvalis_3x6_HUM
Eimeria_sp_M_arvalis_7_CZ16
Eimeria_sp_M_arvalis_8_CZ16
Eimeria_sp_M_glareolus_10_CZ8
Eimeria_sp_M_glareolus_121_CZ9
Eimeria_sp_M_glareolus_18_FR40
Eimeria_sp_M_glareolus_22_CZ17
Eimeria_sp_M_glareolus_32_RU53
Eimeria_sp_M_glareolus_32x14_RU53
Eimeria_sp_M_glareolus_35_CZ17
Eimeria_sp_M_glareolus_48_CZ8
Eimeria_sp_M_glareolus_C40_CZ4
Eimeria_sp_M_levis_7x7_BG73
Eimeria_sp_M_subterraneous_6_CZ19
SK_2822
SK_2825
AA_0111c
AA_0053c
AA_0064
AA_0065
AA_0066
AA_0066c
AA_0067
AA_0070
AA_0080c
AA_0094
AA_0100
AA_0113c
AA_0165
AA_0169
AA_0190c
AA_0237
AA_0243
AA_0252
AA_0257
AA_0259
AA_0298
AA_0299
AA_0308
AA_0312
AA_0314
AA_0316
AA_0317
AA_0319
AA_0374
AA_0413
AA_0417
AA_0428
AA_0430
AA_0431
AA_0436
AA_0441
AA_0446
AA_0452
AA_0454
AA_0458
AA_0465
AA_0469
AA_0495
AA_0522
E_separata
SK_2663
SK_2666
SK_2668
SK_2684
SK_2691
SK_2693
SK_2695
SK_2789
SK_2796
SK_2807
SK_2829
SK_2848
SK_2913
SK_2920
SK_2975
SK_3035
SK_3041
SK_3042
SK_3129
SK_3151
SK_3184
AA_0139
AA_0139c
AA_0173
AA_0245
AA_0248
AA_0260
AA_0303
AA_0309
AA_0348
AA_0375
AA_0393
AA_0409
SK_2659
SK_2798
SK_2815
SK_2998
SK_3003
SK_3103
SK_3126
SK_3128
SK_3131
SK_3186
AA_0068
AA_0120
AA_0121
SK_2972
SK_3025
AA_0064c
AA_0068c
AA_0070c
AA_0121c
AA_0296
E_ferrisi
SK_2664
SK_2810
SK_2811
SK_2812
SK_2827
SK_2831
SK_2911
AA_0168c
AA_0297
AA_0318
AA_0518
SK_2661
SK_2665
SK_2688
SK_2918
SK_2919
SK_2924
SK_2951
SK_3106
SK_3203
AA_0167c
AA_0175c
AA_0455
E_telekii
SK_2847
SK_2917
Isospora_sp_T_europeae_218
Isospora_sp_T_europaea_106
Isospora_sp_T_europaea_151
Isospora_sp_T_europaea_158
0.73
0.86
0.66
0.65
0.62
0.86
0.89
0.6
0.81
0.78
0.54
0.82
0.74
0.91
0.79
0.52
0.51
0.85
0.76
0.67
0.91
0.67
0.73
0.66
0.01
